# The engagement of psychiatrists in the assessment of euthanasia requests from psychiatric patients in Belgium: a survey study

**DOI:** 10.1186/s12888-020-02792-w

**Published:** 2020-08-08

**Authors:** Monica Verhofstadt, Kurt Audenaert, Kris Van den Broeck, Luc Deliens, Freddy Mortier, Koen Titeca, Koen Pardon, Dirk De Bacquer, Kenneth Chambaere

**Affiliations:** 1 End-of-Life Care Research Group, Vrije Universiteit Brussel (VUB) & Ghent University, Corneel Heymanslaan 10, 6K3, Ghent, Belgium; 2 Department of Public Health and Primary Care, Ghent University, Corneel Heymanslaan 10, 6K3, Ghent, Belgium; 3 Department of Psychiatry, Ghent University Hospital, Ghent, Belgium; 4 Collaborative Antwerp Psychiatric Research Institute (CAPRI), Antwerp University, Antwerp, Belgium; 5 Bioethics Institute Ghent, Ghent University, Ghent, Belgium; 6 Department of Psychiatry, General Hospital Groeninge, Courtrai, Belgium; 7 ULteam, end-of-life consultation centre, Wemmel, Brussels, Belgium

**Keywords:** Euthanasia, Mental disorders, Assisted suicide, Psychiatry, Survey study

## Abstract

**Background:**

Since its legalisation in 2002, the number of times euthanasia has been carried out in response to requests from adults with psychiatric conditions (APC) has continued to increase. However, little is known about why and how psychiatrists become engaged in the assessment of such euthanasia requests.

**Methods:**

A cross-sectional survey study was conducted between November 2018 and April 2019 of 499 psychiatrists affiliated with the Flemish Psychiatry Association. Chi square/Fisher’s exact tests were performed to examine if, and to what extent, psychiatrists’ backgrounds relate to their concrete experiences. The answers to the open question regarding motives for (non-) engagement were thematically coded.

**Results:**

Two hundred one psychiatrists participated, a response rate of 40%. During their careers, 80% of those responding have been confronted with at least one euthanasia request from an APC patient and 73% have become involved in the assessment procedure. Their engagement was limited to the roles of: referring physician (in 44% of the psychiatrists), attending physician (30%), legally required ‘advising physician’ (22%), and physician participating in the actual administration of the lethal drugs (5%). Within the most recent 12 months of practice, 61% of the respondents have been actively engaged in a euthanasia assessment procedure and 9% have refused at least once to be actively engaged due to their own conscientious objections and/or the complexity of the assessment. The main motive for psychiatrists to engage in euthanasia is the patient’s fundamental right in Belgian law to ask for euthanasia and the psychiatrist’s duty to respect that. The perception that they were sufficiently competent to engage in a euthanasia procedure was greater in psychiatrists who have already had concrete experience in the procedure.

**Conclusions:**

Although the majority of psychiatrists have been confronted with euthanasia requests from their APC patients, their engagement is often limited to referring the request to a colleague physician for further assessment. More research is needed to identify the determinants of a psychiatrist’s engagement in euthanasia for their APC patients and to discover the consequences of their non-, or their restricted or full engagement, on both the psychotherapeutic relationship and the course of the euthanasia request.

## Background

Since 2002, euthanasia – the intentional termination of life at the patient’s request – has been legal in Belgium, under strict conditions (see Box 1 and a flow chart that illustrates the standard legal euthanasia procedure in APC in OSF), including for Adults with Psychiatric Conditions (APC) [1]. APC encompass two adult patient groups: 1) patients whose euthanasia request is predominantly based on suffering caused solely by their psychiatric conditions, other than dementia; and 2) patients whose euthanasia request is predominantly based on suffering caused primarily by their psychiatric conditions and secondarily by somatic comorbid conditions.

However, euthanasia for APC patients is a highly controversial topic worldwide, and evokes strongly opposing views in the national and international media [2–5]. Extensive research is needed to clarify the way the Belgian Law on Euthanasia is put into practice for APC requesting euthanasia, and how Belgian psychiatrists deal with the roles and responsibilities associated with this practice.

The Belgian Law on Euthanasia places the psychiatrist in the role of gatekeeper, requiring the consultation and formal written ‘advice based on a formal assessment’ (formal advice, in short) by at least one psychiatrist for each request [1]. This psychiatrist is then engaged as a formally advising physician entrusted with the task of giving a formal advice regarding the patient’s (established or potential) eligibility for euthanasia. The formal advice can result in one of 3 determinations: the patient can be considered 1) eligible for euthanasia, 2) eligible for euthanasia, under certain conditions, or 3) not eligible for euthanasia.

To date, this seems to be what happens, as all reported cases include a formal advice from at least one psychiatrist [6]. Yet, a recent study demonstrated that, although a majority of Belgian psychiatrists are in favour of euthanasia as a legal end-of-life option for APC patients, only a minority are willing to actively engage in the assessments and procedures involved (see Box 2 for definitions of the roles a psychiatrist could be engaged in) [7]. Nonetheless, the number of reported euthanasia cases predominantly based on suffering caused by psychiatric conditions has increased steadily over time, although these cases still represent a small percentage of all euthanasia cases (i.e. 26 or 1.1% of all 2309 euthanasia cases performed in 2017) [8].

However, the practice of psychiatrists in APC euthanasia remains under-examined, and little is known about why and how psychiatrists become engaged in the assessment of a euthanasia request from an APC patient. Euthanasia in APC remains a matter of serious concern to society, and debates will remain purely theoretical until there is a solid scientific description of the empirical reality. Even if these requests are comparatively rare and seldom granted, they cannot remain unexamined on the grounds of their low rate of prevalence. Therefore, in order to assess the extent to which this issue pervades Belgian psychiatric practice, and to fill in the knowledge gaps described above, this study will address the following research questions:

1. During their career, to what extent have psychiatrists in Flanders and Brussels been confronted with, and engaged in, euthanasia requests from APC patients?
2. During the last 12 months, what proportion of these psychiatrists have been engaged in euthanasia assessment procedures in this patient group and in giving legally required advice? And what has been the nature of this advice?
3. What motives do psychiatrists in Flanders and Brussels cite in refusing or accepting engagement in the assessment of such procedures?
4. How does this engagement relate to their socio-demographic and professional background?

## Methods

### Study design

This cross-sectional study consisted of paper-and-pencil and web surveys on psychiatrists’ experiences with APC patients whose euthanasia requests are predominantly based on suffering caused by psychiatric conditions other than dementia.

### Participants

The surveys were launched among the professional body of psychiatrists affiliated with The Flemish Psychiatric Association (Vlaamse Vereniging voor Psychiatrie, FPA) *in order to gather original data from Flemish-speaking psychiatrists (N = 600).* Exclusion criteria were: 1) no work experience as a psychiatrist in adult mental healthcare, and 2) not currently working in Belgium. Taking the exclusion criteria into account, a sample of 499 psychiatrists were eligible to fill in the survey. The survey has been launched in the French-speaking part of Belgium, but results are excluded here as the response rate has been extremely low.

### Survey instrument

For this study, the survey questions on the psychiatrist’s concrete engagement in euthanasia cases based on psychiatric conditions were taken from a larger survey instrument, which is posted in the Open Science Framework repository (see Appendices A and B in OSF) accompanying this paper (in Dutch) and the Supplemental Materials in OSF (in French and English). The instrument was developed on the basis of five existing questionnaires [9–13], and adjusted to the context of current psychiatric clinical practice in Belgium.

This larger survey was tested for cognitive validation purposes (i.e. participants identifying potential problems with regard to item interpretation, item redundancy, *completeness of the survey*, feasibility to generate correct answers, and time estimation) via focus group analysis during a meeting with a heterogenous group (with regard to gender, age, and experience in euthanasia) of 15 psychiatrists [14]. Finally, the survey was revised and tested for time estimation and online technicalities by the broader research group (for more details, see the research protocol in OSF, Appendix C).

The survey questions were preceded by the following sentence: “Part 2: The following questions gauge your engagement in ADULT patients’ euthanasia requests that are PRIMARILY based on suffering CAUSED BY one or more psychiatric disorder(s), other than dementia.” The words in capitals were deemed necessary from a cognitive perspective, in order to avoid receiving data based on: 1) minors predominantly suffering from psychiatric conditions (as they cannot be considered eligible for euthanasia by law), and 2) adults suffering predominantly from somatic conditions and secondarily from psychiatric comorbid conditions.

For this specific study, the following 16 items of the larger survey (see OSF, Appendix D) were selected: 1) seven items concerning the psychiatrist’s personal and professional background; 2) two items on whether and why the psychiatrist agreed or refused to be engaged in euthanasia procedures concerning their own patients throughout their careers; and 3) six items on their specific role in euthanasia procedures during the past 12 months (see Box 2 in OSF for definitions of the roles a psychiatrist could be engaged in). One open question was checked for relevant additions to the answers that were provided.

### Procedure

*The FPA members* were invited to participate by e-mail. A link to LimeSurvey’s online platform [15] was included and the information letter was attached (see OSF Appendices E and F, in Dutch). According to the GDPR principle of adequate data processing management, a data manager was engaged only to coordinate data collection procedures. Anonymisation of data collection and data entry ensured that neither the data manager nor the researchers were able to trace which answers were given by which participant.

Non-responders received a first reminder via e-mail after 2 weeks. A second reminder, including a paper-and-pencil version of the questionnaire, was sent by post after 3 weeks.

Data were collected between November 2018 and April 2019. The data were imported from LimeSurvey into SPSS version 25, and cleaned according to the principles of a data analysis plan (Appendix G in OSF). The SPSS database was completed with data gathered from the returned paper surveys and cleaned.

As for the handling of missing data, it was determined beforehand that, in cases of too many missing answers (i.e. > 2 missing answers regarding background and > 3 missing regarding attitudes), all data from the respondent were excluded from analysis.

Personal and professional characteristics were illustrated by means of descriptive statistics and used as independent variables in statistical analyses. The answers on the open question regarding motives for engagement or not were thematically coded by means of identifying the main themes of the qualitative data, without a predetermined use of literature references nor background knowledge. Afterwards, the coded themes were ranked according to the frequency of its mentions.

Bivariate analyses (Chi square test) were performed to examine if, and to what extent, the psychiatrists’ backgrounds relate to their concrete experience of euthanasia cases based on psychiatric conditions. If the assumption for the Chi square test was violated, we used Fisher’s exact test. Due to the exploratory nature of our study, and in order not to miss out on potentially valuable findings that do not seem significant at first glance but are potentially valuable for further research, no correction test for multiple comparisons has been used. Confidence intervals for a population proportion were reported for the main findings. See Appendix G in OSF for the syntax used.

### Ethics

This research project received ethics approval from the Medical Ethics Committee of Brussels University Hospital with reference BUN 143201837302 and the Medical Ethics Committee of Ghent University Hospital with reference 2018–1165.

## Results

### Description of the sample

The FPA database consisted of 600 psychiatrists working in Flanders and Brussels. Of these, 499 are or have been professionally active as psychiatrists in psychiatric care for adult patients. The response sample consisted of 201 of these (valid response rate 40%). The data from 178 psychiatrists were found eligible for further analysis; data from 23 were excluded due to too many missing answers or the lack of explicit agreement regarding informed consent.

Some of the psychiatrists expressed their reasons for non-response as follows: bad timing (*n* = 2), not experienced in euthanasia in APC patients (*n* = 2), survey already filled in during cognitive testing (*n* = 1), not interested in the topic (*n* = 1), and never participate in surveys (n = 1).

Table 1 shows the characteristics of our sample. The majority were male (56%) and worked in a psychiatric hospital care facility (67%). Others worked mainly in private practice (45%) and/or in a community mental healthcare centre (12%). Most (48%) had more than 20 years’ experience, whereas 18% were trainees in psychiatry with less than 5 years of experience. 84% felt ready to engage in euthanasia procedures, and 50% felt sufficiently competent to do so. Only 5% had received specific training in medical end-of-life care.

**Table 1:** Psychiatrists’ demographics and professional characteristics (N/%)^a^

Variables	Sample (*N* = 178) (N^o^ and %)	
Gender		
Male	100	56.2
Female	75	42.1
Unknown	3	1.7
Age (in years)		
< 30	27	15.1
30–40 years	39	21.9
41–60 years	64	36.0
> 60	48	27.0
Worked as psychiatrist or psychiatric trainee during last year		
Yes	161	90.4
No	16	9.0
Unknown	1	0.6
Clinical setting^b^		
Private or Group Practice	80	44.9
Psychiatric Hospital Care	120	67.4
Community Mental HealthCare Center	22	12.4
Psychiatric Nursing Home	9	5.1
Psychiatric Home Care	6	3.4
Sheltered housing	12	6.7
Other^c^	26	14.6
Work experience (in number of years)		
< 5 years	32	18.0
6–10 years	20	11.2
11–20 years	41	23.0
> 20 years	85	47.8
Ever received special training in EOL		
Yes	9	5.1
No	167	93.8
Unknown	2	1.1
Readiness to be involved in euthanasia procedure(s)		
Yes	149	83.7
No	29	16.3

^a^In the online survey tool, explicit consent from the respondent had been asked by inserting the question “*Do you agree to take part in this survey?” immediately after the informed consent statement* and right before the start of the survey. If respondents clicked the option “no”, they have been sent directly to the ‘Non-response Questionnaire’ and only asked to clarify their motives for non-response. Hence, no other data (e.g. sex, work experience) was gathered nor included in this Table

^b^Close to 43% of the psychiatrists (76 out of 178) indicated to be professionally active in more than one workplace

^c^Other workplaces: prison or forensic psychiatric centers, psychiatric and psychosocial rehabilitation centers, psychiatric mobile crisis or response teams, other housing and care centers for other subpopulations (e.g. students, disabled persons)

### Psychiatrists’ experiences during their careers

As presented in detail in Table 2, during their careers, 80% of the responding psychiatrists (95% CI [74, 86]) have been confronted with euthanasia requests and procedures involving their own patients. Of these, 9% have at least once refused to be actively engaged in the assessment procedures, whereas 91% have never refused (data not shown in the Table).

**Table 2:** Engagement of psychiatrists in euthanasia, throughout their career

Confronted with euthanasia throughout their career	Sample (*N* = 178) N/%
Ever confronted with such requests	143 (80.3)
**-** Ever confronted and never refused to be involved	130 (73.0)
**-** Ever confronted, but ever refused to be involved	13 (7.3)
Ever engaged in assessment for euthanasia in APC patients^a^	
No, never	48 (27.5)
Yes, as	130 (72.5)^b^
**-** referring physician	78 (43.8)
**-** attending physician	54 (30.3)
with patients from my practice	41 (23.0)
for patients referred to me by a colleague	27 (15.2)
**-** preliminary advising physician	35 (19.7)
**-** formally advising physician	39 (21.9)
**-** participant to the administration of the lethal drugs (performing physician)	8 (4.5)
with patients from my practice	8 (4.5)
for patients referred to me by a colleague	0 (0.0)
- in another role^c^	12 (6.8)

^a^56 psychiatrists (43.4%) indicated that they have been actively engaged in more than one role, other than the role of referring physician. Seventy-one psychiatrists (55% of all 129 psychiatrists ever engaged in such euthanasia procedures) indicated that they have not been engaged in more than one role, throughout their career (46 psychiatrists as referring physician, 13 as attending physician, 7 as formally advising and 5 as preliminary advising physician)

^b^ One of the 130 cases was *not yet* involved

^c^12 psychiatrists indicated being involved in another role, most of them were *passively involved* as the treating physician of the patient’s psychopathology (e.g. discussing the euthanasia request during or after the euthanasia procedure, as well as during crisis confinement), as a member of the psychiatric care facility’s ethics committee or as trainee in psychiatry

73% (95% CI [66, 80]) of all participating psychiatrists have been actively engaged in the assessment of a euthanasia request from this patient group, 44% (95% CI [36, 51]) as referring physician (see the Glossary box for an overview and description). 56 (43% of all those ever engaged in a euthanasia assessment procedure) indicated that they have been actively engaged in more than one role other than that of referring physician (data not shown). A minority (23%) have engaged in the role of attending physician in the clarification of their own patient’s euthanasia request, and fewer (15%) have taken this particular role regarding a colleague’s patient.

22% of the responding psychiatrists reported experience in the role of formally advising physician, and 20% as preliminary advising physician. Fewer than 5% have assisted in the supply or administration of lethal drugs or have been present when a colleague-physician performed the act for their own patient. None reported any experience in this role regarding a colleague’s patient.

### Psychiatrists’ experiences during the past 12 months

During the previous 12 months, 61% (95% CI [53, 67]) have been actively engaged in a specific role regarding the assessment of a euthanasia procedure for an adult psychiatric patient (Table 3). Among these, 70% have been actively engaged in one or two procedures based on psychiatric conditions, and 8% in more than five.

**Table 3:** Psychiatrists actively engaged in euthanasia cases during the previous 12 months

	N/% of all engaged psychiatrists	% total sample (*N* = 178)
How many psychiatrists were engaged in any role?	108 (100%)	60.6%
1. In 1–2 euthanasia procedures	76 (70.4%)	42.7%
2. In 3–5 euthanasia procedures	24 (22.2%)	13.5%
3. In > 5 euthanasia procedures	8 (7.4%)	4.5%
How many psychiatrists were asked to be engaged as formally advising physician?	102 (96.2%)	57%

96% (or 57% of the total sample) have been engaged as formally advising physician during the 12 months prior to the survey; 70% were engaged in not more than two cases. Although asked to give formal advice, 18.6% have refused to do so. More detailed information is shown in Table 4.

**Table 4:** Psychiatrists engaged as “formally advising physician” during the past 12 months

Type of engagement	In 1–2 euthanasia procedures	In 3–5 euthanasia procedures	In more than 5 euthanasia procedures	Total
Giving any advice	76 (100%)	20 (100%)	6 (100%)	**102 (100)**
Giving Formal Positive Advice	36 (47.4%)	3 (15%)	2 (33.3%)	41 (40.2%)^a^
Giving Formal Negative Advice	26 (34.2%)	14 (70%)	2 (33.3%)	42 (41.2%)^b^
Refusing to give Formal Advice	14 (6.3%)	3 (15%)	2 (33.3%)	19 (18.6%)^c^

^a^of which 16 psychiatrists (39%) only gave positive advices

^b^of which 17 psychiatrists (40%) only gave negative advices

^c^of which 10 psychiatrists (52.7%) only refused to give advices

### Main motives for refusing or accepting engagement

All of the psychiatrists were asked whether they had ever refused active engagement in the euthanasia procedure of their own patient. Table 5 shows the main motives they cited for refusal. The most reported motives were: fundamental objections to euthanasia in APC; the difficulties in adequately assessing the – according to some, unclear and/or subjective – legal criteria; difficulties in reconciling euthanasia assessment within the therapeutic relationship; the ineligibility of the APC patient’s request, as it had been expressed prior to euthanasia legislation. Other reported motives included the perceived ineligibility of the APC patient’s euthanasia request; complexity of the patient’s current life circumstances (e.g. young age and complex family situation); the psychiatrist’s perception of being insufficiently competent to engage in such procedures; and previous experiences with APC patients who had withdrawn their request (e.g. unexpected rehabilitation).

**Table 5:** Motives for (not) refusing to be engaged in euthanasia assessment procedures regarding APC (sort by frequency)

Motives for refusing to be engaged in psychiatric euthanasia procedures^a^	
1. Fundamental motives	
Fundamental objections against euthanasia regarding psychiatric patients (ethical, moral, deontological reasons)	
Euthanasia is incompatible with therapeutic relationship, but should be topic for further exploration in life track	
Physicians should never give the sign to the patient of giving up hope	
Law needs to be re-examined as criteria are unclear or need to be further restricted for this patient group	
In that specific time, the euthanasia law was not yet effective	
2. Ineligibility of the patient’s euthanasia request	
Treatment options were still left, including non-medical treatment	
Substantive legal criteria were not fulfilled	
Personality disorder as contra-indication	
3. Complex circumstances	
Patient’s complex family situation	
Patient’s young age	
Not enough knowledge on the patient and her situation	
Not enough competence to get actively involved	
4. Experience of rehabilitation with former patients with withdrawn request	
Motives for accepting to be engaged in euthanasia procedures concerning psychiatric patients^a^	
1. Fundamental motives	
Fundamental right of the patient to ask for euthanasia	
Fundamental task of the psychiatrist to take and discuss the request seriously	
Opportunity to keep on searching for underlying meaning request and treatment options	
2. Eligibility of the patient’s euthanasia request	
Unbearable and untreatable suffering do exist	
Specific task of the psychiatrist to be involved in the assessment	
The euthanasia request is always based on misfortunes in many more domains in life	

^a^These motives result from 65 psychiatrists’ answers to the open ‘What was your motive to (not) refuse to be actively engaged in the clarification of the patient’s euthanasia request?

Alternatively, motives for accepting involvement mostly concerned the APC patient’s right to request euthanasia; the psychiatrist’s expertise in exploring, and duty to explore, the meaning of the request and to assess all legal criteria; the possibility that a serious discussion would serve as a therapeutic tool, facilitating further explorations of alternatives to death. In addition, it was stated that an APC patient can be eligible for euthanasia not only due to their poor medical condition but also because of the accumulation of the many misfortunes they had encountered in life.

### Psychiatrists’ engagement related to their socio-demographic and professional background characteristics

Table 6 represents the relation between the psychiatrists’ characteristics and their prior engagement in euthanasia procedures concerning APC. There was more perception of being sufficiently competent to engage in euthanasia procedures in those who had taken up a specific role in euthanasia procedures concerning APC (*χ*^*2*^_*(1177)*_ = 10.487, *p* = .001), including a role as preliminary (*χ*^*2*^_*(1177)*_ = 7.803, *p* = .008), formally advising (*χ*^*2*^_*(1177)*_ = 23.586, *p* < .001), or attending physician (*χ*^*2*^_*(1177)*_ = 28.801, *p* < .001) and – according to the Fisher exact test – also as performing physician (*p* < .001).

**Table 6:** Socio-demographic and professionals factors in psychiatrists’ engagement in the euthanasia decision-making procedure

	Ever performed the role of …					Performing physician
	NO ROLE	Referring physician	Preliminary advising physician	Formal advising physician	Attending physician	
Sex						
Male (*n* = 100)	25 (25)	43 (43.9)	16 (16.3)	27 (27)	34 (34)	5 (5)
Female (*n* = 75)	24 (32)	33 (44)	17 (22.7)	12 (16)	19 (25.3)	3 (4)
Age						
< 40 (*n* = 66)	23 (34.8)	32 (48.5)	12 (18.2)	**4 (6.2)**^a^	**12 (18.2)a**	2 (3)
41–60 (*n* = 64)	14 (21.9)	32 (50)	11 (17.2)	**18 (28.1)**^a^	**23 (35.9)a**	4 (6.3)
> 60 (*n* = 48)	12 (25.5)	14 (29.2)	12 (25)	**17 (36.2)**^a^	**19 (39.6)a**	2 (4.2)
Years of work experience						
< 10 (*n* = 52)	20 (38.5)	24 (46.2)	10 (19.2)	**4 (7.7)a**	**8 (15.4)a**	1 (1.9)
10–20 (*n* = 41)	9 (22)	19 (46.3)	11 (26.8)	**8 (19.5)a**	**15 (36.6a)**	4 (9.8)
> 20 (*n* = 85)	20 (23.8)	35 (41.2)	14 (16.5)	**27 (31.8)a**	**31 (36.5)a**	3 (3.5)
Perceived Competence						
Yes (*n* = 89)	**15 (16.9)**^b^	37 (41.6)	**25 (28.1)a**	**33 (37.1)**^b^	**43 (48.3)**^b^	**8 (9)**^b^
No (*n* = 88)	**34 (38.6)**^b^	41 (46.6)	**10 (11.4)a**	**6 (6.8)**^b^	**10 (11.4)**^b^	**0**^b^

In bold: *p* < .05

^a^In bold: significant results after Bonferroni test for multiple comparisons, *p* < .0033

^b^In bold: significant results after Bonferroni test for multiple comparisons, *p* < .005

In addition, more years of work experience and higher age were significantly associated with more experience in the roles of formal advising physician (*χ*^*2*^_*(2178)*_ = 7.506, *p* = .023 for work experience and *χ*^*2*^_*(2178)*_ = 16.253, *p* < .001 for age range) and attending physician (*χ*^*2*^_*(2178)*_ = 7.772, *p* = .021 for work experience and *χ*^*2*^_*(2178)*_ = 11.106, *p* = .004 for age range). No significant associations were found based on biological sex. Years of work experience with regard to the role of formally and/or attending physician nor the age range with regard to the role of attending physician did survive Bonferroni correction for multiple comparisons.

## Discussion

### Summary of results

Over their careers*,* 4 out of 5 of the psychiatrists have been confronted with a request for euthanasia, predominantly based on suffering caused by their APC patient’s psychiatric condition(s), and 7 out of 10 have engaged in the assessment of the request, as referring physician (44%), as attending physician (30%), as formally advising physician (22%), or as performing physician (5%). Over the previous 12 months, 3 out of 5 have been actively engaged in an assessment, 96% as formally advising physician.

Over their careers, 1 in 10 have at least once refused to be actively engaged in an evaluation procedure, due to their own conscientious objection and/or the complexity of assessment in this patient group. The main motive for engagement in euthanasia assessment procedures is the view that the patient has a fundamental right to request and the psychiatrist has a duty to respect and assess these requests.

The perception of being sufficiently competent to engage in euthanasia procedures in this patient group was more common in psychiatrists who have had concrete engagement experiences.

### Strengths and limitations

The results of this study cannot readily be generalized and must therefore be interpreted with caution. Although we achieved higher response than anticipated in this target group, only a minority (40%) of the FPA-affiliated members completed and returned the questionnaire. A strength of this study is the inclusion of a representative group of APC patients, as the psychiatrists were able to fill in the questionnaire with the following two APC groups in mind: 1) patients whose euthanasia requests were solely prompted by their psychiatric conditions, and 2) patients whose euthanasia requests were primarily prompted by suffering caused by their psychiatric conditions, and secondarily by suffering caused by somatic comorbid conditions. We are well aware that some psychiatrists – positioned at either end of the euthanasia debate – may not have participated in the survey as they are not FPA-affiliated (around 10–20% of all psychiatrists working in Flanders are not FPA-affiliated) or because they are opposed to the study and its set-up (e.g. fearing potential criticism of arguments pro or contra today’s euthanasia law and/or practice). Therefore, we cannot exclude the risk of self-selection and response bias skewing the estimates of our survey. In addition, the findings only relate to the Flemish part of Belgium. Unfortunately, a similar survey among French-speaking psychiatrists was unsuccessful and hence we cannot report on this part of the Belgian practice, where previous research shows that requests for euthanasia are dealt with quite differently [16].

In order to facilitate comparison across countries with comparable euthanasia legislation, our questionnaire closely followed the pre-existing Dutch questionnaire in terms of item formulation. Therefore, cognitive testing of the questionnaire was conducted during one focus group session with psychiatrists and their trainees, and not by means of in-depth cognitive interview techniques on an individual level, which might have caused bias. Finally, no established qualitative methods were used to analyse the – concisely written – data. A future follow-up study could make use of these established methods (e.g. literature references).

### Interpretation of findings

Our results suggest that psychiatrists in Belgium need to be well informed about the euthanasia law and the assessment procedure, as a high proportion of them have been confronted with such a request. Even if all of the non-responders have never been confronted with such a request, one-fourth of all affiliated FPA-members still have been. Our study revealed that 80% reported that they have been confronted with a request at least once in their career. Among them, 7% had at least once refused to actively engage in a euthanasia assessment, which means that euthanasia assessments concerning APC involve a larger proportion of psychiatrists than commentators often presume. This is in line with a previous study, based on the same survey, of these psychiatrists’ attitudes towards euthanasia in the APC patient group and their readiness to engage in these procedures: that study revealed that a majority are not only in favour of euthanasia as a potential end-of-life option in this patient group, but that they are also willing to be actively engaged in the procedure [7].

However, their engagement is mainly restricted to the role of referring physician. This is probably due to the complexity of the euthanasia assessment procedure, which involves other colleagues (not necessarily restricted to the medical discipline of psychiatry) and the assessment of different domains: i.e. the difficulty of interpreting and assessing all legally due care criteria in this patient group, the difficulty of reconciling a euthanasia assessment with the therapeutic relationship, and concern about inadequate approaches towards euthanasia assessment and the lack of safeguards in current euthanasia practice [7]. This might also be due to the fear of potential juridical prosecution. In that regard, a number of guidelines and a deontological code have been published recently (2017–2019) in order to support psychiatrists in adequately managing euthanasia assessment. The question is to what extent psychiatrists are already familiar with these guidelines and codes and to what extent they deem them sufficiently useful.

Nevertheless, ‘referral’ is a minimal engagement that is also embedded in the Belgian Board of Physicians’ deontological code, even in cases of conscientious objection. The physician’s legal right to refuse engagement in euthanasia procedures is limited due to the patient’s legal right to be informed clearly and in a timely manner of the reasons for refusal and to be referred to a colleague physician (not necessarily a psychiatrist) for the further clarification of their request [17]. In that respect, it is noteworthy that some psychiatrists cite conscientious objection as a motive for non-referral. On the other hand, some may also sidestep the referral requirement because of a lack of knowledge of this legal criterion or its vagueness, as neither the law nor the existing guidelines provide a sufficiently adequate definition of the term ‘referral’, let alone ‘effective referral’ (cf. patients being given the run-around).

Apart from conscientious objection, the fact that the majority of the responding psychiatrists have been engaged only in a referring role might also be due to the fact that they have not been specially trained in euthanasia consultation and practice, and also that one-fifth were working as trainees at the time and were not allowed to act as an advising or attending physician.

Reluctance to actively engage as attending, formally advising and/or performing physician has also been confirmed in Dutch evaluation studies, which have revealed that APC patients’ euthanasia requests are seldom granted, and even those that are granted do not automatically result in the actual performance of euthanasia [9, 13]. Former Belgian and Dutch studies attribute this reluctance to the complexity of this specific practice in terms of the difficulties psychiatrists have in determining whether the APC patient meets all legal and due care criteria – with regard to, for example, their mental capacity and the incurability of their disorder (given the unpredictable prognoses and outcomes of psychiatric disorders) [18–21] – as well as in integrating a euthanasia request within the therapeutic relationship [7, 22].

### Implications for practice, policy and research

Some of the results regarding conscientious objection and non-referral confirm that, after nearly two decades of legalized euthanasia, it remains a decidedly difficult situation for psychiatrists. More insight is needed to clarify when and why such a referral ends up with the formally advising psychiatrist denying the request, as well as when and why the request is eventually granted. Furthermore, it remains largely unknown what involvement in a euthanasia assessment means for the psychotherapeutic relationship – does it lead to discouragement, demotivation or even despair, when an APC patient learns that their euthanasia request was not taken seriously, let alone granted, and hence the risk of suicide increases? On the other hand, does the option of euthanasia itself undermine the APC patient’s sense of hope and trust in therapy and distract their attention from therapeutic and other options of care that might otherwise be offered?

The fact that psychiatrists are more actively engaged in euthanasia procedures when they perceive themselves as competent in the subject indicates a need to evaluate and reflect on potential thresholds or shortcomings in currently available training and support initiatives as well as in a handful of recently published (and insufficiently known?) advising guidelines [23–27]. As these initiatives take a different, and often more restrictive, approach than is required by law (e.g. by stipulating that at least two positive advices should be obtained from at least two psychiatrists, instead of two advices from at least one psychiatrist of which the outcome is not binding), this may lead to unequal treatment of euthanasia requests and/or an unequal course in euthanasia procedures. As these guidelines are not binding, they might have the undesirable consequence that an APC patient’s euthanasia request is handled differently according to individual differences in physicians’ approaches towards euthanasia assessment and decision-making (whether or not the physician involved also takes the more stringent criteria of the guidelines into account). As a result, this may have an additional undesirable consequence: the patient might immediately search for physicians who presumably hold more permissive stances and approaches regarding euthanasia instead of discussing their euthanasia request with the treating psychiatrist, under the assumption that the latter is inclined to take a more restrictive stance [28]. Therefore, more in-depth research on what kind of additional support and specific training psychiatrists need regarding the adequate and proper handling of a euthanasia request is recommended.

In addition, further qualitative research should investigate what (non-)referral exactly entails when psychiatrists refuse to engage (e.g. refusing to even discuss euthanasia as an end-of-life option, to refusing to actively engage in a role other than a referring one but remaining open to a sound debate with the patient regarding euthanasia). This can have a great impact on the therapeutic relationship, whether or not the patient’s euthanasia request and procedure can still be openly discussed in therapeutic consultations by reciprocally sharing information, concerns and emotions, even when patient and physician have different perspectives, or even different values, regarding medical end-of-life decisions. Just as active euthanasia assessment and decision-making requires excellent communication skills from all physicians involved [22], open discussions about euthanasia can be very demanding, and even burdensome, on an APC patient’s treating psychiatrist on a cognitive as well as an emotional level. As previous research has revealed that APC patients’ euthanasia requests are less likely to be granted than those prompted by purely somatic conditions, the APC patients’ treating psychiatrists should also be sufficiently empowered to deal with their patients’ emotions after obtaining negative advices, and especially after a conditional or definitive refusal [13]. The scarce literature on this topic has revealed that very few treating physicians discuss or evaluate the patient’s death ideation or situation after a refusal [29]. Therefore, it would be interesting to examine whether this also applies to psychiatrists. In addition, research is needed on whether existing courses on medical end-of-life decisions sufficiently address communication techniques for all actively engaged physicians as well as all psychiatrists handling their own patients’ euthanasia procedure, from the moment of the APC patient’s first request for euthanasia to the final decision. Moreover, more research is needed to determine whether these courses sufficiently address the ethical value-based aspects of medical end-of-life decisions in addition to the practical clinical, juridical and technical aspects. As for the ethical aspects, insight is needed into whether the ethical principles for guiding good medical practice – e.g. respect for the patient’s autonomy, promotion of what is best for the patient versus avoiding harm – are sufficiently interlarded with arguments and counter-arguments based on empirical data, case comparison and thought experiments.

Furthermore, more government-coordinated initiatives could be established (e.g. an optimised budget for more centralised training courses and often-repeated evaluation studies following the example of the Dutch quinquennial ones). That said, it must be stressed that, like other new medical practices, factors such as time and experience can also contribute to competence-based practice. This could increase the knowledge and transparency of the entire practice, providing an opportunity to detect and resolve potential shortfalls, and hence offer sufficient medico-legal protection to all actors involved. Future research should also emphasize the perspectives of all actors (including the APC patients and their carers, friends and family) in order to gain more insight into euthanasia practice concerning APC patients as a whole.

Finally, as a previous study based on this survey has revealed that the younger generation of psychiatrists is more supportive of euthanasia in APC patients and more willing to be actively engaged [30], future research endeavours might also reveal a potential cohort effect in terms of psychiatrists’ concrete experiences and engagement in psychiatric euthanasia assessment.

## Conclusions

In their clinical practice, many of the psychiatrists studied have been confronted with requests for euthanasia by adults with psychiatric conditions (APC). However, their engagement is often limited to referring to a colleague-physician for the assessment and possible granting of the request. The assessment of the legal due care criteria stated in the euthanasia law in Belgium seems to be difficult to apply to this specific patient group and it is probably difficult to reconcile within a therapeutic relationship.

More research is needed to identify the determinants of psychiatrists’ decision not to personally engage in a role other than referring the APC, on the latter’s request, to a colleague-physician willing to engage more fully in the assessment of euthanasia requests (e.g. moral objections, the need for more objective euthanasia assessment approaches, wanting to avoid sending the message of giving up on the patient in order to maintain therapeutic compliance and effectiveness, etc). In addition, this can illuminate both the positive and negative consequences of the treating psychiatrist’s refusal or limited engagement for the patients themselves, for the psychotherapeutic relationship (e.g. which motives of (non-) referral may affect therapy compliance, inducing or resolving feelings of hopelessness), and for adequate euthanasia assessment.

## Supplementary information

**Additional file 1.**

## Data Availability

This study is fully disclosed, except for the database (for reasons of anonymity and privacy). To access the supplementary materials, see the Open Science Framework repository at: https://osf.io/asjvp/.

## Abbreviation

APC: Adults with Psychiatric Conditions
