# Supplementary material for: The engagement of psychiatrists in the assessment of euthanasia requests from psychiatric patients in Belgium: a survey study
Source: BMC Psychiatry. 2020 Aug 8;20:400. doi: 10.1186/s12888-020-02792-w (PMC7414658; doi:10.1186/s12888-020-02792-w)
Supplement: Supplementary file 1 — Additional file 1. [file 12888_2020_2792_MOESM1_ESM.zip › Appendix A_Survey_Psychiatric Euthanasia_General Part 1_in Dutch.pdf]

Algemeen deel: attitudes en ervaringen van (Assistent-)Psychiaters met Euthanasie

Luik 1: Enkele algemene professionele en persoonlijke vragen

1. Was u de voorbije 12 maanden werkzaam als (assistent-)psychiater van volwassen patiënten? ☐ Ja ☐ Nee

2. Was u in deze periode als psychiater werkzaam (meerdere antwoorden mogelijk):

- ☐ In een privé (groeps)praktijk
- ☐ In een ziekenhuis (PAAZ/PZ)
- ☐ In een Centrum voor Geestelijke Gezondheidszorg (CGG)
- ☐ In een Psychiatrisch Verzorgingstehuis (PVT)
- ☐ Psychiatrische Zorg Thuissituatie (PZT)
- ☐ Initiatief Beschut wonen
- ☐ Anders, nl.:………

3. Sinds hoeveel jaren bent u als psychiater werkzaam (geweest), inclusief als assistent?

☐ Minder dan 5 jaar ☐ 6 tot 10 jaar ☐ 11 tot 20 jaar ☐ Meer dan 20 jaar

4. Heeft u een gespecialiseerde opleiding gevolgd rond palliatieve en/of andere levenseindezorg? ☐ Ja ☐ Nee

5. Voelt u zich voldoende competent om als psychiater betrokken te worden bij een euthanasieprocedure? ☐ Ja ☐ Nee

6. Wat is uw leeftijd? ☐ Jonger dan 30 ☐ 30 - 40 jaar ☐ 41-60 jaar ☐ Ouder dan 60

7. Wat is uw geslacht? ☐ Man ☐ Vrouw ☐ X

Luik 2: Stellingen

8. *Geef aan in hoeverre u het eens of oneens bent met de volgende 13 stellingen. Het gaat hierbij telkens om uw persoonlijke mening, niet om wat al dan niet wettelijk toegestaan is. Er zijn dus geen juiste of foute antwoorden.* Noot: bij stellingen over psychiatrische patiënten, gaat het louter over patiënten met een psychiatrische ziektebeeld als onderliggende aandoening.

	Helemaal oneens				Helemaal eens
Euthanasie zou enkel voor terminaal zieke patiënten wettelijk mogelijk moeten zijn.	☐	☐	☐	☐	☐
Euthanasie zou voor niet-terminale patiënten wettelijk mogelijk moeten zijn, maar enkel als er sprake is van een somatische aandoening.	☐	☐	☐	☐	☐
Euthanasie zou voor patiënten met een psychiatrische aandoening wettelijk mogelijk moeten blijven.	☐	☐	☐	☐	☐
Euthanasie bij psychiatrische patiënten is verenigbaar met een psychiatrische hulpverleningsrelatie.	☐	☐	☐	☐	☐
Bij psychiatrische patiënten is medisch begeleide zelfdoding (patiënt neemt zelf de letale dosis in, in aanwezigheid en onder begeleiding van de arts) voor mij meer aanvaardbaar dan euthanasie (arts dient letale dosis toe aan de patiënt).	☐	☐	☐	☐	☐
Een psychiatrische patiënt kan zich in een medisch uitzichtloze toestand bevinden.	☐	☐	☐	☐	☐
Een psychiatrische patiënt kan ondraaglijk lijden.	☐	☐	☐	☐	☐
Een redelijk behandelperspectief kan ontbreken bij een psychiatrische patiënt.	☐	☐	☐	☐	☐
Euthanasie is een aanvaardbaar alternatief om suïcide te voorkomen.	☐	☐	☐	☐	☐
Bij de uitklaring van een euthanasieverzoek moet rekening gehouden worden met mogelijke effectieve therapeutische ontwikkelingen in de toekomst.	☐	☐	☐	☐	☐
Bij de uitklaring van een euthanasieverzoek dient de focus niet louter op de medische, maar op de volledige leefsituatie van de patiënt gelegd te worden.	☐	☐	☐	☐	☐
Een doodswens van een psychiatrische patiënt kan weloverwogen zijn en niet louter een symptoom van een onderliggende pathologie.	☐	☐	☐	☐	☐
In bepaalde gevallen wordt er te lichtzinnig omgegaan met de mogelijkheid van euthanasie voor psychiatrische patiënten.	☐	☐	☐	☐	☐

Luik 3: Onderstaande vragen polsen naar uw ervaringen met euthanasieverzoeken van VOLWASSEN patiënten die HOOFDZAKELIJK GEBASEERD waren op het lijden van de patiënt aan één of meerdere psychiatrische aandoening(en), anders dan dementie.

9. Heeft u **als behandelend arts** van een psychiatrische patiënt ooit geweigerd om actief betrokken te zijn bij de uitklaring van diens uitdrukkelijk euthanasieverzoek? ☐ Ja ☐ Neen

10. Om welke reden(en) heeft u hiertoe besloten?	……………………………………………………………………………………… ………………………………………………………………………………………

11. Werd u tijdens uw loopbaan als psychiater ooit betrokken bij een uitdrukkelijk euthanasieverzoek van een volwassen patiënt die hoofdzakelijk leed aan 1 of meer psychiatrische aandoening(en)? (Meerdere opties mogelijk)

☐ Nee, ik werd hier nooit mee geconfronteerd **(→ vraag 15)**
☐ Ja, als behandelend arts, die de eigen patiënt **doorverwees naar een collega-arts** i.f.v. een euthanasieprocedure
☐ Ja, als behandelend arts, die de uitklaring van het euthanasieverzoek van **een eigen patiënt** zelf opnam
☐ Ja, als arts die de uitklaring van het euthanasieverzoek van een **patiënt van een collega-arts** opnam
☐ Ja, als **preliminair adviserend arts** over een deelaspect (bv. uitsluiten acute depressie, beoordelen wilsbekwaamheid)
☐ Ja, als **procedureel adviserend arts** in het kader van een wettelijk vereist vereist 1ste of 2de euthanasie-advies
☐ Ja, als uitvoerend arts die euthanasie bij een **eigen patiënt** uitvoerde, hierbij assisteerde of aanwezig was
☐ Ja, als uitvoerend arts die euthanasie bij een **patiënt van een collega-arts** uitvoerde/assisteerde of erbij aanwezig was
☐ Ja, in een andere rol, nl……

12. Bij hoeveel euthanasieverzoeken van dergelijke patiënten werd u de afgelopen 12 maanden (in eender welke professionele rol) betrokken?

☐ 0 patiënten **(→ vraag 14)**	☐ 1-2 patiënten	☐ 3-5 patiënten	☐ 5-9 patiënten	☐ 10-20 patiënten	☐ > 20 patiënten

13. Hoe vaak heeft u de voorbije 12 maanden een positief dan wel negatief advies gegeven of een advies geweigerd?

Positief advies	☐ Niet van toepassing	☐ 1-2 patiënten	☐ 3-5 patiënten	☐ Meer dan 5 patiënten
Negatief advies	☐ Niet van toepassing	☐ 1-2 patiënten	☐ 3-5 patiënten	☐ Meer dan 5 patiënten
Advies geweigerd	☐ Niet van toepassing	☐ 1-2 patiënten	☐ 3-5 patiënten	☐ Meer dan 5 patiënten

14. Bij hoeveel psychiatrische patiënten was u de afgelopen 5 jaar als uitvoerend arts betrokken?

☐ 0 patiënten	☐ 1-2 patiënten	☐ 3-5 patiënten	☐ Meer dan 5 patiënten

15. Acht u het denkbaar dat u in de toekomst één of meer van volgende rollen opneemt in het kader van concrete euthanasieverzoeken van volwassen patiënten met (een) psychiatrische aandoening(en). (Meerdere opties mogelijk)

☐ Nee, in geen enkele rol
☐ Ja, als behandelend arts, die de eigen patiënt doorverwijst naar een collega-arts voor de verdere uitklaring/advies
☐ Ja, als behandelend arts, die de uitklaring van het euthanasieverzoek van een **eigen patiënt** zelf opneemt
☐ Ja, als arts die de uitklaring van het euthanasieverzoek van een **patiënt van een collega-arts** opneemt
☐ Ja, als **preliminair adviserend arts** over een deelaspect (bv. uitsluiten acute depressie, beoordelen wilsbekwaamheid)
☐ Ja, als **procedureel adviserend arts** in het kader van een wettelijk vereist 1ste of 2de euthanasie-advies
☐ Ja, als uitvoerend arts, die de euthanasie bij een **eigen patiënt** uitvoert, hierbij assisteert of aanwezig is
☐ Ja, als uitvoerend arts, die de euthanasie bij een **patiënt van een collega-arts** uitvoert, hierbij assisteert of aanwezig is
☐ Ja, in een andere rol, nl……

16. Heeft u ooit een extern consultatieteam, gespecialiseerd in de uitklaring van euthanasieverzoeken (bv. ULteam, Vonkel) betrokken bij de euthanasieprocedure van een psychiatrische patiënt?

☐ Nee, en ik zou dat ook nooit doen	☐ Ja, namelijk ………………………………………………………
☐ Nee, maar het is wel denkbaar dat ik dat ooit doe	………………………………………………………………………

17. Om welke reden(en) zou u al dan niet beroep op een extern consultatieteam doen?	……………………………………………………………………… ………………………………………………………………………

Wil u nog iets verduidelijken of toelichten?

……
……
……
……
……
……
……
……
