# Supplementary material for: The engagement of psychiatrists in the assessment of euthanasia requests from psychiatric patients in Belgium: a survey study
Source: BMC Psychiatry. 2020 Aug 8;20:400. doi: 10.1186/s12888-020-02792-w (PMC7414658; doi:10.1186/s12888-020-02792-w)
Supplement: Supplementary file 1 — Additional file 1. [file 12888_2020_2792_MOESM1_ESM.zip › Appendix B_Survey_PsychiatricEuthanasia_Optional Part 2_in Dutch.pdf]

Facultatief deel: uw laatste ervaring met euthanasie

Volgende vragen zoomen in op uw laatste ervaring met een volledig afgeronde (ongeacht de einduitkomst) euthanasieprocedure van een patiënt met een psychiatrische aandoening tijdens de voorbije 12 maanden.

1. Wat was uw specifieke functie? (meerdere opties mogelijk)

☐ Behandelaar van de patiënt en diens psychopathologie
☐ Uitklaring euthanasieverzoek van een eigen patiënt
☐ Uitklaring euthanasieverzoek van een patiënt van een collega-arts
☐ Preliminair adviserend arts
☐ Procedureel adviserend arts
☐ Uitvoerend arts

2. Hoeveel tijd nam de besluitvorming in beslag vanaf het euthanasieverzoek tot de eindbeslissing? (vul aantal weken/maanden/jaren in) ....

3. Hoe lang was de patiënt reeds in behandeling voor hij/zij u consulteerde met een euthanasieverzoek? (Specifieer aantal weken/maanden/jaren) ....

4. Volgde de patiënt psychotherapeutische behandeling(en) op het moment dat hij/zij u voor het eerst consulteerde in het kader van euthanasie? (meerdere opties mogelijk)

☐ Nee, patiënt volgde geen behandeling
☐ Ja, psychofarmaca ☐ Ja, andere farmaca ☐ Ja, gesprekstherapie ☐ Ja, neurochirurgische behandeling
☐ Andere interventies, nl.:..
....

5. Wat was de belangrijkste pathologie van de patiënt? (meerdere aankruis- en invulopties mogelijk)

☐ Klinische stoornis, nl..
☐ Persoonlijkheidsstoornis, nl...
☐ Somatische aandoeningen, nl...

6. Wat waren de redenen van de patiënt om euthanasie te vragen? (meerdere antwoorden mogelijk)

☐ Depressieve gevoelens
☐ Vastlopen op meerdere levensgebieden (werk/relatie/…)
☐ Geen perspectief op verbetering
☐ Geen doel (meer) in het leven
☐ Eenzaamheid
☐ Omgeving niet (langer) tot last willen zijn
☐ Existentieel lijden (lijden aan het leven, zinloosheid)
☐ Gestage deterioratie
☐ Angst voor suïcide
☐ Invaliditeit/immobiliteit
☐ Verlies van autonomie, regie over het eigen leven
☐ Verlies van waardigheid
☐ Totale uitputting
☐ Geen levenskwaliteit, enkel 'overleven'
☐ Andere, nl.:...
....

7. Wat waren volgens u de 2 voornaamste redenen van de patiënt om euthanasie te vragen?

....
....

8. Heeft u één of meerdere artsen/hulpverleners/instanties geconsulteerd tijdens de besluitvorming? (meerdere opties mogelijk)

☐ Neen
☐ Ja, de huisarts(en) van de patiënt
☐ Ja, andere arts(en) van de patiënt
☐ Ja, onafhankelijke collega-psychiater(s)
☐ Ja, onafhankelijke LEIF-arts(en)
☐ Ja, onafhankelijke arts(en) van levenseinde consultatieteam
☐ Ja, verpleegkundige(n)
☐ Ja, ethische commissie
☐ Ja, andere interne adviescommissie
☐ Ja, psycholo(o)gen
☐ Ja, palliatief zorgteam
☐ Ja, een (psycho-)sociale dienst
☐ Ja, andere(n), nl.:...

9. Heeft u overleg gehad met familie en/of vrienden van de patiënt? (meerdere antwoorden mogelijk)

☐ Neen, geen familie of vrienden betrokken
☐ Neen, patiënt had geen familie of vrienden
☐ Ja, tijdens euthanasieprocedure
☐ Ja, na afronding van de euthanasieprocedure

10. Was er bij deze patiënt naar uw oordeel sprake van…

Mentale competentie (wilsbekwaamheid)	☐ Ja	☐ Nee
Ongeneeslijkheid van de aandoening	☐ Ja	☐ Nee
Ondraaglijkheid van het lijden	☐ Ja	☐ Nee
Uitzichtloosheid van de medische conditie	☐ Ja	☐ Nee
Ontbreken redelijk therapeutisch perspectief	☐ Ja	☐ Nee
Vrijwillig, duurzaam en weloverwogen verzoek	☐ Ja	☐ Nee

11. Hoeveel moeilijkheden heeft u ervaren om onderstaande criteria te kunnen beoordelen?

	Geen				Erg veel
Mentale competentie (wilsbekwaamheid)	☐	☐	☐	☐	☐
Ongeneeslijkheid van de aandoening	☐	☐	☐	☐	☐
Ondraaglijkheid van het lijden	☐	☐	☐	☐	☐
Uitzichtloosheid van de medische conditie	☐	☐	☐	☐	☐
Ontbreken redelijk therapeutisch perspectief	☐	☐	☐	☐	☐
Vrijwillig, duurzaam en weloverwogen verzoek	☐	☐	☐	☐	☐
Andere, nl…………………………………………………………………………………………	☐	☐	☐	☐	☐

12. Werd u tijdens deze euthanasieprocedure geconfronteerd met onderstaande vormen van druk?

Patiënt die onder druk van derden om euthanasie verzoekt	☐ Ja	☐ Nee
Druk van patiënt om tot euthanasie te besluiten (advies/uitvoering)	☐ Ja	☐ Nee
Druk van naasten om tot euthanasie te besluiten (advies/uitvoering)	☐ Ja	☐ Nee
Druk van naasten om het euthanasieverzoek af te wijzen	☐ Ja	☐ Nee
Druk van collega's om het euthanasieverzoek af te wijzen	☐ Ja	☐ Nee
Druk van collega's om tot euthanasie te besluiten (advies/uitvoering)	☐ Ja	☐ Nee
Druk van zorginstelling om het euthanasieverzoek af te wijzen	☐ Ja	☐ Nee
Druk van zorginstelling om tot euthanasie te besluiten (advies/uitvoering)	☐ Ja	☐ Nee

13. Werd u tijdens deze euthanasieprocedure geconfronteerd met onderstaande ervaringen?

Hoge emotionele belasting voor uzelf	☐ Ja	☐ Nee
Nieuwe therapeutische kansen bij de patiënt	☐ Ja	☐ Nee
Verlaagd risico op suïcide bij de patiënt	☐ Ja	☐ Nee
Herstel relaties tussen patiënt en diens naasten	☐ Ja	☐ Nee
Mede-patiënten die ook om euthanasie verzochten	☐ Ja	☐ Nee

14. Wat was de aard van het (de) uiteindelijk verstrekte advies (adviezen) in functie van het euthanasieverzoek? (Meerdere antwoorden mogelijk)

☐ Er werden uiteindelijk geen adviezen verstrekt ☐ … positieve adviezen ☐ … negatieve adviezen

☐ Hierover werd ik niet geïnformeerd

15. Is de patiënt overleden via euthanasie?

☐ Weet ik niet ➔ vraag 17

☐ Ja ➔ vraag 16

☐ Nee, de euthanasieprocedure is nog niet afgerond ➔ vraag 17

☐ Nee, de patiënt trok het euthanasieverzoek *zonder druk van derden* terug in ➔ vraag 17

☐ Nee, de patiënt trok het euthanasieverzoek *onder druk van derden* terug in ➔ vraag 17

☐ Nee, de patiënt is op een andere manier overleden ➔ vraag 17

☐ Nee, want……➔ vraag 17

16. Was u aanwezig tijdens de euthanasie?

☐ Ja, en ik heb het letaal middel zelf toegediend (ev. geassisteerd door een ervaren collega)

☐ Ja, en ik heb het middel voorbereid, waarna een andere zorgverlener het aan de patiënt toediende

☐ Ja, en ik heb het middel voorbereid, waarna de patiënt het zelf innam

☐ Ja, maar ik heb het middel niet voorbereid en ook niet toegediend

☐ Nee

17. Heeft u voor uzelf emotionele ondersteuning gezocht tijdens of na de euthanasieprocedure? (Meerdere opties mogelijk)

☐ Nee ☐ Ja, in privékring ☐ Ja, bij collega's ☐ Ja, bij externe professionele hulpverlening ☐ Ja, bij anderen

18. Heeft deze casus uw houding ten aanzien van toekomstige verzoeken beïnvloed? ☐ Ja ☐ Neen➔ vraag 20

19. Op welke manier heeft het uw houding veranderd? ……………………………………………………………………………

……………………………………………………………………………

20. Wil u nog iets verduidelijken of toelichten over uw ervaring/gevoelens m.b.t. deze casus?

………

………
