# Supplementary material for: The engagement of psychiatrists in the assessment of euthanasia requests from psychiatric patients in Belgium: a survey study
Source: BMC Psychiatry. 2020 Aug 8;20:400. doi: 10.1186/s12888-020-02792-w (PMC7414658; doi:10.1186/s12888-020-02792-w)
Supplement: Supplementary file 1 — Additional file 1. [file 12888_2020_2792_MOESM1_ESM.zip › Appendix C_Research Protocol_SurveyStudy_in English(1).docx]

**Flemish (Assistant-)Psychiatrist’s Attitudes and Experiences with Psychiatric Euthanasia Requests and Procedures**

Work Package to the FWO awarded research project (G017818N)

and PhD fellowship (1162618N)

Authors:

Monica Verhofstadt, doctoral researcher (FWO aspirant 1162618N), clinical psychologist
Prof. dr. Kenneth Chambaere, promoter (Vrije Universiteit Brussel (VUB) – Ghent University), medical sociologist
Prof. dr. Kurt Audenaert, copromoter (Ghent University), psychiatrist (University Hospital Ghent), psychiatrist

**Table of Content**

[Background 3](#_Toc519698112)

[Research Aims 4](#_Toc519698113)

[Methods 5](#_Toc519698114)

[Design 5](#_Toc519698115)

[Participants 5](#_Toc519698116)

[Critical Reflection 6](#_Toc519698117)

[Procedure 6](#_Toc519698118)

[Procedure to maximise response rate with respect to confidentiality 7](#_Toc519698119)

[Measurement Instrument 9](#_Toc519698120)

[Data analysis 11](#_Toc519698121)

[Timing 12](#_Toc519698122)

[Ethical Considerations 12](#_Toc519698123)

[The research team 13](#_Toc519698124)

[Members of the Flemish Association of Psychiatry 13](#_Toc519698125)

[Extended collaboration (Advisory Board) 13](#_Toc519698126)

[References 15](#_Toc519698127)

# Background

In Belgium, euthanasia can be justifiable in both terminally ill and non-terminally ill patients, with stricter legal requirements imposed upon the latter patient group. In case of euthanasia requests from the non-terminally ill to be justifiable, the advice of not one, but at least two physicians, among them at least one expert in patient’s disorder, during the decision making procedure is mandatory in Belgium, though not binding.^1,2^ Furthermore, a one-month waiting period between the written euthanasia request and euthanasia performance is mandatory.^1,2^ Hence, in the case of the psychiatric patients with a euthanasia request, the advice of at least one psychiatrist is legally required in evaluating all legal and due care criteria. According to the latest prevalence rates of all performed euthanasia cases in Belgium, 3.1% concerned patients with psychiatric disorders or dementia.^3–5^ Although representing a minority of carried out euthanasia cases, psychiatric euthanasia cases remain subject of great debate and concerns, e.g. due to scientific research revealing strong dissension between physicians on whether or not all legal criteria are consistently and adequately assessed and thus, are met.^6,7^ For example, in more than half of the performed Dutch euthanasia cases, patient’s mental capacity was not evaluated in a systematic way.^7^

The importance of psychiatrists’ expertise and role as additional gatekeeper^8–10^ is confirmed in the Belgian clinical euthanasia practice, as in all performed psychiatric euthanasia cases at least one psychiatrist was involved in euthanasia assessment and evaluation procedures - with the exception of one case which was transferred to the Belgian prosecutor.^2,11^ Moreover, the specific role of psychiatrists is not always restricted to euthanasia requests based on mental suffering from psychiatric disorders as psychiatrists are often involved in euthanasia cases based on suffering from somatic disorders as well. In the years 2014 and 2015, 124 performed euthanasia cases were based on psychiatric and behavioural illnesses, psychiatrists were consulted in over three times as many performed euthanasia cases (406 to be precise) for advice and evaluation of all medical and legal criteria.^3^

However, to date, no research has focussed on the attitudes and experiences of Belgian psychiatrists with the psychiatric euthanasia practice as a whole. This is striking, as recent trend-analysis in Belgium has shown an increase in carried out psychiatric euthanasia cases over time^2^ and it remains subject of fierce (media) debates between proponents and opponents given a wide range of fundamental ethical, societal and legal concerns.^12,13^ For example, there is still some concern regarding potentially overly permissive approaches in managing euthanasia requests from the mentally ill.^14^ Moreover, nothing is known about how many euthanasia requests are – for which reasons – granted, refused, denied or even withdrawn by the patient him- or herself. To date, only one Belgian study revealed limited descriptive information on the psychiatric euthanasia practice, based on a small sample of 100 patients from a private clinical practice.^15^ In total, 48 of the 100 euthanasia requests were accepted, and 35 carried out. Six patients committed suicide (two of them during the euthanasia preparatory procedures) and 11 patients postponed or cancelled the euthanasia. Among them, 8 patients explicitly declared that having the option to die by euthanasia, gave them enough peace of mind to continue living.^15^ However, no data were revealed on how many psychiatrists were involved as treating, advising or performing physician and to what extent they were involved in the decision making procedures regarding the evaluation of essential legal criteria. Furthermore, nothing is known about the attitudes and experiences of psychiatrist assistants, to some extent tutored and to another extent having their own stances on euthanasia that could influence the future of psychiatry. A deeper insight in the role, involvement and conceivability of (assistant-)psychiatrists regarding the management of (former, actual and future) psychiatric euthanasia requests is quintessential to provide the debate with sound arguments.

As of now, only the Netherlands regularly provided in evaluation studies on the euthanasia practice. Belgium also needs to undertake these most needed scientific endeavours in order to provide not only the Belgian, but also the international society with sufficient detail, especially when it concerns the most vulnerable population of psychiatric patients. Hence, this research project can thus be seen as an effort to set the research agenda in order to provide deeper insights in today’s psychiatric euthanasia practice.

Research Aims

This study aims to gain insight in the attitudes of (assistant-)psychiatrists regarding euthanasia requests and practice as well as in the prevalence and experiences of (assistant-)psychiatrists being confronted and involved with psychiatric euthanasia requests and procedures.

More specifically this study aims to address the following research questions:

1. What are **(assistant-)psychiatrists’ attitudes** towards euthanasia practice in general and in psychiatric patients specifically?
2. To what extent can they conceive of themselves refusing, granting and/or performing euthanasia for psychiatric patients?
3. What are **(assistant-)psychiatrists’ experiences** with the euthanasia practice?

During the last year and throughout their career, how often have (assistant-)psychiatrists been confronted with euthanasia requests from psychiatric patients? And how often, to what extent and in what capacity have they been involved in the management of psychiatric euthanasia procedures?

1. **How do (assistant-)psychiatrists handle requests for euthanasia from psychiatric patients?** Which other important actors (family, friends and other caregivers) are or can be involved during the euthanasia decision making procedures? Are there specific needs to be distinguished that could help (assistant-)psychiatrists adequately address the complexity of euthanasia requests and procedures?
2. **To what extent are (assistant-)psychiatrists’** **attitudes, conceivability, experience and handling of euthanasia requests for psychiatric patients related to their** socio-demographic and professional background**?**

This research objective is a Work Package to the FWO awarded PhD fellowship of Monica Verhofstadt (1162618N) and FWO research project (G017818N).

# Methods

## Design

This cross-sectional study consists of a paper and web survey, gathering data on (assistant-)psychiatrists’ attitudes and experiences regarding euthanasia requests and procedures in psychiatric patients.

## Participants

The sample will consist of (assistant-)psychiatrists, associated to the Flemish Psychiatry Association (VVP) (estimated *N* = 700, of which 85 assistant-psychiatrists). No further exclusion criteria will be employed.

Procedure

Data collection
 The associated members of the VVP will be approached via a 5-step mailing procedure, according to the principles of Dillman’s ‘Tailored Design Survey Method’ to maximise the response rate.^16^
Step 1 (day 0): First, potential respondents will be contacted by an e-mail from the VVP president as sender, with the invitation to participate in this study. The mail will contain an information letter (see appendix A), in which the purpose of the study, relevant ethical considerations such as risks, anonymity, data protection and confidentiality, and contact addresses of the research team will be presented to the respondents. The mail will also contain a link to the online questionnaire in LimeSurvey,^17^ which will be made accessible also through the website euthanasiaresearch.eu and the VVP home page <http://www.vvp-online.be/>. At the start of the survey, a brief, but concise informed consent statement will be given in which the purpose, timing, and relevance regarding, and some key terminology used in this study, will be presented.

Step 2 (day 14): A reminder will be sent to all potential psychiatrists by the VVP president via e-mail. Again, the potential participant will be first given an informative introduction letter (see appendix A) in which the purpose, timing, and relevance regarding, and some key terminology used in this study, will be presented. The mail will also include the message that a paper-and-pencil version will be sent in a prepaid (with return address printed) envelope to all psychiatrists in case the postal survey would be preferred over the online survey.

Step 3 (day 21): The paper-and-pen version will be sent by post via the VVP. The potential participant will be first given an informative introduction letter (see appendix B) in which the purpose, timing, and relevance regarding, and some key terminology used in this study, will be presented. The introduction letter will also mention the possibility to participate online, with link to the online survey. Questionnaire 1 and 2 will be included, as well as a prepaid envelope with MV’s office address as return address.

~~Step 4 (day 35): A reminder will be sent to non-responding psychiatrists via e-mail.
Step 5 (day 50): A final reminder will be sent to non-responding psychiatrists via post, but this time with a reduced version of the survey instrument (only questionnaire 1, see the section ‘Survey Instrument’).~~

In all cases, data will be collected as soon as ethical approval has been obtained, thus approximately during the months of October 2018 and January 2019.

##### Procedure to maximise response rate with respect to confidentiality

A data manager will be engaged to coordinate the data collection, follow up procedures, and facilitate data entry. By doing so, the role of the executive researcher will be restricted to answering all potential answers from the (potential) participants regarding this survey study and performing data analysis.

Each potential participant will be given a unique personal token code. The unique token code (automatically generated) will be linked to the VVP members in the survey database together with their contact details. This token code is only meant to guide the follow-up/reminder process.

By using unique token codes and macro functionality in Excel, the medical secretary will be able to send the websurvey link to all VVP-members (step 1). Participating VVP members have to use their unique token code to access the websurvey. Response will be tracked by the data manager. He will report the token codes for which response was received to the medical secretary of the VVP, who will then delete all VVP members in the survey database corresponding to these token codes (i.e. responding). With this procedure, reminders will only be sent to VVP members who have not completed the survey (step 2-5). After data collection is concluded, the survey database will be deleted altogether.

Collected online data will be automatically stored in the response database (excel-file). Collected data from the postal survey will be added manually by the executive researcher in the same database/Excel-file after step 5. In order to further guarantee participants’ anonymity, the response database will be shared only with MV, KC and KA via SYNC (encrypted) on their secured servers. See Appendix C for a signed Agreement Letter from the VVP-president.

##### Ethical consideration regarding GDPR-compliance for online data collection

In the online survey tool, explicit consent from the respondent will be asked by inserting the question “Do you agree to take part in this survey?” immediately after the informed consent statement and right before the start of the survey. As explicit consent has to be given affirmatively, a radio button will be created in LimeSurvey.

- If respondents click the option ‘Yes’, they can continue to take part in the survey and submit their data online. All respondents will also be given the option to withdraw their data at any point (via the easy to opt-out ‘skip logic’ system).

If respondents click the option ‘No’, they will be sent directly to the ‘Non-response Questionnaire’ (see box 1).

| BOX 1 Answer options of the non-response questionnaire (multiple answers possible)  Bad timing, the survey does not fit in my current agenda  I’m not interested in the topic  The survey interferes with my privacy  I never participate in surveys  I do not trust surveys in general  Other: (open question) |
| --- |

According to the GDPR guidelines of May 2018, an internal register will provide all required information on e.g. the purposes of all processing operations; a description of the categories of data subjects and the recipients or categories of recipients to whom the data are disclosed; the legal basis of the processing operation for which the data are intended (see Appendix D for a detailed internal register of all procession operations). This internal register has been sent to the data protection officer (DPO) of the VUB on August, 28th.

Measurement Instrument
Survey instrument development
 The survey instrument is based on 5 existing questionnaires: two Dutch and three Flemish surveys, aiming to investigate physicians’ attitudes and experiences regarding the euthanasia practice for research and legal evaluation purposes^18–22^ and adjusted to the context of psychiatric clinical practice in Flanders. Many items were deleted or reformulated in consultation with three experienced psychiatrists of the Flemish Psychiatry Association (VVP) member board. Additional questions were added according to the in detail formulated research questions. Subsequently, the survey draft had been presented to the members of the project group and advisory board for extended feedback. In order to develop user-friendly questionnaires, asking a minimum of time and effort from (assistant-)psychiatrists, the survey was divided in two parts: one general part to be completed by every psychiatrist and psychiatrist assistant, irrespective of them being involved in concrete euthanasia cases, and a facultative part focussing on the last concrete involvement in a euthanasia case. Most items consist of check-box answers, with a minimum number of items containing open answer categories. On the other hand, one optional comment box has been added both at the end of the general and facultative part of the survey, so respondents qualify or clarify one or more of their responses.

Then, the survey draft was presented at a meeting with 15 (assistant-)psychiatrists of the psychiatry ward of the Ghent University Hospital (UZ Ghent) for a cognitive validation in order to capture the maximum time spent on the survey and to gain additional unprejudiced feedback on acceptability regarding length, form and content, and exclusion of social desirability. The survey was adjusted according to the feedback of these 15 (assistant-)psychiatrists. Finally, the adjusted survey was presented to the three VVP-psychiatrists, the members of the project group and advisory board for final feedback and approval.

The online version of the survey will be created in LimeSurvey ^17^ and checked for unexpected technical problems and time estimation by a small sample of ten researchers from the End-of-Life Care research group.

##### Survey instrument

The survey consists of two separate but related parts that together examine (assistant-)psychiatrists’ attitudes, conceivability and experiences towards psychiatric euthanasia cases.

**The general part**: contains 29 items and is divided in three main parts:

1) Seven general items; three items on professional specialism and work environment, two items on education and competence regarding the clinical end-of-life practice, one item on gender and one item on age category,

2) 13 statements on attitudes towards euthanasia).
3) Nine items on experiences and conceivability towards psychiatric euthanasia requests and procedures in terms of the extent of being confronted or involved with euthanasia requests as e.g. treating, advising and/or performing psychiatrist throughout respondents’ career or/ and in the future.

At the end of the sub-questionnaire an optional comment box is included in case respondents want to elucidate some of the given answers.

**The facultative part** is restricted for (assistant-)psychiatrists who have managed at least one euthanasia request in their professional career and contains 19 items on their last fully completed psychiatric euthanasia procedure. The facultative part also contains an optional comment box for respondents to elucidate some of the given answers.
 Depending on the experience with euthanasia requests and procedures, respondents will be asked to answer minimum 27 items, including 13 statements, when not experienced, or maximum 48 items when experienced at a great extent in the psychiatric euthanasia practice (See Appendix E and F for respectively the general and facultative part of the survey).

Prevention and handling of missing answers
 Measures to reduce the possibility of missing answers to a bare minimum were put in place according to recent methodological standards^23,24^. As mentioned above, the survey was developed in consultation with three experienced psychiatrists and presented in a meeting with 15 (assistant-)psychiatrists in order to test for acceptability both in length, form and content (gathering of only quintessential information). The survey was then imported in Limesurvey, using the server setting enabling to explicitly refuse to answer some items as well as to leave the online session while automatically saving the already registered answers and return to the survey on a later time.

Data analysis

Only data from Dutch-speaking respondents who were recently working as (assistant-)psychiatrist of adult patients will be included for analysis purposes. This means that the data from (assistant-)psychiatrist responding ‘No’ at the first question “During the past 12 months, were you professionally active as (assistant-) psychiatrist for adult patients?” will be a posteriori excluded. Descriptive statistics will be reported on the following characteristics of the participants: gender, age category, working environment, and being educated in handling euthanasia request (frequency, percentage, mode or median). As for years of practice and whether or not (and if so, at what extent) being confronted with euthanasia requests, descriptive statistics will also be reported (with frequency, percentages, means, and standard deviation).

In case of missing data, the number of missing answers will be transparently reported, included for analysis via multiple imputation methods and commented in the discussion section of scientific reports.

Furthermore, multivariate analysis will be carried out to examine whether experiences, conceivability, and attitudes towards euthanasia correlated with gender, age category, years and type of work experience, and stepwise backward multiple regression (removal at *p* < 0.01) will be performed. All results will be reported using 95% confidence intervals. Statistical analysis will be carried out using SPSS Statistics software.

Timing

The survey will be conducted, pending ethics approval, in the period September-November 2018.

Ethical Considerations
 Ethical approval from the Medical Ethics Committee of UZ Brussels (as central institution) and the Medical Ethics Committee of UZ Ghent (as local institution) will be sought.

As for respondents’ anonymity and privacy, no directly identifiable information will be gathered. Respondents’ personal and professional characteristics (e.g. gender, years of professional activity) will only be used by means of aggregated descriptive statistics (in order to describe the sample) and as independent variables in statistical analyses.

Regarding online data protection and safety, privacy policies of online survey software and cloud storage services have been checked already. The data protection statement of LimeSurvey (<https://www.limesurvey.org/data-protection-statement>) implies safeguards in online data protection and security (e.g. against inadvertent or deliberate manipulation and unauthorized persons). LimeSurvey settings, specifically used to collect response-related details (e.g. the referrer URL, IP address, timings, etc.) will not be used nor tracked via Google Analytics as they are irrelevant to the purposes of this study.

One day after the survey expiry date, online data will be imported into a SPSS-file, only shared between KA, KC and MV in a secured encrypted Sync folder^25^ on their secured servers. After data is imported in the Sync folder, the LimeSurvey will be deactivated and data erased from the online platform.

Although data property rights are owned by the Vrije Universiteit Brussel (VUB) and Ghent University, KA, KC and MV will have intellectual rights on data storage and use, only to the extent necessary for the abovementioned scientific research purposes, until the study has been submitted and approved for publication in a scientific journal. However, as for transparency and reproducibility (i.e. good scientific practice) data will be accessible following procedures from the Ethics Committee of UZ Brussels and UZ Ghent.
Collaboration

The research team
This research project features as Work Package of the research project entitled “Euthanasia requests from psychiatric patients in Flanders: motives, experiences, management and outcomes”. The FWO awarded a research project (G017818N) and PhD fellowship (1162618N) was assigned to the research team:

| - Prof. Kenneth Chambaere, promotor and senior researcher End of Life Research Group (VUB-UGhent) - Prof. dr. Kurt Audenaert, copromotor, psychiatrist and senior researcher (UGhent-UZGhent) - MSc Monica Verhofstadt, doctoral researcher End of Life Research Group (VUB-UGhent)  The research team further includes: - Prof. Dr. Luc Deliens (medical sociologist), director of the End of Life Care Research Group |
| --- |

Members of the Flemish Association of Psychiatry
The survey was developed in close collaboration with the following psychiatrists and members of the VVP:

- Dr. Kris van den Broeck (affiliated to Antwerp University)
- Dr. Koen Titeca (affiliated to AZ Groeninge, Kortrijk)
- Prof. dr. Joris Vandenberghe (affiliated to Catholic University of Leuven and UZ Leuven)

## Extended collaboration (Advisory Board)

During the course of the entire FWO awarded research project and thus also during this specific research project, the research team will consult the following advisory board of experienced, independent researchers for supervision, multidisciplinary perspectives and ethical integrity:

- Prof. dr. Dirk De Bacquer, medical statistician and epidemiologist, Ghent University
- Prof. dr. Piet Bracke, sociologist, Ghent University
- Prof. dr. Bregje Onwuteaka-Philipsen, health scientist, Vrije Universiteit Amsterdam
- Prof. Dr. Freddy Mortier, ethicist, Ghent University
- Dr. Koen Titeca, psychiatrist, AZ Groeninge Kortrijk

Date and Signature lead researcher and promotor, prof.dr. Kenneth Chambaere


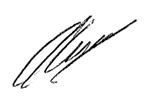
27 August, 2018

Date and Signature Copromotor promotor, prof. dr. Kurt Audenaert

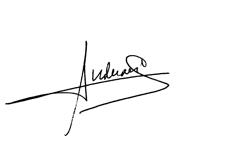
27 August, 2018

Date and Signature of the director of the End of Life Care Research Group, prof. dr. Luc Deliens:


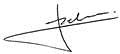
27 August, 2018

Date and Signature Executive Researcher, Monica Verhofstadt


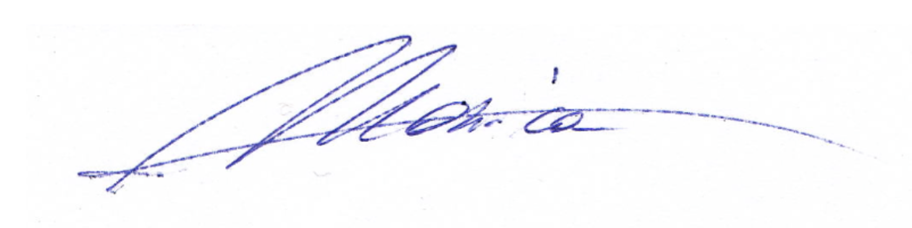
27 August, 2018

# References

1. *WET WE*. Brussels, Belgium; 2002. http://www.npzl.be/files/107a_B3_Wet_euthanasie.pdf.

2. Dierickx S, Deliens L, Cohen J, Chambaere K. Euthanasia for people with psychiatric disorders or dementia in Belgium: analysis of officially reported cases. *BMC Psychiatry*. 2017;17(1):203. doi:10.1186/s12888-017-1369-0.

3. FCEC. *Seventh Report to the Parliament (2014–2015)*. Brussels, Belgium; 2016.

4. KNMG. *Infographics Euthanasia May 2016*. Utrecht, The Netherlands; 2016.

5. KNMG. *Euthanasie in Cijfers_april 2017*. Utrecht, The Netherlands; 2017. https://www.knmg.nl/advies-richtlijnen/dossiers/euthanasie.htm.

6. Kim SYH, De Vries RG, Peteet JR. Euthanasia and assisted suicide of patients with psychiatric disorders in the Netherlands 2011 to 2014. *JAMA Psychiatry*. 2016;73(4):362-368. doi:10.1001/jamapsychiatry.2015.2887.

7. Doernberg SN, Peteet JR, Kim SYH. Capacity Evaluations of Psychiatric Patients Requesting Assisted Death in the Netherlands. *Psychosomatics*. 2016;57(6):556-565. doi:10.1016/j.psym.2016.06.005.

8. Naudts K, Ducatelle C, Kovacs J, Laurens K, Van Den Eynde F, Van Heeringen C. Euthanasia: The role of the psychiatrist. *Br J Psychiatry*. 2006;188(MAY):405-409. doi:10.1192/bjp.bp.105.010256.

9. Roy K. Role of Psychiatrists in Assisted Dying : A Changing Trend. 2015:5-7.

10. Olie E, Courtet P. The Controversial Issue of Euthanasia in Patients With Psychiatric Illness. *JAMA-JOURNAL Am Med Assoc*. 2016;316(6):656-657. doi:10.1001/jamapsychiatry.2015.2887.

11. Symons X. Belgian euthanasia doctor faces criminal charges. *Bioethics news from around the world*. https://www.bioedge.org/bioethics/belgian-euthanasia-doctor-faces-criminal-charges/11635. Published October 25, 2015.

12. Bazan A, Van de Vijver G, Lemmens W. “Remove Euthanasia on the Basis of Purely Psychological Suffering from the Legislation” (Translation Open Letter). Lemmens Trudo (Translation.

13. Braeckman J, Ravelingien A, Boudry M. “Don’t Trivialize Psychological Suffering” (translation response letter). Lemmens, Trudo (Translation). https://trudolemmens.wordpress.com/2015/12/14/dont-trivialize-psychological-suffering/. Published 2015. Accessed December 14, 2017.

14. Clifford JM. Where is the argument for the conceptual Slippery Slope? Response letter to Verhofstadt M., Thienpont L., & Peters GJY. (2017) “When unbearable suffering incites psychiatric patients to request euthanasia: qualitative study.” 2017;249(1):2017. doi:10.1192/bjp.211.6.397.

15. Thienpont L, Verhofstadt M, Van Loon T, Distelmans W, Audenaert K, De Deyn PP. Euthanasia requests, procedures and outcomes for 100 Belgian patients suffering from psychiatric disorders: a retrospective, descriptive study. *BMJ Open*. 2015;5(7):e007454. doi:10.1136/bmjopen-2014-007454.

16. Dillman DA. Mail and internet surveys: The tailored design method. *J Contin Educ Health Prof*. 2007;2(3):207. doi:10.1017/CBO9781107415324.004.

17. LimeSurvey Project Team / Carsten Schmitz. *LimeSurvey: An Open Source Survey Tool*. Hamburg, Germany; 2017.

18. Onwuteaka-Philipsen BD, Legemaate J, van der Heide A, et al. *Derde Evaluatie Wet Toetsing Levensbeëindiging Op Verzoek En Hulp Bij Zelfdoding*.; 2017.

19. Cohen J, Van Wesemael Y, Smets T, et al. Nationwide survey to evaluate the decision-making process in euthanasia requests in Belgium: do specifically trained 2nd physicians improve quality of consultation? *BMC Health Serv Res*. 2014;14(1):307. doi:10.1186/1472-6963-14-307.

20. Smets T, Cohen J, Bilsen J, Van Wesemael Y, Rurup ML, Deliens L. Attitudes and experiences of belgian physicians regarding euthanasia practice and the euthanasia law. *J Pain Symptom Manage*. 2011;41(3):580-593. doi:10.1016/j.jpainsymman.2010.05.015.

21. Hanssen-de Wolf JE, Pasman HRW, Onwuteaka-Philipsen BD. How do general practitioners assess the criteria for due care for euthanasia in concrete cases? *Health Policy (New York)*. 2008;87(3):316-325. doi:10.1016/j.healthpol.2007.12.009.

22. Bolt EE, Snijdewind MC, Willems DL, van der Heide A, Onwuteaka-Philipsen BD. Can physicians conceive of performing euthanasia in case of psychiatric disease, dementia or being tired of living? *J Med Ethics*. 2015;41(8):592-598. doi:10.1136/medethics-2014-102150.

23. Li T, Hutfless S, Scharfstein DO, et al. Standards should be applied in the prevention and handling of missing data for patient-centered outcomes research: A systematic review and expert consensus. *J Clin Epidemiol*. 2014;67(1):15-32. doi:10.1016/j.jclinepi.2013.08.013.

24. Kang H. The prevention and handling of the missing data. *Korean J Anesthesiol*. 2013;64(5):402-406. doi:10.4097/kjae.2013.64.5.402.

25. Sync.com Inc. Secure Cloud Storage. 2017. https://www.sync.com/.
