# Supplementary material for: The engagement of psychiatrists in the assessment of euthanasia requests from psychiatric patients in Belgium: a survey study
Source: BMC Psychiatry. 2020 Aug 8;20:400. doi: 10.1186/s12888-020-02792-w (PMC7414658; doi:10.1186/s12888-020-02792-w)
Supplement: Supplementary file 1 — Additional file 1. [file 12888_2020_2792_MOESM1_ESM.zip › Appendix D_Items_PsychiatristsConcreteExperiences_Psy_English.pdf]

Psychiatrists' Concrete Experiences with Psychiatric Euthanasia Requests and Procedures

Part 1: Some professional and personal questions in general

1. During the past 12 months, have you worked as psychiatrist (or trainee) with adult patients? ☐ Yes ☐ No

2. Have you worked (more answer options plausible):

☐ In a private or Group Practice
☐ In a Psychiatric Hospital Care
☐ In a Community Mental HealthCare Centre
☐ In a Psychiatric Nursing Home
☐ In a Psychiatric Home Care
☐ In a Sheltered housing facility
☐ Other, namely:...

3. How many years are or have you been professionally active as psychiatrist, including as trainee in psychiatry?

☐ Less than 5 years ☐ 6 - 10 years ☐ 11 - 20 years ☐ More than 20 years

4. Have you ever received special training in palliative and/or other end of life care? ☐ Yes ☐ No

5. Do you feel sufficiently competent to be involved in euthanasia procedures as psychiatrist (or trainee)? ☐ Yes ☐ No

6. What is your age? ☐ Younger than 30 ☐ 30 - 40 years ☐ 41 - 60 years ☐ Older than 60 years

7. What is your sex? ☐ Male ☐ Female ☐ X

Part 2: The following questions gauge your engagement in ADULT patients' euthanasia requests that are PRIMARILY BASED on the suffering experiences of a patient with one or more psychiatric disorder(s), other than dementia.

8. Have you ever refused to be actively engaged **as treating physician** in order to have a psychiatric patient's explicit euthanasia request clarified? ☐ Yes ☐ No

9. What was your main motive for (non-) refusal? ....
....

10. Throughout your career, have you been actively engaged in one or more roles concerning explicitly expressed euthanasia requests of adult patients with (a) psychiatric disorder(s)? (More answer options plausible)

☐ No, in not one single role
☐ Yes, as **treating physician**, who refers the own patient to a colleague-physician for further clarification/advise
☐ Yes, as **attending physician**, engaged in the clarification of a euthanasia request of **my own patient**
☐ Yes, as **attending physician**, engaged in the clarification of a euthanasia request of a **colleague-physician's patient**
☐ Yes, as **preliminary advising physician** concerning a partial aspect (e.g. ruling out the existence of an acute depression, assessing mental competence).
☐ Yes, as **procedural advising physician** concerning the legally required 1st or 2nd advice
☐ Yes, as **performing physician**, when being present at, assisting in of carrying out the act of euthanasia in **my own patient** is
☐ Yes, as **performing physician**, when being present at, assisting in of carrying out the act of euthanasia in a **colleague's patient**
☐ Yes, in another role:...

11. During the past 12 months, how often have you been engaged in euthanasia procedures concerning psychiatric patients (in whatever role)?

☐ 0 patients ☐ 1-2 patients ☐ 3-5 patients ☐ 5-9 patients ☐ 10-20 patients ☐ > 20 patients

12. During the past 12 months, how often have you given a **positive and/or negative advice** or **refused to give an advice**?

Positive advice	☐ Not applicable	☐ 1-2 patients	☐ 3-5 patients	☐ More than 5 patients
Negative advice	☐ Not applicable	☐ 1-2 patients	☐ 3-5 patients	☐ More than 5 patients
Advice refused	☐ Not applicable	☐ 1-2 patients	☐ 3-5 patients	☐ More than 5 patients

13. **During the past 5 years**, for how many psychiatric patients have you been engaged as **performing physician**?

☐ 0 patients ☐ 1-2 patients ☐ 3-5 patients ☐ More than 5 patients

14. Have you ever engaged an **external consultation team**, specialised in the clarification of euthanasia requests (e.g. ULteam, Vonkel) in the euthanasia procedure of a psychiatric patient?

☐ No, and I would never consider it
☐ No, but I might consider it once
☐ Yes, namely ....
....

15. For what reason would or wouldn't you rely on such ....

external consultation team?	....

16. Would you like to clarify or explain some of your answers to the abovementioned questions?
....
....
....
....
....
....
....
....
