# Supplementary material for: The engagement of psychiatrists in the assessment of euthanasia requests from psychiatric patients in Belgium: a survey study
Source: BMC Psychiatry. 2020 Aug 8;20:400. doi: 10.1186/s12888-020-02792-w (PMC7414658; doi:10.1186/s12888-020-02792-w)
Supplement: Supplementary file 1 — Additional file 1. [file 12888_2020_2792_MOESM1_ESM.zip › Appendix E_IntroductionLetter_OnlineSurvey_Dutch(1).docx]

**Geachte collega,** *[automatische aanspreking via macrofunctie in Excel]*

15 jaar na de implementatie van de euthanasiewet, wordt het debat toegespitst op de meest kwetsbare patiënten, zoals patiënten die lijden aan één of meerdere psychiatrische aandoeningen. De euthanasiepraktijk bij deze patiëntenpopulatie roept veel vragen op die onvoldoende onderzocht zijn.

Daarom nemen onderzoekers van 4 Vlaamse universiteiten (VUB, UGent, KUL en UA) het initiatief om via een survey bij alle psychiaters in Vlaanderen te peilen naar hun ervaringen en attitudes inzake de euthanasiepraktijk bij deze patiëntenpopulatie.

**Wat vragen zij van u?**

Uw anonieme deelname aan deze survey. Deze bestaat uit een ‘algemeen deel’, dat iedereen kan invullen, en een ‘facultatief deel’ dat u enkel kan invullen indien u tijdens uw loopbaan direct of indirect geconfronteerd geweest bent met minimum één afgeronde euthanasieprocedure van een volwassen patiënt met minstens 1 psychiatrische aandoening. Hierbij speelt de einduitkomst van de procedure geen rol.

**Hoe deelnemen?**
Deelnemen is mogelijk via deze link: <https://ondrzk.nl/ls206/index.php/322383?lang=nl-informal>

En het invoeren van de volgende toegangscode *[automatische token code via macrofunctie in Excel]*

**Hoe lang duurt het invullen?**

De tijd die u nodig heeft om enkel het algemeen dan wel beide delen in te vullen, varieert van 10 tot 15 minuten (deel 1) tot 30 minuten (beide delen).

De onderzoekers danken u alvast voor uw deelname.

Voor alle vragen of opmerkingen, kan u rechtsreeks met hen contact opnemen:

**Drs. Monica Verhofstadt**, 0494 98 09 92, [monica.verhofstadt@vub.be](mailto:monica.verhofstadt@vub.be)
**Prof. dr. Kenneth Chambaere**, 0498 18 45 57, kenneth.chambaere@vub.be
