# Supplementary material for: The engagement of psychiatrists in the assessment of euthanasia requests from psychiatric patients in Belgium: a survey study
Source: BMC Psychiatry. 2020 Aug 8;20:400. doi: 10.1186/s12888-020-02792-w (PMC7414658; doi:10.1186/s12888-020-02792-w)
Supplement: Supplementary file 1 — Additional file 1. [file 12888_2020_2792_MOESM1_ESM.zip › Appendix F_IntroductionLetter_PaperVersionSurvey_Dutch(2).docx]

**Geachte collega,** *[automatische aanspreking via macrofunctie in Word]*

15 jaar na de implementatie van de euthanasiewet, wordt het debat toegespitst op de meest kwetsbare patiënten, zoals patiënten die lijden aan één of meerdere psychiatrische aandoeningen. De euthanasiepraktijk bij deze patiëntenpopulatie roept veel vragen op die onvoldoende onderzocht zijn.

Daarom nemen onderzoekers van 4 Vlaamse universiteiten (VUB, UGent, KUL en UA) het initiatief om via een survey bij alle psychiaters in Vlaanderen te peilen naar hun ervaringen en attitudes inzake euthanasie.

**Wat vragen zij van u?**

Uw anonieme deelname aan deze survey. Deze survey bestaat uit een ‘algemeen deel’, door iedereen in te vullen, en ‘facultatief deel’. Het facultatief deel hoeft u enkel in te vullen indien u tijdens uw loopbaan geconfronteerd geweest bent met minimum één euthanasieverzoek van een volwassen patiënt met minstens 1 psychiatrische aandoening. Hierbij kan het gaan om een patiënt uit uw praktijk of een patiënt die naar u werd doorverwezen.

**Hoe deelnemen?**

Bijgevoegd bij deze brief vindt u de vragenlijsten. Nadat u ze ingevuld heeft, kan u ze ons per post terugsturen via de bijgesloten voorgefrankeerde enveloppe.

U kan ook deelnemen via de online survey. Als u de vragenlijsten liever online invult, kan dit via deze link: <https://ondrzk.nl/ls206/index.php/322383?lang=nl-informal>

En het invoeren van de volgende toegangscode: *[automatische token code via macrofunctie in Word]*

Ook indien u niet wenst deel te nemen, vragen wij u even naar de online tool te gaan en aan te geven waarom u niet deelneemt.

Deelname is volstrekt anoniem. Deze gepersonaliseerde mail werd gegenereerd, gebruikmakend van het VVP-ledenbestand. De onderzoekers kunnen uw antwoorden niet linken aan uw identiteit.

Meer info over anonimiteit en privacy rond dit onderzoek vindt u op de achterkant van deze brief.

**Hoe lang duurt het invullen?**

Zowel het algemene deel als het facultatieve deel tellen slechts 2 bladzijden (1 A4). De tijd die u nodig heeft om één dan wel beide delen in te vullen, varieert van 10 tot 30 minuten.

Wij danken u alvast voor uw deelname.

Voor alle vragen of opmerkingen, kan u contact opnemen met de onderzoekers:

**Drs. Monica Verhofstadt**, 0494 98 09 92, [monica.verhofstadt@vub.be](mailto:monica.verhofstadt@vub.be)
**Prof. dr. Kenneth Chambaere**, 0498 18 45 57, [kenneth.chambaere@vub.be](mailto:kenneth.chambaere@vub.be)

**ANONIMITEIT, PRIVACY EN CONTACTGEGEVENS ONDERZOEKERS**

Dit onderzoek is onderhevig aan de nieuwe Algemene Verordening Gegevensbescherming (AVG) regelgeving. De gegevens die wij verzamelen worden uitsluitend voor dit onderzoek gebruikt en worden volstrekt anoniem gerapporteerd. Ze worden uitsluitend door de onafhankelijke onderzoekers verwerkt en nooit aan derden doorgegeven. Uw deelname is volstrekt anoniem. Op geen enkel ogenblik is het mogelijk om nominatieve gegevens van U en van uw patiënten te identificeren. Sommige achtergrondgegevens (bijvoorbeeld geslacht, leeftijd, aantal jaar beroepservaring) worden enkel gebruikt om een algemeen beeld van de deelnemers als groep te kunnen schetsen. Deze gegevens worden meteen na afloop van de onderzoektermijn vernietigd. De andere, meer inhoudelijke data worden 10 jaar bewaard conform de facultaire Research Data Management (RDM) richtlijnen.

De onderzoekers van de Onderzoeksgroep Zorg rond het Levenseinde (VUB-UGent) hebben de **Vlaamse Vereniging voor Psychiatrie** gevraagd om deze vragenlijst naar u te verzenden en hebben zelf geen toegang gekregen tot hun ledenbestand. Omgekeerd krijgt de VVP nooit toegang tot de ruwe data. De automatisch gegenereerde tokencodes worden enkel gebruikt wordt voor follow-up doeleinden (zodat we diegenen die de vragenlijst reeds hebben ingevuld en doorgestuurd geen reminder sturen) en worden definitief verwijderd bij de verwerking van de gegevens.

**INLEIDING VRAGENLIJST**
Bij de meeste vragen dient u bij het voor u passende antwoord slechts 1 vakje aan te kruisen. Indien u bij een vraag meerdere hokjes mag aankruisen, staat dit duidelijk vermeld. De “open vragen” zonder vastgelegde antwoordmogelijkheden kunnen vrij ingevuld worden.

Mogelijk zullen niet alle vragen voor u van toepassing zijn. In dat geval wordt een instructie gegeven om direct door te gaan naar een vraagnummer verderop. U dient dan de tussenliggende vragen niet in te vullen.

**TERMINOLOGIE**

Doorheen de vragenlijst komen onderstaande termen geregeld terug. In het kader hieronder geven wij u mee wat daaronder verstaan wordt. Deze definities kunnen u helpen om de vragen met een gerust hart te beantwoorden.

**Euthanasie**: Opzettelijke levensbeëindiging op uitdrukkelijk verzoek van de patiënt waarbij de arts een letale dosis aan deze patiënt toedient OF voor de patiënt prepareert zodat deze patiënt het aan zichzelf toedient (medisch begeleide zelfdoding).
**Uitklaring euthanasie**: Tijdens een uitklaring wordt bekeken of een patiënt volgens de arts in aanmerking kan komen voor euthanasie (conform de wettelijke en/of andere zorgvuldigheidsbepalingen).
**Behandelend arts:** De behandelend arts van de patiënt die zich focust op de behandeling van de psychopathologie van de patiënt. Deze arts kan daarnaast betrokken zijn bij de uitklaring van het euthanasieverzoek en/of als uitvoerend arts optreden.
**Uitvoerend arts**: Deze arts kan de euthanasie zelfstandig uitvoeren of mits assistentie van een collega hulpverlener. Deze arts kan ook assisteren of aanwezig zijn bij de euthanasie, bijvoorbeeld door een lethale dosis middelen klaar te zetten die de patiënt vervolgens zelf inneemt (hulp bij zelfdoding).
**Preliminair adviserend arts**: Arts die gevraagd wordt om advies te geven over specifieke criteria i.f.v. het uitdrukkelijk euthanasieverzoek. Het kan daarbij gaan om assessment/evaluatie van wilsbekwaamheid, aanwezigheid/uitsluiten van een depressie, etc.
**Procedureel adviserend arts**: De arts die ten behoeve van de euthanasieprocedure en op verzoek van de uitvoerend arts, gevraagd wordt om de naleving van de wettelijke en klinische zorgvuldigheidscriteria bij de patiënt na te gaan en de resultaten hiervan schriftelijk aan de uitvoerend arts rapporteert.
