# Supplementary material for: The engagement of psychiatrists in the assessment of euthanasia requests from psychiatric patients in Belgium: a survey study
Source: BMC Psychiatry. 2020 Aug 8;20:400. doi: 10.1186/s12888-020-02792-w (PMC7414658; doi:10.1186/s12888-020-02792-w)
Supplement: Supplementary file 1 — Additional file 1. [file 12888_2020_2792_MOESM1_ESM.zip › Appendix G_Data Analysis Plan VVP_fullversion.docx]

# Belgian Psychiatrist’s Attitudes and Experiences with Psychiatric Euthanasia Requests and Procedures

Verhoftstadt M., Audenaert K., Van den Broeck K., Deliens L., Mortier F., Pardon K., De Bacquer D., & Chambaere K.

Table of contents

[Belgian Psychiatrist’s Attitudes and Experiences with Psychiatric Euthanasia Requests and Procedures 1](#_Toc36651148)

[Research questions 3](#_Toc36651149)

[Nature and Structure of the Dataset 4](#_Toc36651150)

[Structural Models 4](#_Toc36651151)

[Analysis plan preparation in SPSS 5](#_Toc36651152)

[Data import 5](#_Toc36651153)

[Data Cleaning 5](#_Toc36651154)

[1. Value Labels 5](#_Toc36651155)

[1) Participants and non-response 5](#_Toc36651156)

[2) Survey questions with Yes/No or Yes/Not Selected responses 6](#_Toc36651157)

[3) Variables regarding professional background and socio-demographics 6](#_Toc36651158)

[4) Statements on euthanasia 6](#_Toc36651159)

[5) Variables concerning concrete experiences with psychiatric euthanasia requests 7](#_Toc36651160)

[6) Variables concerning psychiatrists’ and their trainees’ opinions on difficulties with the assessment of the legal criteria 7](#_Toc36651161)

[7) Variables concerning whether or not the patient died by means of euthanasia 7](#_Toc36651162)

[2. Recoding variables 8](#_Toc36651163)

[Data Analysis: Sample + Research Questions 9](#_Toc36651164)

[1. Descriptive Statistics 9](#_Toc36651165)

[1.1. Sample 9](#_Toc36651166)

[1.2. Answers to the research questions 10](#_Toc36651167)

[RQ 1: What are (assistant-) psychiatrists’ attitudes towards the euthanasia practice? 10](#_Toc36651168)

[RQ 2: To what extent can (assistant-)psychiatrists conceive of themselves refusing, granting and/or performing euthanasia for psychiatric patients? 11](#_Toc36651169)

[RQ 3: What are (assistant-)psychiatrists’ experiences with the euthanasia practice? 13](#_Toc36651170)

[RQ4: How do (assistant-)psychiatrists handle requests for euthanasia from psychiatric patients? 15](#_Toc36651171)

[2. Descriptive Statistics by Group 17](#_Toc36651172)

[RQ 5. To what extent are (assistant-)psychiatrists’ attitudes, conceivability, experience and handling of euthanasia requests for psychiatric patients related to their socio-demographic and professional background? 17](#_Toc36651173)

[G*Power: How many respondents do I need? 18](#_Toc36651174)

[G*Power: How many respondents do I need? 19](#_Toc36651175)

[G*Power: How many respondents do I need? 21](#_Toc36651176)

[G*Power: How many respondents do I need? 22](#_Toc36651177)

[Syntax 22](#_Toc36651178)

[1. Data import Online Survey: in Dutch 22](#_Toc36651179)

[2. Syntax psychiatrists’ attitudes and readiness to engage 50](#_Toc36651180)

[3. Data import psychiatrists’ concrete experiences 53](#_Toc36651181)

[4. Data import: case study 57](#_Toc36651182)

# Research questions

1. What are (assistant-)psychiatrists’ attitudes towards the euthanasia practice?
   1.1. What are (assistant-)psychiatrists’ attitudes towards the euthanasia practice in general
   1. What are (assistant-)psychiatrists’ attitudes towards euthanasia practice in psychiatric patients specifically?
2. To what extent can (assistant-)psychiatrists conceive of themselves refusing, granting and/or performing euthanasia for psychiatric patients?
3. What are (assistant-)psychiatrists’ experiences with the euthanasia practice?

3.1. During the last year, how often have (assistant-)psychiatrists been confronted with euthanasia requests from psychiatric patients?

3.2 Throughout their career, how often have (assistant-)psychiatrists been confronted with euthanasia requests from psychiatric patients?

3.3. How often have they been involved in the management of psychiatric euthanasia procedures?

3.4. To what extent have they been involved in the management of psychiatric euthanasia procedures?

3.5. To what extent and in what capacity have they been involved in the management of psychiatric euthanasia procedures?

1. How do (assistant-)psychiatrists handle requests for euthanasia from psychiatric patients?

4.1. Which other important actors (family, friends and other caregivers) are or can be involved during the euthanasia decision making procedures?

4.2. Are there specific needs to be distinguished that could help (assistant-)psychiatrists adequately address the complexity of euthanasia requests and procedures?

1. To what extent are (assistant-)psychiatrists’ attitudes, conceivability, experience and handling of euthanasia requests for psychiatric patients related to their socio-demographic and professional background?

5.1. To what extent are (assistant-)psychiatrists’ attitudes regarding psychiatric euthanasia requests and procedures related to their socio-demographic and professional background?

5.2. To what extent are (assistant-)psychiatrists’ conceivability toward the handling of euthanasia requests for psychiatric patients related to their socio-demographic and professional background?

5.3. To what extent are (assistant-)psychiatrists’ experiences with psychiatric euthanasia requests and procedures related to their socio-demographic and professional background?

5.4 To what extent are (assistant-)psychiatrists’ own accounts of psychiatric euthanasia decision-making related to their socio-demographic and professional background?

# Nature and Structure of the Dataset

The data set contains 175 variables, of which:
1) one concerning the informed consent
2) seven concerning a short non-response questionnaire, and
3) 168 variables that directly fit the given answer modalities to the survey questions

The list of variables in Dutch, French and English can be found in the codebook (see additional document).

## Structural Models

1. Explorative study: descriptive statistics concerning (assistant-)psychiatrists’ characteristics, attitudes and experiences regarding psychiatric euthanasia requests and procedures
2. Analysis comparing at least two independent groups and their specific attitudes and experiences regarding psychiatric euthanasia requests and procedures

Gender and age category

Attitudes

Professional Background

# Analysis plan preparation in SPSS

## Data import

1. The raw data and the syntax file were automatically extracted from Limesurvey and inserted in two Excel-files and two SPSS-files: one Excel and SPSS-file contained full responses (e.g. “Ja” and “Nee”) and the other Excel and SPSS-file contained coded responses (e.g. “Y” and “2”).
2. Data received from the postal survey versions were then manually added to both SPSS-files and checked for errors in data entry via descriptive analysis.
3. The .sav dataset with raw data (full responses) was chosen in order to ensure that no data were falsely considered as missing values as the coded excel-file and SPSS-file only had labeled “Y” for “Yes” and “ ” (empty space) for “No”.

## Data Cleaning

##### 1. Value Labels

Data labeling was realized automatically by means of running the extracted syntax-file from LimeSurvey into the extracted .sav (See Appendix 2)

###### 1) Participants and non-response

To easily distinguish the group of participants who agreed or did not agree on the terms and conditions set in the informed consent introduction letter, the variable “IC” was labeled as follows:

VALUE LABELS IC
 1 "Ja, ik ga akkoord met deelname."
 0 "Nee, ik ga niet akkoord met deelname.".

All answers to the short non-response questionnaire were labeled as:

VALUE LABELS NonPartSurvNL
1 "Ja"
0 "Nee".

###### 2) Survey questions with Yes/No or Yes/Not Selected responses

Most variables contain bivariate Yes/No questions, with the following data structure: Yes/No or Yes/Not Selected (in case of already coded responses. “Y” for the response “Yes” and “ “ (empty) for the response “No”.)
To ensure that the “No” responses were not considered as missing values, the variables were automatically labeled via the extracted syntax-file from LimeSurvey.

For example:

| VALUE LABELS WorkasPsych 1 "Ja" 0 "Nee". | VALUE LABELS ExtConsTeam  1 "Ja"  0 "Niet geselecteerd". |
| --- | --- |

###### 3) Variables regarding professional background and socio-demographics

| **Value labels** | **Value labels** | **Value labels** | **Value labels** | **Value labels** |
| --- | --- | --- | --- | --- |
| YrsAsPsych  0 "Minder dan 5 jaar"  1 "6 tot 10 jaar"  2 "11 tot 20 jaar"  3 "Meer dan 20 jaar". | SpecialEducEOL  1 "Ja"  0 "Nee". | CompetenceEUNL  1 "Ja"  0 "Nee". | AgeNL  0 "Jonger dan 30"  1 "30 - 40 jaar"  2 "41 - 60 jaar"  3 "Ouder dan 60". | SexNL  0 "Man"  1 "Vrouw"  2 "X". |

###### 4) Statements on euthanasia

These variables were assessed by means of a 5-point Likert Scale and coded as follows:

VALUE LABELS STOnlyTermNL

1 "Helemaal oneens"

2 "Oneens"

3 "Neutraal"

4 "Eens"

5 "Helemaal eens".

###### 5) Variables concerning concrete experiences with psychiatric euthanasia requests

Variables measuring the number of patients the (assistant-)psychiatrist was confronted with in the different stages of the euthanasia procedure (request/advice/performance)

| NumberEuReqN | OftenAdv | NumberEUPerformNL |
| --- | --- | --- |
| 0 "0 patiënten"  1 "1-2 patiënten"  2 "3-5 patiënten"  3 "5-9 patiënten"  4 "10-20 patiënten"  5 "> 20 patiënten". | "A1" "niet van toepassing"  "A2" "1-2 patiënten"  "A3" "3-5 patiënten"  "A4" "meer dan 5 patiënten". | 0 "0 patiënten"  1 "1-2 patiënten"  2 "3-5 patiënten"  3 "meer dan 5 patiënten". |

###### 6) Variables concerning psychiatrists’ and their trainees’ opinions on difficulties with the assessment of the legal criteria

VALUE LABELS OpinOfPtScaleNL

1 "Geen"

2 "Weinig"

3 "Gemiddeld"

4 "Veel"

5 "Erg Veel".

###### 7) Variables concerning whether or not the patient died by means of euthanasia

VALUE LABELS DeathEuNL

0 "Weet ik niet"

1 "Ja"

2 "Nee, de euthanasieprocedure is nog niet afgerond"

3 "Nee, de patiënt trok het euthanasieverzoek zonder druk van derden terug in"

4 "Nee, de patiënt trok het euthanasieverzoek onder druk van derden terug in"

5 "Nee, de patiënt is op een andere manier overleden"

6 "Nee, wegens een andere dan de hierboven genoemde redenen".

##### 2. Recoding variables

**New variable “AttProEu”**

The variable regarding the “13 Statements” will be computed into a new variable “AttProEU” in terms of a more permissive versus dismissive approach toward euthanasia in psychiatric patients. Statement 5 on assisted-suicide versus euthanasia and statement 13 on current overly permissive approaches were excluded.

**Measurement Model**

Statement No. 3,4,6,7,8,9,11,12

Reverse Statements No. 1,2,10

**New variable: Future Roles**

The nominal variable regarding the “Future Roles” will be computed into a new ordinal variable “ConcRole” that shows the extent or level of respondents’ conceivable involvement in psychiatric euthanasia. The hierarchy was defined by the researchers: see measurement model.

**Measurement Model**

ConcRole

0 = No Role

1 = Referring Psych

2 = Preliminary Advising Psych

3 = Legally required advising Psych

4 = Attending Psych (clarify request)
5 = Performing Psych

Future Roles No. 1-8

# Data Analysis: Sample + Research Questions

## Descriptive Statistics

##### Sample

To produce the contents for the descriptive statistics to describe the sample, the following variables will be analyzed:

| **Main Variable** | **Associated Question** | **Values** | **Sub-variable** | **Variable**  **Type** |
| --- | --- | --- | --- | --- |
| IC | See CodeBook | 1= Yes 2= No | / | Nominal |
| NonPartSurvNL | See CodeBook | 1= Yes  0 = No | NonPartSurvNL_SQ001 NonPartSurvNL_SQ002 NonPartSurvNL_SQ003  NonPartSurvNL_SQ004 NonPartSurvNL_SQ005 NonPartSurvNL_SQ006  NonPartSurvNL_other | Nominal |
| WorkasPsychNL | Was u de voorbije 12 maanden werkzaam als (assistent-) psychiater van volwassen patienten? | 1= Yes  0 = No |  | Nominal |
| WhereWorkNL | Was u in deze periode als psychiater werkzaam |  | WhereWorkNL_WorkPrivNL  WhereWorkNL_WorkHospNL  WhereWorkNL_WorkCentNL  WhereWorkNL_WorkPyschCareNL  WhereWorkNL_WorkPsychHomeNL  WhereWorkNL_WorkAsstLivNL  WhereWorkNL_WorkAsstOthNL | Nominal  Nominal  Nominal  Nominal  Nominal  Nominal Nominal |
|  |  |  | SpecWhereWork* | String |
| YrsAsPsychNL | Sinds hoeveel jaren bent u als psychiater werkzaam (geweest), inclusief als assistent? | 0 = Minder dan 5 jaar 1= 6 tot 10 jaar 2 = 11 tot 20 jaar 3 = Meer dan 20 jaar |  | Ordinal |
| SpecialEducEOLNL | Heeft u een gespecialiseerde opleiding gevolgd rond palliatieve en/of andere levenseindezorg? | 1 = Yes  0 = No |  | Nominal |
| CompetenceEUNL | Voelt u zich voldoende competent om als psychiater betrokken te worden bij een euthanasieprocedure? | 1 = Yes  0 = No |  | Nominal |
| AgeNL | Wat is uw leeftijd? | 0 = Jonger dan 30  1 = 30 - 40 jaar  2 = 41 - 60 jaar  3 = Ouder dan 60 |  | Ordinal |
| SexNL | Wat is uw geslacht? | 0 = Man  1 = Vrouw  2 = X |  | Nominal |

*This variable contains a follow-up question to ‘WhereWorkNL_WorkAsstOthNL’ and answered as free text, which requires further analysis in order to make the decision whether or not to include the respondent in the sample.

##### Answers to the research questions

###### RQ 1: What are (assistant-) psychiatrists’ attitudes towards the euthanasia practice?

- 1. What are (assistant-)psychiatrists’ attitudes towards the euthanasia practice in general?
  2. What are (assistant-)psychiatrists’ attitudes towards euthanasia practice in psychiatric patients specifically?

Analysis

- Analyze the “opinion” questions (13 Statements) by determining the distribution for each variable
  - For these variables, the coding is as follows:
    - 1= helemaal oneens
    - 2 = oneens
    - 3 = neutraal
    - 4 = eens
    - 5 = helemaal eens
  - Variables to be included:

| **Variable** | **Corresponding Question** | **RQ** | **Variable type** |
| --- | --- | --- | --- |
| STOnlySomatNL | Euthanasie zou voor niet-terminale patiënten wettelijk mogelijk moeten zijn, maar enkel als er sprake is van een somatische aandoening. | 1.1. | Scale |
| STNotPsychNL | Euthanasie zou voor patiënten met een psychiatrische aandoening wettelijk mogelijk moeten blijven. | 1.1. 1.2. | Scale |
| STYesPsychHelpRelNL | Euthanasie bij psychiatrische patiënten is verenigbaar met een psychiatrische hulpverleningsrelatie. | 1.1. 1.2. | Scale |
| STMAIDNL | Bij psychiatrische patiënten is medisch begeleide zelfdoding (patiënt neemt zelf de letale dosis in, in aanwezigheid en onder begeleiding van de arts) voor mij meer aanvaardbaar dan euthanasie (arts dient letale dosis toe aan de patiënt). | 1.1. 1.2. | Scale |
| STHopelessNL | Een psychiatrische patiënt kan zich in een medisch uitzichtloze toestand bevinden. | 1.1. 1.2. | Scale |
| STExtremeSuffNL | Een psychiatrische patiënt kan ondraaglijk lijden. | 1.1. 1.2. | Scale |
| STNoPerpectiveNL | Een redelijk behandelperspectief kan ontbreken bij een psychiatrische patiënt. | 1.1. 1.2. | Scale |
| STEuthPrevSuicNL | Euthanasie is een aanvaardbaar alternatief om suïcide te voorkomen. | 1.1. | Scale |
| STFutureMedOptionsNL | Bij de uitklaring van een euthanasieverzoek moet rekening gehouden worden met mogelijke effectieve therapeutische ontwikkelingen in de toekomst. | 1.1. | Scale |
| STWholeLifeNL | Bij de uitklaring van een euthanasieverzoek dient de focus niet louter op de medische, maar op de volledige leefsituatie van de patiënt gelegd te worden. | 1.1. | Scale |
| STWellConsiderNL | Een doodswens van een psychiatrische patiënt kan weloverwogen zijn en niet louter een symptoom van een onderliggende pathologie. | 1.1. 1.2. | Scale |
| STTooPermissiveNL | In bepaalde gevallen wordt er te lichtzinnig omgegaan met de mogelijkheid van euthanasie voor psychiatrische patiënten. | 1.1. 1.2. | Scale |

##

###### RQ 2: To what extent can (assistant-)psychiatrists conceive of themselves refusing, granting and/or performing euthanasia for psychiatric patients?

Analysis 2.1.: refused euthanasia request

- - Analyze the answers to the questions 9, 10 and 15 of the ‘general part’ by determining the distribution for the variable corresponding with question 9 and 15, and qualitatively examining the variable corresponding with open question 10
  - Variables to be included:

| **Variable** | **Corresponding Question** | **Value** | **Variable type** |
| --- | --- | --- | --- |
| RefEuNL | Heeft u als behandelend arts van een psychiatrische patiënt ooit geweigerd om actief betrokken te zijn bij de uitklaring van diens uitdrukkelijk euthanasieverzoek? | 1=Yes;  0 = No | Nominal |
| WhyRefEuNL | Om welke reden(en) heeft u hiertoe besloten? | Free tekst* | String |
| FutureRoles | Acht u het denkbaar dat u in de toekomst één of meer van volgende rollen opneemt in het kader van concrete euthanasieverzoeken van volwassen patiënten met (een) psychiatrische aandoening(en). | 1=Yes;  0 = No | Nominal |
| FutureRolesNL[SQ009] | Nee, in geen enkele rol | 1=Yes;  0 = No | Nominal |

* Ensure that the variable is analyzed with respect to the answer given to RefEuNL, as data exist both for those that have responded Yes and No to the primary variable

Analysis 2.2.: potentially being involved and granting a euthanasia request

- - Analyze the majority of the answers to question 15 of the ‘general part’ by determining the distribution for the corresponding variable except for the open answer option that needs to be examined by means of a qualitative approach
  - (Sub-)Variables to be included:

| **(sub)Variable** | **Corresponding Question** | **Value** | **Variable type** |
| --- | --- | --- | --- |
| FutureRoles | Acht u het denkbaar dat u in de toekomst één of meer van volgende rollen opneemt in het kader van concrete euthanasieverzoeken van volwassen patiënten met (een) psychiatrische aandoening(en). (Meerdere opties mogelijk) | 1=Yes;  0 = No | Nominal |
| FutureRolesNL[SQ009] | Nee, in geen enkele rol | 1=Yes;  0 = No | Nominal |
| FutureRolesNL[SQ001] | ja, als behandelend arts, die de eigen patiënt doorverwijst naar een collega-arts voor de verdere uitklaring/advies | 1=Yes;  0 = No | Nominal |
| FutureRolesNL[SQ002] | ja, als behandelend arts, die de uitklaring van het euthanasieverzoek van een eigen patiënt zelf opneemt | 1=Yes;  0 = No | Nominal |
| FutureRolesNL[SQ003] | ja, als arts die de uitklaring van het euthanasieverzoek van een patiënt van een collega-arts opneemt | 1=Yes;  0 = No | Nominal |
| FutureRolesNL[SQ004] | ja, als preliminair adviserend arts over een deelaspect (bv. uitsluiten acute depressie, beoordelen wilsbekwaamheid) | 1=Yes;  0 = No | Nominal |
| FutureRolesNL[SQ005] | ja, als procedureel adviserend arts in het kader van een wettelijk vereist 1ste of 2de euthanasie-advies | 1=Yes;  0 = No | Nominal |
| FutureRolesNL[SQ006] | ja, als uitvoerend arts, die de euthanasie bij een eigen patiënt uitvoert, hierbij assisteert of aanwezig is | 1=Yes;  0 = No | Nominal |
| FutureRolesNL[SQ008] | ja, in een andere rol, nl | 1=Yes;  0 = No | Nominal |
| SpecFutureRoleNL | In welke rol? | Free text | String |

Analysis 2.3.: potentially granting a euthanasia request

- - Analyze the remaining answers to question 15 of the ‘general part’ by determining the distribution for the corresponding variable except for the open answer option that needs to be examined by means of a qualitative approach
  - Variables to be included:

| **Variable** | **Corresponding Question** | **Value** | **Variable type** |
| --- | --- | --- | --- |
| FutureRolesNL[SQ007] | ja, als uitvoerend arts, die de euthanasie bij een patiënt van een collega-arts uitvoert, hierbij assisteert of aanwezig is | 1=Yes;  0 = No | Nominal |
| SpecFutureRoleNL | In welke rol? | Free text | String |

###### RQ 3: What are (assistant-)psychiatrists’ experiences with the euthanasia practice?

Analysis 3.1.: how often have (assistant-)psychiatrists been confronted with euthanasia requests from psychiatric patients during the last year?

- - Analyze the answers to question 12 of the ‘general part’ by determining the distribution for the corresponding variable
  - Variable to be included:

| **Variable** | **Corresponding Question** | **Value** | **Variable type** |
| --- | --- | --- | --- |
| NumberEuReqNL | Bij hoeveel euthanasieverzoeken van dergelijke patiënten werd u de voorbije 12 maanden (in eender welke professionele rol) betrokken? | 0 = 0 patiënten  1 = 1-2 patiënten  2 = 3-5 patiënten  3 = 5-9 patiënten  4 = 10-20 patiënten  5 = > 20 patiënten | Ordinal |

Analysis 3.2.: How often have (assistant-)psychiatrists been confronted with euthanasia requests from psychiatric patients throughout their career?

- - Analyze the answers to question 11 of the ‘general part’ by determining the distribution for the corresponding variable ‘InvolvEU’, except for the open answer option that needs to be examined by means of a qualitative approach. The sub-variables correspond to the question: Werd u tijdens uw loopbaan als psychiater ooit betrokken bij een uitdrukkelijk euthanasieverzoek van een volwassen patiënt die hoofdzakelijk leed aan 1 of meer psychiatrische aandoening(en)?
  - Sub-variables to be included:

| **Variable** | **Corresponding Question** | **Value** | **Variable type** |
| --- | --- | --- | --- |
| InvolvEuNL_0 | nee, ik werd hier nooit mee geconfronteerd | 1=Yes;  0 = No | Nominal |
| InvolvEuNL_1 | ja, als behandelend arts, die de eigen patiënt doorverwees naar een collega-arts i.f.v. een euthanasieprocedure | 1=Yes;  0 = No | Nominal |
| InvolvEuNL_2 | ja, als behandelend arts, die de uitklaring van het euthanasieverzoek van een eigen patiënt zelf opnam | 1=Yes;  0 = No | Nominal |
| InvolvEuNL_3 | ja, als arts die de uitklaring van het euthanasieverzoek van een patiënt van een collega-arts opnam | 1=Yes;  0 = No | Nominal |
| InvolvEuNL_4 | ja, als preliminair adviserend arts over een deelaspect (bv. uitsluiten acute depressie, beoordelen wilsbekwaamheid) | 1=Yes;  0 = No | Nominal |
| InvolvEuNL_5 | ja, als procedureel adviserend arts in het kader van een wettelijk vereist vereist 1ste of 2de euthanasie-advies | 1=Yes;  0 = No | Nominal |
| InvolvEuNL_6 | ja, als uitvoerend arts die euthanasie bij een eigen patiënt uitvoerde, hierbij assisteerde of aanwezig was | 1=Yes;  0 = No | Nominal |
| InvolvEuNL_7 | ja, als uitvoerend arts die euthanasie bij een patiënt van een collega-arts uitvoerde/assisteerde of erbij aanwezig was | 1=Yes;  0 = No | Nominal |
| InvolvEuNL_8 | ja, in een andere rol | 1=Yes;  0 = No | Nominal |
| SpecInvolNL | Welke andere rol? | Free text | String |

Analysis 3.3-3.5.: How often, to what extent and in what capacity have (assistant-)psychiatrists been involved in the management of psychiatric euthanasia procedures?

- - Analyze the answers to question 12, 13, 14 and 16 of the ‘general part’ by determining the distribution for the corresponding variables, except for the open answer option that needs to be examined by means of a qualitative approach.
  - Variables to be included:

| **Variable** | **Corresponding Question** | **Value** | **Variable type** |
| --- | --- | --- | --- |
| NumberEuReqNL | Bij hoeveel euthanasieverzoeken van dergelijke patiënten werd u de voorbije 12 maanden (in eender welke professionele rol) betrokken? | 0 = 0 patiënten;  1 = 1-2 patiënten;  2 = 3-5 patiënten;  3 = 5-9 patiënten;  4 = 10-20 patiënten;  5 = > 20 patiënten; | Ordinal |
| OftenAdv  OftenAdv [SQ001]  OftenAdv [SQ002]  OftenAdv [SQ003] | Hoe vaak heeft u de voorbije 12 maanden een positief dan wel negatief advies gegeven of een advies geweigerd? | A1= niet van toepassing;  A2 = 1-2 patiënten;  A3 = 3-5 patiënten;  A4 = meer dan 5 patiënten | Ordinal |
| NumberEUPerform | Bij hoeveel psychiatrische patiënten was u de afgelopen 5 jaar als uitvoerend arts betrokken? | 0 "0 patiënten"  1 "1-2 patiënten"  2 "3-5 patiënten"  3 "meer dan 5 patiënten". | Nominal |
| ExtConsTeam* ExtConsTeam [SQ001]  ExtConsTeam [SQ002]  ExtConsTeam [SQ003] | Heeft u ooit een extern consultatieteam, gespecialiseerd in de uitklaring van euthanasieverzoeken (bv. ULteam, Vonkel) betrokken bij de euthanasieprocedure van een psychiatrische patiënt? | 0 = Nee, en ik zou dat ook nooit doen; 1 = Nee, maar het is wel denkbaar dat ik dat ooit doe 2 = Ja | Ordinal |
| WhyNoExtConsTeam | Om welke reden(en) zou u al dan niet beroep op een extern consultatieteam doen? | Free tekst | String |

** Note: for this variable, the coding is listed as “more than one answer possible,” however the phrasing of the question indicates that only one answer should be possible, so ensure that each individual provided one answer for this question. Furthermore, the sub-variables are falsely listed as stand-alone variables in Limesurvey. Hence, this variable needs to be recoded and checked for mutual exclusion.

###### RQ4: How do (assistant-)psychiatrists handle requests for euthanasia from psychiatric patients?

See also 3.3.-3.5.

| **Variable** | **Corresponding Question** | **Value** | **Variable type** |
| --- | --- | --- | --- |
| ExtConsTeam** ExtConsTeam [SQ001]  ExtConsTeam [SQ002]  ExtConsTeam [SQ003] | See also 3.3.-3.5. | See also 3.3.-3.5. | See also 3.3.-3.5. |
| WhyNoExtConsTeam | See also 3.3.-3.5. | See also 3.3.-3.5. | See also 3.3.-3.5. |

Analysis 4.1: Which other important actors (family, friends and other caregivers) are ~~or can be~~ involved during the euthanasia decision making procedures?

- - Analyze the answers to question 8 and 9 of the ‘facultative part’ by determining the distribution for the corresponding variables, except for the open answer option that needs to be examined by means of a qualitative approach.
  - Variables to be included:

| **(Sub-)Variable** | **Corresponding Question** | **Value** | **Variable type** |
| --- | --- | --- | --- |
| ConsCaregivers | Heeft u één of meerdere artsen/hulpverleners/instanties geconsulteerd tijdens de besluitvorming? (meerdere opties mogelijk) | 1=Yes;  0 = No  1=Yes;  0 = No | Nominal  Nominal |
| ConsCaregiversNL[1] | Neen |  |  |
| ConsCaregiversNL[2] | Ja, de huisarts(en) van de patiënt |  |  |
| ConsCaregiversNL[3] | Ja, andere arts(en) van de patiënt |  |  |
| ConsCaregiversNL[4] | Ja, onafhankelijke collega-psychiater(s) |  |  |
| ConsCaregiversNL[5] | Ja, onafhankelijke LEIF-arts(en) |  |  |
| ConsCaregiversNL[6] | Ja, onafhankelijke arts(en) van levenseinde consultatieteam |  |  |
| ConsCaregiversNL[7] | Ja, verpleegkundige(n) |  |  |
| ConsCaregiversNL[8] | Ja, ethische commissie |  |  |
| ConsCaregiversNL[9] | Ja, andere interne adviescommissie |  |  |
| ConsCaregiversNL[10] | Ja, psycholo(o)gen |  |  |
| ConsCaregiversNL[11] | Ja, palliatief zorgteam |  |  |
| ConsCaregiversNL[12]  ConsCaregiversNL_13 | Ja, een (psycho-)sociale dienst  Ja, andere |  |  |
| WhichOtherPrContNL | Welke andere hulpverleners? | Free text | String |
| ConsFamFriNL | Heeft u overleg gehad met familie en/of vrienden van de patiënt? (meerdere antwoorden mogelijk) | / | / |
| ConsFamFriNL[1]  ConsFamFriNL[2]  ConsFamFriNL[3]  ConsFamFriNL[4] | Neen, geen familie of vrienden betrokken  Neen, patiënt had geen familie of vrienden  Ja, tijdens euthanasieprocedure  Ja, na afronding van de euthanasieprocedure | 1=Yes;  0 = No | Nominal |

Analysis 4.2: Are there specific needs to be distinguished that could help (assistant-)psychiatrists adequately address the complexity of euthanasia requests and procedures?

- - Analyze the answers to question 11, 12, 13 of the ‘facultative part’ by determining the distribution for the corresponding variables, except for the open answer option that needs to be examined by means of a qualitative approach.
  - Variables to be included: OpinOfPtScaleNL, TypePressNL, ConfExperNL and SelfEmoSuppNL, respectively corresponding to the questions: “Hoeveel moeilijkheden heeft u ervaren om onderstaande criteria te kunnen beoordelen?”, “Werd u tijdens deze euthanasieprocedure geconfronteerd met onderstaande vormen van druk?”, “Werd u tijdens deze euthanasieprocedure geconfronteerd met onderstaande ervaringen?” and “Heeft u voor uzelf emotionele ondersteuning gezocht tijdens of na de euthanasieprocedure?”
  - Other variables to be included: CaseInfFutNL, OpinChangeNL and AddInfoPartTwoNL

| **Variable** | **Corresponding Question** | **Value** | **Variable type** |
| --- | --- | --- | --- |
| OpinOfPtScaleNL[MentCompNL]  OpinOfPtScaleNL[IncurCondNL] OpinOfPtScaleNL[UnbearSuffNL]  OpinOfPtScaleNL[HopMedCondNL]  OpinOfPtScaleNL[LackResTherNL]  OpinOfPtScaleNL[VolunReqNL] | Mentale competentie (wilsbekwaamheid)  Ongeneeslijkheid van de aandoening  Ondraaglijkheid van het lijden  Uitzichtloosheid van de medische conditie  Ontbreken redelijk therapeutisch perspectief  Vrijwillig, duurzaam en weloverwogen verzoek | 1= None;  2= Little;  3= Somewhat;  4= Much;  5= A great deal | Scale |
| OtherOpinTextNL | Andere, nl.:………… | Free text | String |
| TypePressNL[Press3PartNL]  TypePressNL[PressPtNL]  TypePressNL[PressFamYesNL]  TypePressNL[PressFamNoNL]  TypePressNL[PressCollNoNL]  TypePressNL[PressCollYesNL]  TypePressNL[PressCarInstNoNL]  TypePressNL[PressCarInstYesNL] | Patiënt onder druk van derden  Druk van patiënt voor euthanasie  Druk van naasten voor euthanasie  Druk van naasten tegen euthanasie  Druk van collega’s tegen euthanasie  Druk van collega’s voor euthanasie  Druk van zorginstelling tegen euthanasie  Druk van zorginstelling voor euthanasie | 1=Yes;  0 = No | Nominal |
| ConfExperNL[SelfEmoBurdNL]  ConfExperNL[NewTherChanNL]  ConfExperNL[PtLowSuicRiskNL]  ConfExperNL[RestRelatNL]  ConfExperNL[OthPtAlsoEuNL] | Hoge emotionele belasting voor uzelf  Nieuwe therapeutische kansen bij de patient  Verlaagd risico op suïcide bij de patiënt  Herstel relaties tussen patiënt en diens naasten  Mede-patiënten die ook om euthanasie verzochten | 1=Yes;  0 = No | Nominal |
| SelfEmoSuppNL[1]  SelfEmoSuppNL[2]  SelfEmoSuppNL[3]  SelfEmoSuppNL[4]  SelfEmoSuppNL[5] | Nee  ja, in privékring  ja, bij collega’s  ja, bij externe professionele hulpverlening  ja, bij anderen | 1=Yes;  0 = No " | Nominal |
| CaseInfFutNL | Heeft deze casus uw houding ten aanzien van toekomstige verzoeken beïnvloed? | 1=Yes;  0 = No | Nominal |
| OpinChangeNL | Op welke manier heeft het uw houding veranderd? | Free text | String |
| AddInfoPartTwoNL | Wil u nog iets verduidelijken of toelichten over uw ervaring/gevoelens m.b.t. deze casus? | Free text | String |

Answers to the abovementioned RQ can also be found in the following variables from the ‘general part’ of the questionnaire.

| **Variable** | **Corresponding Question** | **Value** | **Variable type** |
| --- | --- | --- | --- |
| WhyNoExtConsTeam | Om welke reden(en) zou u al dan niet beroep op een extern consultatieteam doen? | Free text | String |
| AddInfoPartOneNL | Wil u nog iets verduidelijken of toelichten? | Free text | String |

## Descriptive Statistics by Group

###### RQ 5. To what extent are (assistant-)psychiatrists’ attitudes, conceivability, experience and handling of euthanasia requests for psychiatric patients related to their socio-demographic and professional background?

Analysis 5.1: To what extent are (assistant-)psychiatrists’ attitudes regarding psychiatric euthanasia requests and procedures related to their socio-demographic and professional background?

- - Analysis of the distribution of the dependent variable: ordinal or interval variable
  - Analysis of subsets and groups.
  - Variables to be included:

| **Variable** | **Variable Type** | **Variable** | **Variable Type** | **Analyses** |
| --- | --- | --- | --- | --- |
| Sex | 2 independent groups | 13 Statements | Ordinal  Interval | MannWhitney/Kolmogorov Smirnov  Onafhankelijke t-toets |
| Age | > 2 independent groups | 13 Statements | Ordinal  Interval | Kruskal Wallis  ANOVA |
| YrsAsPsych | > 2 independent groups | 13 Statements | Ordinal Interval | Kruskal Wallis  ANOVA |
| SpecialEducEOL | 2 independent groups | 13 Statements | Ordinal  Interval | MannWhitney/Kolmogorov Smirnov  Onafhankelijke t-toets |
| CompetenceEU | 2 independent groups | 13 Statements | Ordinal  Interval | MannWhitney/Kolmogorov Smirnov  Onafhankelijke t-toets |
| WorkasPsych | 2 independent groups | 13 Statements | Ordinal  Interval | MannWhitney/Kolmogorov Smirnov  Onafhankelijke t-toets |
| WhereWork | > 2 independent groups | 13 Statements | Ordinal  Interval | Kruskal Wallis  ANOVA |

| **Variable** | **Purpose** | **Variable** | **Variable Type** | **Analyses** |
| --- | --- | --- | --- | --- |
| Age | Correlation | 13 Statements | Ordinal  Interval | Kendall’s tau/Spearman’s R  Pearson’s R |
| YrsAsPsych | Correlation | 13 Statements | Ordinal Interval | Kendall’s tau/Spearman’s R  Pearson’s R |
| SpecialEducEOL | Correlation | 13 Statements | Ordinal  Interval | Kendall’s tau/Spearman’s R  Pearson’s R |
| CompetenceEU | Correlation | 13 Statements | Ordinal  Interval | Kendall’s tau/Spearman’s R  Pearson’s R |
| WhereWork | Correlation | 13 Statements | Ordinal  Interval | Kendall’s tau/Spearman’s R  Pearson’s R |

G*Power: How many respondents do I need?

**t tests** - Means: Difference between two independent means (two groups)

**Analysis:** A priori: Compute required sample size

**Input:** Tail(s) = Two

Effect size d = 0,5

α err prob = 0,05

Power (1-β err prob) = 0,85

Allocation ratio N2/N1 = 1

**Output:** Noncentrality parameter δ = 3,0207615

Critical t = 1,9765751

Df = 144

Sample size group 1 = 73

Sample size group 2 = 73

Total sample size = 146

Actual power = 0,8509678

**t tests** - Means: Wilcoxon-Mann-Whitney test (two groups)

**Options:** A.R.E. method

**Analysis:** A priori: Compute required sample size

**Input:** Tail(s) = Two

Parent distribution = Normal

Effect size d = 0,5

α err prob = 0,05

Power (1-β err prob) = 0,85

Allocation ratio N2/N1 = 1

**Output:** Noncentrality parameter δ = 3,0316989

Critical t = 1,9764528

Df = 145,0592

Sample size group 1 = 77

Sample size group 2 = 77

Total sample size = 154

Actual power = 0,8535097

**F tests** - ANOVA: Fixed effects, special, main effects and interactions

**Analysis:** A priori: Compute required sample size

**Input:** Effect size f = 0,25

α err prob = 0,05

Power (1-β err prob) = 0,85

Numerator df = 3

Number of groups = 4

**Output:** Noncentrality parameter λ = 12,5625000

Critical F = 2,6504417

Denominator df = 197

Total sample size = 201

Actual power = 0,8504528

Analysis 5.2: To what extent are (assistant-)psychiatrists’ conceivability toward the handling of euthanasia requests for psychiatric patients related to their socio-demographic and professional background?

- - Analysis of subsets and groups
  - Variables to be included:

| **Variable** | **Variable Type** | **Variable** | **Variable Type** | **Analyses** |
| --- | --- | --- | --- | --- |
| Sex | 2 independent groups | RefEuNL  FutureRoles [SQ001-SQ009] | Nominal | Chi kwadraat |
| Age | > 2 independent groups | RefEuNL  FutureRoles [SQ001-SQ009] | Nominal | Chi kwadraat |
| YrsAsPsych | > 2 independent groups | RefEuNL  FutureRoles [SQ001-SQ009] | Nominal | Chi kwadraat |
| SpecialEducEOL | 2 independent groups | RefEuNL  FutureRoles [SQ001-SQ009] | Nominal | Chi kwadraat |
| CompetenceEU | 2 independent groups | RefEuNL  FutureRoles [SQ001-SQ009] | Nominal | Chi kwadraat |
| WorkasPsych | 2 independent groups | RefEuNL  FutureRoles [SQ001-SQ009] | Nominal | Chi kwadraat |
| WhereWork | > 2 independent groups | RefEuNL  FutureRoles [SQ001-SQ009] | Nominal | Chi kwadraat |

G*Power: How many respondents do I need?

**χ² tests** - Goodness-of-fit tests: Contingency tables

**Analysis:** A priori: Compute required sample size

**Input:** Effect size w = 0,3

α err prob = 0,05

Power (1-β err prob) = 0,85

Df = 1

**Output:** Noncentrality parameter λ = 9,0000000

Critical χ² = 3,8414588

Total sample size = 100

Actual power = 0,8508388

**χ² tests** - Goodness-of-fit tests: Contingency tables

**Analysis:** A priori: Compute required sample size

**Input:** Effect size w = 0,3

α err prob = 0,05

Power (1-β err prob) = 0,85

Df = 2

**Output:** Noncentrality parameter λ = 10,9800000

Critical χ² = 5,9914645

Total sample size = 122

Actual power = 0,8519333

**Analysis:** A priori: Compute required sample size

**Input:** Effect size w = 0,3

α err prob = 0,05

Power (1-β err prob) = 0,85

Df = 3

**Output:** Noncentrality parameter λ = 12,3300000

Critical χ² = 7,8147279

Total sample size = 137

Actual power = 0,8509101

**χ² tests** - Goodness-of-fit tests: Contingency tables

**Analysis:** A priori: Compute required sample size

**Input:** Effect size w = 0,3

α err prob = 0,05

Power (1-β err prob) = 0,85

Df = 6

**Output:** Noncentrality parameter λ = 15,3000000

Critical χ² = 12,5915872

Total sample size = 170

Actual power = 0,8512317

Analysis 5.3: To what extent are (assistant-)psychiatrists’ experiences with psychiatric euthanasia requests and procedures related to their socio-demographic and professional background?

- - Analysis of subsets and groups.
  - Variables to be included:

`

| **Variable** | **Variable Type** | **Variable** | **Variable Type** | **Analyses** |
| --- | --- | --- | --- | --- |
| Sex | 2 independent groups | NumberEuReqNL InvolvEuNL_[0 - 8]  OftenAdv [SQ001-003]  NumberEUPerform | Ordinal Nominal  Ordinal Nominal | Mann-Whitney/K.Smirnov  Chi kwadraattoets  Mann-Whitney/K.Smirnov  Chi kwadraattoets |
| Age | > 2 independent groups | NumberEuReqNL InvolvEuNL_[0 - 8]  OftenAdv [SQ001-003]  NumberEUPerform | Ordinal Nominal  Ordinal Nominal | Kruskal-Wallis  Chi kwadraattoets  Kruskal-Wallis  Chi kwadraattoets |
| YrsAsPsych | > 2 independent groups | NumberEuReqNL InvolvEuNL_[0 - 8]  OftenAdv [SQ001-003]  NumberEUPerform | Ordinal Nominal  Ordinal Nominal | Kruskal-Wallis  Chi kwadraattoets  Kruskal-Wallis  Chi kwadraattoets |
| SpecialEducEOL | 2 independent groups | NumberEuReqNL InvolvEuNL_[0 - 8]  OftenAdv [SQ001-003]  NumberEUPerform | Ordinal Nominal  Ordinal Nominal | Mann-Whitney/K.Smirnov  Chi kwadraattoets  Mann-Whitney/K.Smirnov  Chi kwadraattoets |
| CompetenceEU | 2 independent groups | NumberEuReqNL InvolvEuNL_[0 - 8]  OftenAdv [SQ001-003]  NumberEUPerform | Ordinal Nominal  Ordinal Nominal | Mann-Whitney/K.Smirnov  Chi kwadraattoets  Mann-Whitney/K.Smirnov  Chi kwadraattoets |

G*Power: How many respondents do I need?

See above

Analysis 5.4: To what extent are (assistant-)psychiatrists’ own accounts of psychiatric euthanasia decision-making related to their socio-demographic and professional background?

- - Analysis of subsets and groups.
  - Variables to be included:

| **Variable** | **Variable Type** | **Variable** | **Variable Type** | **Analyses** |
| --- | --- | --- | --- | --- |
| Sex | 2 independent groups | ExtConsTeam[SQ001-003]  ConsCaregivers[1-11] ConsFamFriNL[1-4] | Nominal Nominal Nominal | Chi kwadraat |
| Age | > 2 independent groups | ExtConsTeam[SQ001-003]  ConsCaregivers[1-11] ConsFamFriNL[1-4] | Nominal Nominal Nominal | Chi kwadraat |
| YrsAsPsych | > 2 independent groups | ExtConsTeam[SQ001-003]  ConsCaregivers[1-11] ConsFamFriNL[1-4] | Nominal Nominal Nominal | Chi kwadraat |
| SpecialEducEOL | 2 independent groups | ExtConsTeam[SQ001-003]  ConsCaregivers[1-11] ConsFamFriNL[1-4] | Nominal Nominal Nominal | Chi kwadraat |
| CompetenceEU | 2 independent groups | ExtConsTeam[SQ001-003]  ConsCaregivers[1-11] ConsFamFriNL[1-4] | Nominal Nominal Nominal | Chi kwadraat |
| WorkasPsych | 2 independent groups | ExtConsTeam[SQ001-003]  ConsCaregivers[1-11] ConsFamFriNL[1-4] | Nominal Nominal Nominal | Chi kwadraat |
| WhereWork | > 2 independent groups | ExtConsTeam[SQ001-003]  ConsCaregivers[1-11] ConsFamFriNL[1-4] | Nominal Nominal Nominal | Chi kwadraat |

G*Power: How many respondents do I need?

See above

# Syntax

## Data import Online Survey: in Dutch

************************************************************************************************************************************************************************************************************************************************

*$Rev: 121017 $ all 2.

SET UNICODE=ON.

SHOW LOCALE.

PRESERVE LOCALE.

SET LOCALE='en_UK'.

GET DATA

/TYPE=TXT

/FILE='survey_**survey number**_SPSS_data_file.dat'

/DELCASE=LINE

/DELIMITERS=","

/QUALIFIER="'"

/ARRANGEMENT=DELIMITED

/FIRSTCASE=1

/IMPORTCASE=ALL

/VARIABLES=

V1 A100

V2 A100

V3 A100

V4 A100

V5 A100

V6 A100

V7 A100

V8 A100

V9 A100

V10 F7

V11 A16

V12 DATETIME23.2

V13 F7

V14 A19

V15 F1

V16 F1

V17 F1

V18 F1

V19 F1

V20 F1

V21 F1

V22 F1

V23 F1

V24 A38

V25 F1

V26 F1

V27 F1

V28 F1

V29 F1

V30 F1

V31 F1

V32 F1

V33 F1

V34 F1

V35 F1

V36 F1

V37 F1

V38 F1

V39 F1

V40 F1

V41 F1

V42 F1

V43 F1

V44 A1173

V45 F1

V46 F1

V47 F1

V48 F1

V49 F1

V50 F1

V51 F1

V52 F1

V53 F1

V54 A99

V55 F1

V56 A2

V57 A2

V58 A2

V59 F1

V60 F1

V61 F1

V62 F1

V63 F1

V64 F1

V65 F1

V66 F1

V67 F1

V68 F1

V69 A73

V70 F1

V71 F1

V72 F1

V73 A21

V74 A563

V75 A1574

V76 F1

V77 F1

V78 F1

V79 F1

V80 F1

V81 F1

V82 F1

V83 F12.11

V84 F13.12

V85 F12.11

V86 F1.0

V87 F12.11

V88 F13.12

V89 F1

V90 F1

V91 F1

V92 F1

V93 F1

V94 F1

V95 A100

V96 F1

V97 A105

V98 F1

V99 A35

V100 F1

V101 A69

V102 F1

V103 F1

V104 F1

V105 F1

V106 F1

V107 F1

V108 F1

V109 F1

V110 F1

V111 F1

V112 F1

V113 F1

V114 F1

V115 F1

V116 F1

V117 A118

V118 F1

V119 F1

V120 F1

V121 F1

V122 F1

V123 F1

V124 F1

V125 F1

V126 F1

V127 F1

V128 F1

V129 F1

V130 F1

V131 A51

V132 F1

V133 F1

V134 F1

V135 F1

V136 F1

V137 F1

V138 F1

V139 F1

V140 F1

V141 F1

V142 F1

V143 F1

V144 F1

V145 F1

V146 F1

V147 F1

V148 A42

V149 F1

V150 F1

V151 F1

V152 F1

V153 F1

V154 F1

V155 F1

V156 F1

V157 F1

V158 F1

V159 F1

V160 F1

V161 F1

V162 F1

V163 F1

V164 F1

V165 F1

V166 A2

V167 A2

V168 F1

V169 A171

V170 F1

V171 F1

V172 F1

V173 F1

V174 F1

V175 F1

V176 F1

V177 F1

V178 F1

V179 F1

V180 F1

V181 A150

V182 A341

V183 F1

V184 F1

V185 F1

V186 F1

V187 F1

V188 F1

V189 A91.

CACHE.

EXECUTE.

*Define Variable Properties.

VARIABLE LABELS V1 "Voornaam".

VARIABLE LABELS V2 "Achternaam".

VARIABLE LABELS V3 "E-mailadres".

VARIABLE LABELS V4 "E-mail status".

VARIABLE LABELS V5 "Taalcode".

VARIABLE LABELS V6 "Verzenddatum uitnodiging".

VARIABLE LABELS V7 "Datum verzenden laatste herinnering".

VARIABLE LABELS V8 "Aantal verzonden herinneringen".

VARIABLE LABELS V9 "Aantal keer nog te gebruiken".

VARIABLE LABELS V10 "id".

VARIABLE LABELS V11 "token".

VARIABLE LABELS V12 "submitdate".

VARIABLE LABELS V13 "lastpage".

VARIABLE LABELS V14 "startlanguage".

VARIABLE LABELS V15 "Alvorens van start te gaan, willen we u graag inlichten over waarom en hoe wij uw anonimiteit en privacy kunnen en zullen garanderen. Deze informatie vindt u onderaan deze pagina. Klik hieronder op  'ja' indien u instemt met dez"+

"e garanties en aan het onderzoek wil deelnemen. Klik hieronder op 'nee' indien u niet aan dit onderzoek wil deelnemen.".

VARIABLE LABELS V16 "Was u de voorbije 12 maanden werkzaam als (assistent-)psychiater van volwassen patiënten?  ".

VARIABLE LABELS V17 "[In een privé (groeps)praktijk] Was u in deze periode als psychiater werkzaam:  ".

VARIABLE LABELS V18 "[In een ziekenhuis (PAAZ/PZ)] Was u in deze periode als psychiater werkzaam:  ".

VARIABLE LABELS V19 "[In een Centrum voor Geestelijke Gezondheidszorg (CGG)] Was u in deze periode als psychiater werkzaam:  ".

VARIABLE LABELS V20 "[In een Psychiatrisch Verzorgingstehuis (PVT)] Was u in deze periode als psychiater werkzaam:  ".

VARIABLE LABELS V21 "[Psychiatrische Zorg Thuissituatie (PZT)] Was u in deze periode als psychiater werkzaam:  ".

VARIABLE LABELS V22 "[Initiatief Beschut wonen] Was u in deze periode als psychiater werkzaam:  ".

VARIABLE LABELS V23 "[Anders] Was u in deze periode als psychiater werkzaam:  ".

VARIABLE LABELS V24 "Waar was u werkzaam?".

VARIABLE LABELS V25 "Sinds hoeveel jaren bent u als psychiater werkzaam (geweest), inclusief als assistent?".

VARIABLE LABELS V26 "Heeft u een gespecialiseerde opleiding gevolgd rond palliatieve en/of andere levenseindezorg?  ".

VARIABLE LABELS V27 "Voelt u zich voldoende competent om als psychiater betrokken te worden bij een euthanasieprocedure?".

VARIABLE LABELS V28 "Wat is uw leeftijd?".

VARIABLE LABELS V29 "Wat is uw geslacht?".

VARIABLE LABELS V30 "[1. Euthanasie zou enkel voor terminaal zieke patiënten wettelijk mogelijk moeten zijn.] Geef aan in hoeverre u het eens of oneens bent met de volgende 13 stellingen. Het gaat hierbij telkens om uw persoonlijke mening, niet om "+

"wat al dan niet wettelijk toegestaan is. Er zijn dus geen juiste of foute antwoorden. Noot: bij stellingen over psychiatrische patiënten, gaat het louter over patiënten met een psychiatrische ziektebeeld als onderliggende aando"+

"ening. (Indien nodig, vindt u de begrippenlijst onderaan).    ".

VARIABLE LABELS V31 "[2. Euthanasie zou voor niet-terminale patiënten wettelijk mogelijk moeten zijn, maar enkel als er sprake is van een somatische aandoening.] Geef aan in hoeverre u het eens of oneens bent met de volgende 13 stellingen. Het gaat"+

" hierbij telkens om uw persoonlijke mening, niet om wat al dan niet wettelijk toegestaan is. Er zijn dus geen juiste of foute antwoorden. Noot: bij stellingen over psychiatrische patiënten, gaat het louter over patiënten met ee"+

"n psychiatrische ziektebeeld als onderliggende aandoening. (Indien nodig, vindt u de begrippenlijst onderaan).    ".

VARIABLE LABELS V32 "[3. Euthanasie zou voor patiënten met een psychiatrische aandoening wettelijk mogelijk moeten blijven.] Geef aan in hoeverre u het eens of oneens bent met de volgende 13 stellingen. Het gaat hierbij telkens om uw persoonlijke m"+

"ening, niet om wat al dan niet wettelijk toegestaan is. Er zijn dus geen juiste of foute antwoorden. Noot: bij stellingen over psychiatrische patiënten, gaat het louter over patiënten met een psychiatrische ziektebeeld als onde"+

"rliggende aandoening. (Indien nodig, vindt u de begrippenlijst onderaan).    ".

VARIABLE LABELS V33 "[4. Euthanasie bij psychiatrische patiënten is verenigbaar met een psychiatrische hulpverleningsrelatie.] Geef aan in hoeverre u het eens of oneens bent met de volgende 13 stellingen. Het gaat hierbij telkens om uw persoonlijke"+

" mening, niet om wat al dan niet wettelijk toegestaan is. Er zijn dus geen juiste of foute antwoorden. Noot: bij stellingen over psychiatrische patiënten, gaat het louter over patiënten met een psychiatrische ziektebeeld als on"+

"derliggende aandoening. (Indien nodig, vindt u de begrippenlijst onderaan).    ".

VARIABLE LABELS V34 "[5. Bij psychiatrische patiënten is medisch begeleide zelfdoding (patiënt neemt zelf de letale dosis in, in aanwezigheid en onder begeleiding van de arts) voor mij meer aanvaardbaar dan euthanasie (arts dient letale dosis toe aa"+

"n de patiënt).] Geef aan in hoeverre u het eens of oneens bent met de volgende 13 stellingen. Het gaat hierbij telkens om uw persoonlijke mening, niet om wat al dan niet wettelijk toegestaan is. Er zijn dus geen juiste of foute"+

" antwoorden. Noot: bij stellingen over psychiatrische patiënten, gaat het louter over patiënten met een psychiatrische ziektebeeld als onderliggende aandoening. (Indien nodig, vindt u de begrippenlijst onderaan).    ".

VARIABLE LABELS V35 "[6. Een psychiatrische patiënt kan zich in een medisch uitzichtloze toestand bevinden.] Geef aan in hoeverre u het eens of oneens bent met de volgende 13 stellingen. Het gaat hierbij telkens om uw persoonlijke mening, niet om w"+

"at al dan niet wettelijk toegestaan is. Er zijn dus geen juiste of foute antwoorden. Noot: bij stellingen over psychiatrische patiënten, gaat het louter over patiënten met een psychiatrische ziektebeeld als onderliggende aandoe"+

"ning. (Indien nodig, vindt u de begrippenlijst onderaan).    ".

VARIABLE LABELS V36 "[7. Een psychiatrische patiënt kan ondraaglijk lijden.] Geef aan in hoeverre u het eens of oneens bent met de volgende 13 stellingen. Het gaat hierbij telkens om uw persoonlijke mening, niet om wat al dan niet wettelijk toegest"+

"aan is. Er zijn dus geen juiste of foute antwoorden. Noot: bij stellingen over psychiatrische patiënten, gaat het louter over patiënten met een psychiatrische ziektebeeld als onderliggende aandoening. (Indien nodig, vindt u de "+

"begrippenlijst onderaan).    ".

VARIABLE LABELS V37 "[8. Een redelijk behandelperspectief kan ontbreken bij een psychiatrische patiënt.] Geef aan in hoeverre u het eens of oneens bent met de volgende 13 stellingen. Het gaat hierbij telkens om uw persoonlijke mening, niet om wat a"+

"l dan niet wettelijk toegestaan is. Er zijn dus geen juiste of foute antwoorden. Noot: bij stellingen over psychiatrische patiënten, gaat het louter over patiënten met een psychiatrische ziektebeeld als onderliggende aandoening"+

". (Indien nodig, vindt u de begrippenlijst onderaan).    ".

VARIABLE LABELS V38 "[9. Euthanasie is een aanvaardbaar alternatief om suïcide te voorkomen.] Geef aan in hoeverre u het eens of oneens bent met de volgende 13 stellingen. Het gaat hierbij telkens om uw persoonlijke mening, niet om wat al dan niet "+

"wettelijk toegestaan is. Er zijn dus geen juiste of foute antwoorden. Noot: bij stellingen over psychiatrische patiënten, gaat het louter over patiënten met een psychiatrische ziektebeeld als onderliggende aandoening. (Indien n"+

"odig, vindt u de begrippenlijst onderaan).    ".

VARIABLE LABELS V39 "[10. Bij de uitklaring van een euthanasieverzoek moet rekening gehouden worden met mogelijke effectieve therapeutische ontwikkelingen in de toekomst.] Geef aan in hoeverre u het eens of oneens bent met de volgende 13 stellingen"+

". Het gaat hierbij telkens om uw persoonlijke mening, niet om wat al dan niet wettelijk toegestaan is. Er zijn dus geen juiste of foute antwoorden. Noot: bij stellingen over psychiatrische patiënten, gaat het louter over patiën"+

"ten met een psychiatrische ziektebeeld als onderliggende aandoening. (Indien nodig, vindt u de begrippenlijst onderaan).    ".

VARIABLE LABELS V40 "[11. Bij de uitklaring van een euthanasieverzoek dient de focus niet louter op de medische, maar op de volledige leefsituatie van de patiënt gelegd te worden.] Geef aan in hoeverre u het eens of oneens bent met de volgende 13 s"+

"tellingen. Het gaat hierbij telkens om uw persoonlijke mening, niet om wat al dan niet wettelijk toegestaan is. Er zijn dus geen juiste of foute antwoorden. Noot: bij stellingen over psychiatrische patiënten, gaat het louter ov"+

"er patiënten met een psychiatrische ziektebeeld als onderliggende aandoening. (Indien nodig, vindt u de begrippenlijst onderaan).    ".

VARIABLE LABELS V41 "[12. Een doodswens van een psychiatrische patiënt kan weloverwogen zijn en niet louter een symptoom van een onderliggende pathologie.] Geef aan in hoeverre u het eens of oneens bent met de volgende 13 stellingen. Het gaat hier"+

"bij telkens om uw persoonlijke mening, niet om wat al dan niet wettelijk toegestaan is. Er zijn dus geen juiste of foute antwoorden. Noot: bij stellingen over psychiatrische patiënten, gaat het louter over patiënten met een psy"+

"chiatrische ziektebeeld als onderliggende aandoening. (Indien nodig, vindt u de begrippenlijst onderaan).    ".

VARIABLE LABELS V42 "[13. In bepaalde gevallen wordt er te lichtzinnig omgegaan met de mogelijkheid van euthanasie voor psychiatrische patiënten.] Geef aan in hoeverre u het eens of oneens bent met de volgende 13 stellingen. Het gaat hierbij telken"+

"s om uw persoonlijke mening, niet om wat al dan niet wettelijk toegestaan is. Er zijn dus geen juiste of foute antwoorden. Noot: bij stellingen over psychiatrische patiënten, gaat het louter over patiënten met een psychiatrisch"+

"e ziektebeeld als onderliggende aandoening. (Indien nodig, vindt u de begrippenlijst onderaan).    ".

VARIABLE LABELS V43 "Heeft u als behandelend arts van een psychiatrische patiënt ooit geweigerd om actief betrokken te zijn bij de uitklaring van diens uitdrukkelijk euthanasieverzoek?".

VARIABLE LABELS V44 "Om welke reden(en) heeft u hiertoe besloten?".

VARIABLE LABELS V45 "[Nee, ik werd hier nooit mee geconfronteerd] Werd u tijdens uw loopbaan als psychiater ooit betrokken bij een uitdrukkelijk euthanasieverzoek van een volwassen patiënt die hoofdzakelijk leed aan 1 of meer psychiatrische aandoeni"+

"ng(en)? ".

VARIABLE LABELS V46 "[Ja, als behandelend arts, die de eigen patiënt doorverwees naar een collega-arts i.f.v. een euthanasieprocedure] Werd u tijdens uw loopbaan als psychiater ooit betrokken bij een uitdrukkelijk euthanasieverzoek van een volwassen"+

" patiënt die hoofdzakelijk leed aan 1 of meer psychiatrische aandoening(en)? ".

VARIABLE LABELS V47 "[Ja, als behandelend arts, die de uitklaring van het euthanasieverzoek van een eigen patiënt zelf opnam] Werd u tijdens uw loopbaan als psychiater ooit betrokken bij een uitdrukkelijk euthanasieverzoek van een volwassen patiënt "+

"die hoofdzakelijk leed aan 1 of meer psychiatrische aandoening(en)? ".

VARIABLE LABELS V48 "[Ja, als arts die de uitklaring van het euthanasieverzoek van een patiënt van een collega-arts opnam] Werd u tijdens uw loopbaan als psychiater ooit betrokken bij een uitdrukkelijk euthanasieverzoek van een volwassen patiënt die"+

" hoofdzakelijk leed aan 1 of meer psychiatrische aandoening(en)? ".

VARIABLE LABELS V49 "[Ja, als preliminair adviserend arts over een deelaspect (bv. uitsluiten acute depressie, beoordelen wilsbekwaamheid)] Werd u tijdens uw loopbaan als psychiater ooit betrokken bij een uitdrukkelijk euthanasieverzoek van een volw"+

"assen patiënt die hoofdzakelijk leed aan 1 of meer psychiatrische aandoening(en)? ".

VARIABLE LABELS V50 "[Ja, als procedureel adviserend arts in het kader van een wettelijk vereist vereist 1ste of 2de euthanasie-advies ] Werd u tijdens uw loopbaan als psychiater ooit betrokken bij een uitdrukkelijk euthanasieverzoek van een volwass"+

"en patiënt die hoofdzakelijk leed aan 1 of meer psychiatrische aandoening(en)? ".

VARIABLE LABELS V51 "[Ja, als uitvoerend arts die euthanasie bij een eigen patiënt uitvoerde, hierbij assisteerde of aanwezig was] Werd u tijdens uw loopbaan als psychiater ooit betrokken bij een uitdrukkelijk euthanasieverzoek van een volwassen pat"+

"iënt die hoofdzakelijk leed aan 1 of meer psychiatrische aandoening(en)? ".

VARIABLE LABELS V52 "[Ja, als uitvoerend arts die euthanasie bij een patiënt van een collega-arts uitvoerde/assisteerde of erbij aanwezig was] Werd u tijdens uw loopbaan als psychiater ooit betrokken bij een uitdrukkelijk euthanasieverzoek van een v"+

"olwassen patiënt die hoofdzakelijk leed aan 1 of meer psychiatrische aandoening(en)? ".

VARIABLE LABELS V53 "[Ja, in een andere rol] Werd u tijdens uw loopbaan als psychiater ooit betrokken bij een uitdrukkelijk euthanasieverzoek van een volwassen patiënt die hoofdzakelijk leed aan 1 of meer psychiatrische aandoening(en)? ".

VARIABLE LABELS V54 "In welke andere rol?".

VARIABLE LABELS V55 "Bij hoeveel euthanasieverzoeken van dergelijke patiënten werd u de afgelopen 12 maanden (in eender welke professionele rol) betrokken?".

VARIABLE LABELS V56 "[Positief advies] Hoe vaak heeft u de voorbije 12 maanden een positief dan wel negatief advies gegeven of een advies geweigerd?".

VARIABLE LABELS V57 "[Negatief advies] Hoe vaak heeft u de voorbije 12 maanden een positief dan wel negatief advies gegeven of een advies geweigerd?".

VARIABLE LABELS V58 "[Advies geweigerd] Hoe vaak heeft u de voorbije 12 maanden een positief dan wel negatief advies gegeven of een advies geweigerd?".

VARIABLE LABELS V59 "Bij hoeveel psychiatrische patiënten was u de afgelopen 5 jaar als uitvoerend arts betrokken?".

VARIABLE LABELS V60 "[Nee, in geen enkele rol] Acht u het denkbaar dat u in de toekomst één of meer van volgende rollen opneemt in het kader van concrete euthanasieverzoeken van volwassen patiënten met (een) psychiatrische aandoening(en). ".

VARIABLE LABELS V61 "[Ja, als behandelend arts, die de eigen patiënt doorverwijst naar een collega-arts voor de verdere uitklaring/advies] Acht u het denkbaar dat u in de toekomst één of meer van volgende rollen opneemt in het kader van concrete eu"+

"thanasieverzoeken van volwassen patiënten met (een) psychiatrische aandoening(en). ".

VARIABLE LABELS V62 "[Ja, als behandelend arts, die de uitklaring van het euthanasieverzoek van een eigen patiënt zelf opneemt ] Acht u het denkbaar dat u in de toekomst één of meer van volgende rollen opneemt in het kader van concrete euthanasiev"+

"erzoeken van volwassen patiënten met (een) psychiatrische aandoening(en). ".

VARIABLE LABELS V63 "[Ja, als arts die de uitklaring van het euthanasieverzoek van een patiënt van een collega-arts opneemt] Acht u het denkbaar dat u in de toekomst één of meer van volgende rollen opneemt in het kader van concrete euthanasieverzoe"+

"ken van volwassen patiënten met (een) psychiatrische aandoening(en). ".

VARIABLE LABELS V64 "[Ja, als preliminair adviserend arts over een deelaspect (bv. uitsluiten acute depressie, beoordelen wilsbekwaamheid)] Acht u het denkbaar dat u in de toekomst één of meer van volgende rollen opneemt in het kader van concrete e"+

"uthanasieverzoeken van volwassen patiënten met (een) psychiatrische aandoening(en). ".

VARIABLE LABELS V65 "[Ja, als procedureel adviserend arts in het kader van een wettelijk vereist 1ste of 2de euthanasie-advies] Acht u het denkbaar dat u in de toekomst één of meer van volgende rollen opneemt in het kader van concrete euthanasiever"+

"zoeken van volwassen patiënten met (een) psychiatrische aandoening(en). ".

VARIABLE LABELS V66 "[Ja, als uitvoerend arts, die de euthanasie bij een eigen patiënt uitvoert, hierbij assisteert of aanwezig is] Acht u het denkbaar dat u in de toekomst één of meer van volgende rollen opneemt in het kader van concrete euthanasi"+

"everzoeken van volwassen patiënten met (een) psychiatrische aandoening(en). ".

VARIABLE LABELS V67 "[Ja, als uitvoerend arts, die de euthanasie bij een patiënt van een collega-arts uitvoert, hierbij assisteert of aanwezig is] Acht u het denkbaar dat u in de toekomst één of meer van volgende rollen opneemt in het kader van con"+

"crete euthanasieverzoeken van volwassen patiënten met (een) psychiatrische aandoening(en). ".

VARIABLE LABELS V68 "[Ja, in een andere rol] Acht u het denkbaar dat u in de toekomst één of meer van volgende rollen opneemt in het kader van concrete euthanasieverzoeken van volwassen patiënten met (een) psychiatrische aandoening(en). ".

VARIABLE LABELS V69 "In welke andere rol?".

VARIABLE LABELS V70 "[Nee, en ik zou dat ook nooit doen ] Heeft u ooit een extern consultatieteam, gespecialiseerd in de uitklaring van euthanasieverzoeken (bv. ULteam, Vonkel) betrokken bij de euthanasieprocedure van een psychiatrische patiënt?".

VARIABLE LABELS V71 "[Nee, maar het is wel denkbaar dat ik dat ooit doe ] Heeft u ooit een extern consultatieteam, gespecialiseerd in de uitklaring van euthanasieverzoeken (bv. ULteam, Vonkel) betrokken bij de euthanasieprocedure van een psychiatris"+

"che patiënt?".

VARIABLE LABELS V72 "[Ja] Heeft u ooit een extern consultatieteam, gespecialiseerd in de uitklaring van euthanasieverzoeken (bv. ULteam, Vonkel) betrokken bij de euthanasieprocedure van een psychiatrische patiënt?".

VARIABLE LABELS V73 "Op welk extern consultatieteam heeft u beroep gedaan?".

VARIABLE LABELS V74 "Om welke reden(en) zou u al dan niet beroep op een extern consultatieteam doen?".

VARIABLE LABELS V75 "Wil u nog iets verduidelijken of toelichten?".

VARIABLE LABELS V76 "Heeft u de voorbije 12 maanden ervaring gehad met een volledig afgeronde euthanasieprocedure (ongeacht de einduitkomst) van een patiënt met een psychiatrische aandoening tijdens de voorbije 12 maanden?".

VARIABLE LABELS V77 "[Behandelaar van de patiënt en diens psychopathologie] Wat was uw specifieke functie? ".

VARIABLE LABELS V78 "[Uitklaring euthanasieverzoek van een eigen patiënt] Wat was uw specifieke functie? ".

VARIABLE LABELS V79 "[Uitklaring euthanasieverzoek van een patiënt van een collega-arts ] Wat was uw specifieke functie? ".

VARIABLE LABELS V80 "[Preliminair adviserend arts ] Wat was uw specifieke functie? ".

VARIABLE LABELS V81 "[Procedureel adviserend arts] Wat was uw specifieke functie? ".

VARIABLE LABELS V82 "[Uitvoerend arts ] Wat was uw specifieke functie? ".

VARIABLE LABELS V83 "[Weken] Hoeveel tijd nam de besluitvorming in beslag vanaf het euthanasieverzoek tot de eindbeslissing? (vul aantal weken/maanden/jaren in)".

VARIABLE LABELS V84 "[Maanden] Hoeveel tijd nam de besluitvorming in beslag vanaf het euthanasieverzoek tot de eindbeslissing? (vul aantal weken/maanden/jaren in)".

VARIABLE LABELS V85 "[Jaren] Hoeveel tijd nam de besluitvorming in beslag vanaf het euthanasieverzoek tot de eindbeslissing? (vul aantal weken/maanden/jaren in)".

VARIABLE LABELS V86 "[Weken] Hoe lang was de patiënt reeds in behandeling voor hij/zij u consulteerde met een euthanasieverzoek? (vul aantal weken/maanden/jaren in)".

VARIABLE LABELS V87 "[Maanden] Hoe lang was de patiënt reeds in behandeling voor hij/zij u consulteerde met een euthanasieverzoek? (vul aantal weken/maanden/jaren in)".

VARIABLE LABELS V88 "[Jaren] Hoe lang was de patiënt reeds in behandeling voor hij/zij u consulteerde met een euthanasieverzoek? (vul aantal weken/maanden/jaren in)".

VARIABLE LABELS V89 "[Nee, patiënt volgende geen behandeling ] Volgde de patiënt psychotherapeutische behandeling(en) op het moment dat hij/zij u voor het eerst consulteerde in het kader van euthanasie? ".

VARIABLE LABELS V90 "[Ja, psychofarmaca ] Volgde de patiënt psychotherapeutische behandeling(en) op het moment dat hij/zij u voor het eerst consulteerde in het kader van euthanasie? ".

VARIABLE LABELS V91 "[Ja, andere farmaca] Volgde de patiënt psychotherapeutische behandeling(en) op het moment dat hij/zij u voor het eerst consulteerde in het kader van euthanasie? ".

VARIABLE LABELS V92 "[Ja, gesprekstherapie] Volgde de patiënt psychotherapeutische behandeling(en) op het moment dat hij/zij u voor het eerst consulteerde in het kader van euthanasie? ".

VARIABLE LABELS V93 "[Ja, neurochirurgische behandeling] Volgde de patiënt psychotherapeutische behandeling(en) op het moment dat hij/zij u voor het eerst consulteerde in het kader van euthanasie? ".

VARIABLE LABELS V94 "[Ja, andere interventies] Volgde de patiënt psychotherapeutische behandeling(en) op het moment dat hij/zij u voor het eerst consulteerde in het kader van euthanasie? ".

VARIABLE LABELS V95 "Welke andere interventies?".

VARIABLE LABELS V96 "[Klinische stoornis, namelijk] Wat was de belangrijkste pathologie van de patiënt? ".

VARIABLE LABELS V97 "[Opmerking] Wat was de belangrijkste pathologie van de patiënt? ".

VARIABLE LABELS V98 "[Persoonlijkheidsstoornis, namelijk] Wat was de belangrijkste pathologie van de patiënt? ".

VARIABLE LABELS V99 "[Opmerking] Wat was de belangrijkste pathologie van de patiënt? ".

VARIABLE LABELS V100 "[Somatische aandoeningen, namelijk] Wat was de belangrijkste pathologie van de patiënt? ".

VARIABLE LABELS V101 "[Opmerking] Wat was de belangrijkste pathologie van de patiënt? ".

VARIABLE LABELS V102 "[Depressieve gevoelens] Wat waren de redenen van de patiënt om euthanasie te vragen? ".

VARIABLE LABELS V103 "[Vastlopen op meerdere levensgebieden (werk/relatie/…)] Wat waren de redenen van de patiënt om euthanasie te vragen? ".

VARIABLE LABELS V104 "[Geen perspectief op verbetering] Wat waren de redenen van de patiënt om euthanasie te vragen? ".

VARIABLE LABELS V105 "[Geen doel (meer) in het leven] Wat waren de redenen van de patiënt om euthanasie te vragen? ".

VARIABLE LABELS V106 "[Eenzaamheid] Wat waren de redenen van de patiënt om euthanasie te vragen? ".

VARIABLE LABELS V107 "[Omgeving niet (langer) tot last willen zijn ] Wat waren de redenen van de patiënt om euthanasie te vragen? ".

VARIABLE LABELS V108 "[Existentieel lijden (lijden aan het leven, zinloosheid)] Wat waren de redenen van de patiënt om euthanasie te vragen? ".

VARIABLE LABELS V109 "[Gestage deterioratie ] Wat waren de redenen van de patiënt om euthanasie te vragen? ".

VARIABLE LABELS V110 "[Angst voor suïcide ] Wat waren de redenen van de patiënt om euthanasie te vragen? ".

VARIABLE LABELS V111 "[Invaliditeit/immobiliteit] Wat waren de redenen van de patiënt om euthanasie te vragen? ".

VARIABLE LABELS V112 "[Verlies van autonomie, regie over het eigen leven] Wat waren de redenen van de patiënt om euthanasie te vragen? ".

VARIABLE LABELS V113 "[Verlies van waardigheid] Wat waren de redenen van de patiënt om euthanasie te vragen? ".

VARIABLE LABELS V114 "[Totale uitputting ] Wat waren de redenen van de patiënt om euthanasie te vragen? ".

VARIABLE LABELS V115 "[Geen levenskwaliteit, enkel ‘overleven’ ] Wat waren de redenen van de patiënt om euthanasie te vragen? ".

VARIABLE LABELS V116 "[Andere] Wat waren de redenen van de patiënt om euthanasie te vragen? ".

VARIABLE LABELS V117 "Wat waren volgens u de 2 voornaamste redenen van de patiënt om euthanasie te vragen?".

VARIABLE LABELS V118 "[Neen] Heeft u één of meerdere artsen/hulpverleners/instanties geconsulteerd tijdens de besluitvorming?".

VARIABLE LABELS V119 "[Ja, de huisarts(en) van de patiënt ] Heeft u één of meerdere artsen/hulpverleners/instanties geconsulteerd tijdens de besluitvorming?".

VARIABLE LABELS V120 "[Ja, andere arts(en) van de patiënt] Heeft u één of meerdere artsen/hulpverleners/instanties geconsulteerd tijdens de besluitvorming?".

VARIABLE LABELS V121 "[Ja, onafhankelijke collega-psychiater(s)] Heeft u één of meerdere artsen/hulpverleners/instanties geconsulteerd tijdens de besluitvorming?".

VARIABLE LABELS V122 "[Ja, onafhankelijke LEIF-arts(en)] Heeft u één of meerdere artsen/hulpverleners/instanties geconsulteerd tijdens de besluitvorming?".

VARIABLE LABELS V123 "[Ja, onafhankelijke arts(en) van levenseinde consultatieteam ] Heeft u één of meerdere artsen/hulpverleners/instanties geconsulteerd tijdens de besluitvorming?".

VARIABLE LABELS V124 "[Ja, verpleegkundige(n)] Heeft u één of meerdere artsen/hulpverleners/instanties geconsulteerd tijdens de besluitvorming?".

VARIABLE LABELS V125 "[Ja, ethische commissie] Heeft u één of meerdere artsen/hulpverleners/instanties geconsulteerd tijdens de besluitvorming?".

VARIABLE LABELS V126 "[Ja, andere interne adviescommissie] Heeft u één of meerdere artsen/hulpverleners/instanties geconsulteerd tijdens de besluitvorming?".

VARIABLE LABELS V127 "[Ja, psycholo(o)g(en)] Heeft u één of meerdere artsen/hulpverleners/instanties geconsulteerd tijdens de besluitvorming?".

VARIABLE LABELS V128 "[Ja, palliatief zorgteam] Heeft u één of meerdere artsen/hulpverleners/instanties geconsulteerd tijdens de besluitvorming?".

VARIABLE LABELS V129 "[Ja, een (psycho-)sociale dienst ] Heeft u één of meerdere artsen/hulpverleners/instanties geconsulteerd tijdens de besluitvorming?".

VARIABLE LABELS V130 "[Ja, andere(n)] Heeft u één of meerdere artsen/hulpverleners/instanties geconsulteerd tijdens de besluitvorming?".

VARIABLE LABELS V131 "Wie of welke instanties heeft u daarnaast nog geconsulteerd?".

VARIABLE LABELS V132 "[Neen, geen familie of vrienden betrokken ] Heeft u overleg gehad met familie en/of vrienden van de patiënt? ".

VARIABLE LABELS V133 "[Neen, patiënt had geen familie of vrienden ] Heeft u overleg gehad met familie en/of vrienden van de patiënt? ".

VARIABLE LABELS V134 "[Ja, tijdens euthanasieprocedure] Heeft u overleg gehad met familie en/of vrienden van de patiënt? ".

VARIABLE LABELS V135 "[Ja, na afronding van de euthanasieprocedure] Heeft u overleg gehad met familie en/of vrienden van de patiënt? ".

VARIABLE LABELS V136 "[Mentale competentie (wilsbekwaamheid)] Was er bij deze patiënt naar uw oordeel sprake van…".

VARIABLE LABELS V137 "[Ongeneeslijkheid van de aandoening] Was er bij deze patiënt naar uw oordeel sprake van…".

VARIABLE LABELS V138 "[Ondraaglijkheid van het lijden] Was er bij deze patiënt naar uw oordeel sprake van…".

VARIABLE LABELS V139 "[Uitzichtloosheid van de medische conditie] Was er bij deze patiënt naar uw oordeel sprake van…".

VARIABLE LABELS V140 "[Ontbreken redelijk therapeutisch perspectief] Was er bij deze patiënt naar uw oordeel sprake van…".

VARIABLE LABELS V141 "[Vrijwillig, duurzaam en weloverwogen verzoek] Was er bij deze patiënt naar uw oordeel sprake van…".

VARIABLE LABELS V142 "[Mentale competentie (wilsbekwaamheid)] Hoeveel moeilijkheden heeft u ervaren om onderstaande criteria te kunnen beoordelen?".

VARIABLE LABELS V143 "[Ongeneeslijkheid van de aandoening] Hoeveel moeilijkheden heeft u ervaren om onderstaande criteria te kunnen beoordelen?".

VARIABLE LABELS V144 "[Ondraaglijkheid van het lijden] Hoeveel moeilijkheden heeft u ervaren om onderstaande criteria te kunnen beoordelen?".

VARIABLE LABELS V145 "[Uitzichtloosheid van de medische conditie] Hoeveel moeilijkheden heeft u ervaren om onderstaande criteria te kunnen beoordelen?".

VARIABLE LABELS V146 "[Ontbreken redelijk therapeutisch perspectief] Hoeveel moeilijkheden heeft u ervaren om onderstaande criteria te kunnen beoordelen?".

VARIABLE LABELS V147 "[Vrijwillig, duurzaam en weloverwogen verzoek] Hoeveel moeilijkheden heeft u ervaren om onderstaande criteria te kunnen beoordelen?".

VARIABLE LABELS V148 "Zijn er nog andere criteria die u moeilijk te beoordelen vond?".

VARIABLE LABELS V149 "[Patiënt die onder druk van derden om euthanasie verzoekt] Werd u tijdens deze euthanasieprocedure geconfronteerd met onderstaande vormen van druk?".

VARIABLE LABELS V150 "[Druk van patiënt om tot euthanasie te besluiten (advies/uitvoering)] Werd u tijdens deze euthanasieprocedure geconfronteerd met onderstaande vormen van druk?".

VARIABLE LABELS V151 "[Druk van naasten om tot euthanasie te besluiten (advies/uitvoering)] Werd u tijdens deze euthanasieprocedure geconfronteerd met onderstaande vormen van druk?".

VARIABLE LABELS V152 "[Druk van naasten om het euthanasieverzoek af te wijzen] Werd u tijdens deze euthanasieprocedure geconfronteerd met onderstaande vormen van druk?".

VARIABLE LABELS V153 "[Druk van collega’s om het euthanasieverzoek af te wijzen] Werd u tijdens deze euthanasieprocedure geconfronteerd met onderstaande vormen van druk?".

VARIABLE LABELS V154 "[Druk van collega’s om tot euthanasie te besluiten (advies/uitvoering)] Werd u tijdens deze euthanasieprocedure geconfronteerd met onderstaande vormen van druk?".

VARIABLE LABELS V155 "[Druk van zorginstelling om het euthanasieverzoek af te wijzen] Werd u tijdens deze euthanasieprocedure geconfronteerd met onderstaande vormen van druk?".

VARIABLE LABELS V156 "[Druk van zorginstelling om tot euthanasie te besluiten (advies/uitvoering)] Werd u tijdens deze euthanasieprocedure geconfronteerd met onderstaande vormen van druk?".

VARIABLE LABELS V157 "[Hoge emotionele belasting voor uzelf] Heeft u tijdens deze euthanasieprocedure de volgende ervaringen beleefd?".

VARIABLE LABELS V158 "[Nieuwe therapeutische kansen bij de patiënt] Heeft u tijdens deze euthanasieprocedure de volgende ervaringen beleefd?".

VARIABLE LABELS V159 "[Verlaagd risico op suïcide bij de patiënt] Heeft u tijdens deze euthanasieprocedure de volgende ervaringen beleefd?".

VARIABLE LABELS V160 "[Herstel relaties tussen patiënt en diens naasten] Heeft u tijdens deze euthanasieprocedure de volgende ervaringen beleefd?".

VARIABLE LABELS V161 "[Mede-patiënten die ook om euthanasie verzochten] Heeft u tijdens deze euthanasieprocedure de volgende ervaringen beleefd?".

VARIABLE LABELS V162 "[Er werden uiteindelijk geen adviezen verstrekt ] Wat was de aard van het (de) uiteindelijk verstrekte advies (adviezen) in functie van het euthanasieverzoek?".

VARIABLE LABELS V163 "[Hierover werd ik niet geïnformeerd] Wat was de aard van het (de) uiteindelijk verstrekte advies (adviezen) in functie van het euthanasieverzoek?".

VARIABLE LABELS V164 "[Er werden wettelijk vereiste adviezen verstrekt] Wat was de aard van het (de) uiteindelijk verstrekte advies (adviezen) in functie van het euthanasieverzoek?".

VARIABLE LABELS V165 "[Er werden meer dan enkel de wettelijk vereiste adviezen verstrekt] Wat was de aard van het (de) uiteindelijk verstrekte advies (adviezen) in functie van het euthanasieverzoek?".

VARIABLE LABELS V166 "[Positieve adviezen:] Hoeveel positieve dan wel negatieve adviezen werden er verstrekt?".

VARIABLE LABELS V167 "[Negatieve adviezen:] Hoeveel positieve dan wel negatieve adviezen werden er verstrekt?".

VARIABLE LABELS V168 "Is de patiënt overleden via euthanasie?".

VARIABLE LABELS V169 "Wil u hierover nog iets verduidelijken?".

VARIABLE LABELS V170 "[Ja, en ik heb het letaal middel zelf toegediend (ev. geassisteerd door een ervaren collega)] Was u aanwezig tijdens de euthanasie?".

VARIABLE LABELS V171 "[Ja, en ik heb het middel voorbereid, waarna een andere zorgverlener het aan de patiënt toediende] Was u aanwezig tijdens de euthanasie?".

VARIABLE LABELS V172 "[Ja, en ik heb het middel voorbereid, waarna de patiënt het zelf innam] Was u aanwezig tijdens de euthanasie?".

VARIABLE LABELS V173 "[Ja, maar ik heb het middel niet voorbereid en ook niet toegediend] Was u aanwezig tijdens de euthanasie?".

VARIABLE LABELS V174 "[Nee] Was u aanwezig tijdens de euthanasie?".

VARIABLE LABELS V175 "[Nee] Heeft u voor uzelf emotionele ondersteuning gezocht tijdens of na de euthanasieprocedure?".

VARIABLE LABELS V176 "[Ja, in privékring] Heeft u voor uzelf emotionele ondersteuning gezocht tijdens of na de euthanasieprocedure?".

VARIABLE LABELS V177 "[Ja, bij collega’s] Heeft u voor uzelf emotionele ondersteuning gezocht tijdens of na de euthanasieprocedure?".

VARIABLE LABELS V178 "[Ja, bij externe professionele hulpverlening] Heeft u voor uzelf emotionele ondersteuning gezocht tijdens of na de euthanasieprocedure?".

VARIABLE LABELS V179 "[Ja, bij anderen] Heeft u voor uzelf emotionele ondersteuning gezocht tijdens of na de euthanasieprocedure?".

VARIABLE LABELS V180 "Heeft deze casus uw houding ten aanzien van toekomstige verzoeken beïnvloed?".

VARIABLE LABELS V181 "Op welke manier heeft het uw houding veranderd?".

VARIABLE LABELS V182 "Wil u nog iets verduidelijken of toelichten over uw ervaring/gevoelens m.b.t. deze casus?".

VARIABLE LABELS V183 "[Slechte timing, deelname past nu even niet in mijn drukke agenda] Hartelijk dank om onze onderzoeksvoorwaarden te lezen. U heeft echter aangegeven niet te willen deelnemen aan ons onderzoek.Zou u hieronder kunnen aangeven waar"+

"om u de vragenlijst liever niet invult?U kan deze enquête alleszins nu meteen of na het aanduiden van uw reden(en)van niet-deelname afsluiten. ".

VARIABLE LABELS V184 "[Deelname kost te veel tijd] Hartelijk dank om onze onderzoeksvoorwaarden te lezen. U heeft echter aangegeven niet te willen deelnemen aan ons onderzoek.Zou u hieronder kunnen aangeven waarom u de vragenlijst liever niet invult"+

"?U kan deze enquête alleszins nu meteen of na het aanduiden van uw reden(en)van niet-deelname afsluiten. ".

VARIABLE LABELS V185 "[Ik heb geen interesse in het onderwerp ] Hartelijk dank om onze onderzoeksvoorwaarden te lezen. U heeft echter aangegeven niet te willen deelnemen aan ons onderzoek.Zou u hieronder kunnen aangeven waarom u de vragenlijst lieve"+

"r niet invult?U kan deze enquête alleszins nu meteen of na het aanduiden van uw reden(en)van niet-deelname afsluiten. ".

VARIABLE LABELS V186 "[Ik vermoed dat het een inbreuk is op mijn privacy] Hartelijk dank om onze onderzoeksvoorwaarden te lezen. U heeft echter aangegeven niet te willen deelnemen aan ons onderzoek.Zou u hieronder kunnen aangeven waarom u de vragen"+

"lijst liever niet invult?U kan deze enquête alleszins nu meteen of na het aanduiden van uw reden(en)van niet-deelname afsluiten. ".

VARIABLE LABELS V187 "[Ik neem nooit deel aan survey onderzoek] Hartelijk dank om onze onderzoeksvoorwaarden te lezen. U heeft echter aangegeven niet te willen deelnemen aan ons onderzoek.Zou u hieronder kunnen aangeven waarom u de vragenlijst lieve"+

"r niet invult?U kan deze enquête alleszins nu meteen of na het aanduiden van uw reden(en)van niet-deelname afsluiten. ".

VARIABLE LABELS V188 "[Ik heb geen vertrouwen in survey onderzoek] Hartelijk dank om onze onderzoeksvoorwaarden te lezen. U heeft echter aangegeven niet te willen deelnemen aan ons onderzoek.Zou u hieronder kunnen aangeven waarom u de vragenlijst li"+

"ever niet invult?U kan deze enquête alleszins nu meteen of na het aanduiden van uw reden(en)van niet-deelname afsluiten. ".

VARIABLE LABELS V189 "[Andere] Hartelijk dank om onze onderzoeksvoorwaarden te lezen. U heeft echter aangegeven niet te willen deelnemen aan ons onderzoek.Zou u hieronder kunnen aangeven waarom u de vragenlijst liever niet invult?U kan deze enquête "+

"alleszins nu meteen of na het aanduiden van uw reden(en)van niet-deelname afsluiten. ".

*Define Value labels.

VALUE LABELS V15

1 "Ja, ik ga akkoord met deelname."

0 "Nee, ik ga niet akkoord met deelname.".

VALUE LABELS V16

1 "Ja"

2 "Nee".

VALUE LABELS V17

1 "Ja"

0 "Niet geselecteerd".

VALUE LABELS V18

1 "Ja"

0 "Niet geselecteerd".

VALUE LABELS V19

1 "Ja"

0 "Niet geselecteerd".

VALUE LABELS V20

1 "Ja"

0 "Niet geselecteerd".

VALUE LABELS V21

1 "Ja"

0 "Niet geselecteerd".

VALUE LABELS V22

1 "Ja"

0 "Niet geselecteerd".

VALUE LABELS V23

1 "Ja"

0 "Niet geselecteerd".

VALUE LABELS V25

0 "Minder dan 5 jaar"

1 "6 tot 10 jaar"

2 "11 tot 20 jaar"

3 "Meer dan 20 jaar".

VALUE LABELS V26

1 "Ja"

2 "Nee".

VALUE LABELS V27

1 "Ja"

2 "Nee".

VALUE LABELS V28

0 "Jonger dan 30"

1 "30 - 40 jaar"

2 "41 - 60 jaar"

3 "Ouder dan 60".

VALUE LABELS V29

0 "Man"

1 "Vrouw"

2 "X".

VALUE LABELS V30

1 "Helemaal oneens"

2 "Oneens"

3 "Neutraal"

4 "Eens"

5 "Helemaal eens".

VALUE LABELS V31

1 "Helemaal oneens"

2 "Oneens"

3 "Neutraal"

4 "Eens"

5 "Helemaal eens".

VALUE LABELS V32

1 "Helemaal oneens"

2 "Oneens"

3 "Neutraal"

4 "Eens"

5 "Helemaal eens".

VALUE LABELS V33

1 "Helemaal oneens"

2 "Oneens"

3 "Neutraal"

4 "Eens"

5 "Helemaal eens".

VALUE LABELS V34

1 "Helemaal oneens"

2 "Oneens"

3 "Neutraal"

4 "Eens"

5 "Helemaal eens".

VALUE LABELS V35

1 "Helemaal oneens"

2 "Oneens"

3 "Neutraal"

4 "Eens"

5 "Helemaal eens".

VALUE LABELS V36

1 "Helemaal oneens"

2 "Oneens"

3 "Neutraal"

4 "Eens"

5 "Helemaal eens".

VALUE LABELS V37

1 "Helemaal oneens"

2 "Oneens"

3 "Neutraal"

4 "Eens"

5 "Helemaal eens".

VALUE LABELS V38

1 "Helemaal oneens"

2 "Oneens"

3 "Neutraal"

4 "Eens"

5 "Helemaal eens".

VALUE LABELS V39

1 "Helemaal oneens"

2 "Oneens"

3 "Neutraal"

4 "Eens"

5 "Helemaal eens".

VALUE LABELS V40

1 "Helemaal oneens"

2 "Oneens"

3 "Neutraal"

4 "Eens"

5 "Helemaal eens".

VALUE LABELS V41

1 "Helemaal oneens"

2 "Oneens"

3 "Neutraal"

4 "Eens"

5 "Helemaal eens".

VALUE LABELS V42

1 "Helemaal oneens"

2 "Oneens"

3 "Neutraal"

4 "Eens"

5 "Helemaal eens".

VALUE LABELS V43

1 "Ja"

2 "Nee".

VALUE LABELS V45

1 "Ja"

0 "Niet geselecteerd".

VALUE LABELS V46

1 "Ja"

0 "Niet geselecteerd".

VALUE LABELS V47

1 "Ja"

0 "Niet geselecteerd".

VALUE LABELS V48

1 "Ja"

0 "Niet geselecteerd".

VALUE LABELS V49

1 "Ja"

0 "Niet geselecteerd".

VALUE LABELS V50

1 "Ja"

0 "Niet geselecteerd".

VALUE LABELS V51

1 "Ja"

0 "Niet geselecteerd".

VALUE LABELS V52

1 "Ja"

0 "Niet geselecteerd".

VALUE LABELS V53

1 "Ja"

0 "Niet geselecteerd".

VALUE LABELS V55

0 "0 patiënten"

1 "1-2 patiënten"

2 "3-5 patiënten"

3 "5-9 patiënten"

4 "10-20 patiënten"

5 "> 20 patiënten".

VALUE LABELS V56

"A1" "niet van toepassing"

"A2" "1-2 patiënten"

"A3" "3-5 patiënten"

"A4" "meer dan 5 patiënten".

VALUE LABELS V57

"A1" "niet van toepassing"

"A2" "1-2 patiënten"

"A3" "3-5 patiënten"

"A4" "meer dan 5 patiënten".

VALUE LABELS V58

"A1" "niet van toepassing"

"A2" "1-2 patiënten"

"A3" "3-5 patiënten"

"A4" "meer dan 5 patiënten".

VALUE LABELS V59

0 "0 patiënten"

1 "1-2 patiënten"

2 "3-5 patiënten"

3 "meer dan 5 patiënten".

VALUE LABELS V60

1 "Ja"

0 "Niet geselecteerd".

VALUE LABELS V61

1 "Ja"

0 "Niet geselecteerd".

VALUE LABELS V62

1 "Ja"

0 "Niet geselecteerd".

VALUE LABELS V63

1 "Ja"

0 "Niet geselecteerd".

VALUE LABELS V64

1 "Ja"

0 "Niet geselecteerd".

VALUE LABELS V65

1 "Ja"

0 "Niet geselecteerd".

VALUE LABELS V66

1 "Ja"

0 "Niet geselecteerd".

VALUE LABELS V67

1 "Ja"

0 "Niet geselecteerd".

VALUE LABELS V68

1 "Ja"

0 "Niet geselecteerd".

VALUE LABELS V70

1 "Ja"

0 "Niet geselecteerd".

VALUE LABELS V71

1 "Ja"

0 "Niet geselecteerd".

VALUE LABELS V72

1 "Ja"

0 "Niet geselecteerd".

VALUE LABELS V76

1 "Ja"

0 "Nee".

VALUE LABELS V77

1 "Ja"

0 "Niet geselecteerd".

VALUE LABELS V78

1 "Ja"

0 "Niet geselecteerd".

VALUE LABELS V79

1 "Ja"

0 "Niet geselecteerd".

VALUE LABELS V80

1 "Ja"

0 "Niet geselecteerd".

VALUE LABELS V81

1 "Ja"

0 "Niet geselecteerd".

VALUE LABELS V82

1 "Ja"

0 "Niet geselecteerd".

VALUE LABELS V89

1 "Ja"

0 "Niet geselecteerd".

VALUE LABELS V90

1 "Ja"

0 "Niet geselecteerd".

VALUE LABELS V91

1 "Ja"

0 "Niet geselecteerd".

VALUE LABELS V92

1 "Ja"

0 "Niet geselecteerd".

VALUE LABELS V93

1 "Ja"

0 "Niet geselecteerd".

VALUE LABELS V94

1 "Ja"

0 "Niet geselecteerd".

VALUE LABELS V96

1 "Ja"

0 "Niet geselecteerd".

VALUE LABELS V98

1 "Ja"

0 "Niet geselecteerd".

VALUE LABELS V100

1 "Ja"

0 "Niet geselecteerd".

VALUE LABELS V102

1 "Ja"

0 "Niet geselecteerd".

VALUE LABELS V103

1 "Ja"

0 "Niet geselecteerd".

VALUE LABELS V104

1 "Ja"

0 "Niet geselecteerd".

VALUE LABELS V105

1 "Ja"

0 "Niet geselecteerd".

VALUE LABELS V106

1 "Ja"

0 "Niet geselecteerd".

VALUE LABELS V107

1 "Ja"

0 "Niet geselecteerd".

VALUE LABELS V108

1 "Ja"

0 "Niet geselecteerd".

VALUE LABELS V109

1 "Ja"

0 "Niet geselecteerd".

VALUE LABELS V110

1 "Ja"

0 "Niet geselecteerd".

VALUE LABELS V111

1 "Ja"

0 "Niet geselecteerd".

VALUE LABELS V112

1 "Ja"

0 "Niet geselecteerd".

VALUE LABELS V113

1 "Ja"

0 "Niet geselecteerd".

VALUE LABELS V114

1 "Ja"

0 "Niet geselecteerd".

VALUE LABELS V115

1 "Ja"

0 "Niet geselecteerd".

VALUE LABELS V116

1 "Ja"

0 "Niet geselecteerd".

VALUE LABELS V118

1 "Ja"

0 "Niet geselecteerd".

VALUE LABELS V119

1 "Ja"

0 "Niet geselecteerd".

VALUE LABELS V120

1 "Ja"

0 "Niet geselecteerd".

VALUE LABELS V121

1 "Ja"

0 "Niet geselecteerd".

VALUE LABELS V122

1 "Ja"

0 "Niet geselecteerd".

VALUE LABELS V123

1 "Ja"

0 "Niet geselecteerd".

VALUE LABELS V124

1 "Ja"

0 "Niet geselecteerd".

VALUE LABELS V125

1 "Ja"

0 "Niet geselecteerd".

VALUE LABELS V126

1 "Ja"

0 "Niet geselecteerd".

VALUE LABELS V127

1 "Ja"

0 "Niet geselecteerd".

VALUE LABELS V128

1 "Ja"

0 "Niet geselecteerd".

VALUE LABELS V129

1 "Ja"

0 "Niet geselecteerd".

VALUE LABELS V130

1 "Ja"

0 "Niet geselecteerd".

VALUE LABELS V132

1 "Ja"

0 "Niet geselecteerd".

VALUE LABELS V133

1 "Ja"

0 "Niet geselecteerd".

VALUE LABELS V134

1 "Ja"

0 "Niet geselecteerd".

VALUE LABELS V135

1 "Ja"

0 "Niet geselecteerd".

VALUE LABELS V136

1 "Ja"

0 "Nee".

VALUE LABELS V137

1 "Ja"

0 "Nee".

VALUE LABELS V138

1 "Ja"

0 "Nee".

VALUE LABELS V139

1 "Ja"

0 "Nee".

VALUE LABELS V140

1 "Ja"

0 "Nee".

VALUE LABELS V141

1 "Ja"

0 "Nee".

VALUE LABELS V142

1 "Geen"

2 "Weinig"

3 "Gemiddeld"

4 "Veel"

5 "Erg Veel".

VALUE LABELS V143

1 "Geen"

2 "Weinig"

3 "Gemiddeld"

4 "Veel"

5 "Erg Veel".

VALUE LABELS V144

1 "Geen"

2 "Weinig"

3 "Gemiddeld"

4 "Veel"

5 "Erg Veel".

VALUE LABELS V145

1 "Geen"

2 "Weinig"

3 "Gemiddeld"

4 "Veel"

5 "Erg Veel".

VALUE LABELS V146

1 "Geen"

2 "Weinig"

3 "Gemiddeld"

4 "Veel"

5 "Erg Veel".

VALUE LABELS V147

1 "Geen"

2 "Weinig"

3 "Gemiddeld"

4 "Veel"

5 "Erg Veel".

VALUE LABELS V149

1 "Ja"

0 "Nee".

VALUE LABELS V150

1 "Ja"

0 "Nee".

VALUE LABELS V151

1 "Ja"

0 "Nee".

VALUE LABELS V152

1 "Ja"

0 "Nee".

VALUE LABELS V153

1 "Ja"

0 "Nee".

VALUE LABELS V154

1 "Ja"

0 "Nee".

VALUE LABELS V155

1 "Ja"

0 "Nee".

VALUE LABELS V156

1 "Ja"

0 "Nee".

VALUE LABELS V157

1 "Ja"

0 "Nee".

VALUE LABELS V158

1 "Ja"

0 "Nee".

VALUE LABELS V159

1 "Ja"

0 "Nee".

VALUE LABELS V160

1 "Ja"

0 "Nee".

VALUE LABELS V161

1 "Ja"

0 "Nee".

VALUE LABELS V162

1 "Ja"

0 "Niet geselecteerd".

VALUE LABELS V163

1 "Ja"

0 "Niet geselecteerd".

VALUE LABELS V164

1 "Ja"

0 "Niet geselecteerd".

VALUE LABELS V165

1 "Ja"

0 "Niet geselecteerd".

VALUE LABELS V168

0 "Weet ik niet"

1 "Ja"

2 "Nee, de euthanasieprocedure is nog niet afgerond"

3 "Nee, de patiënt trok het euthanasieverzoek zonder druk van derden terug in"

4 "Nee, de patiënt trok het euthanasieverzoek onder druk van derden terug in"

5 "Nee, de patiënt is op een andere manier overleden"

6 "Nee, wegens een andere dan de hierboven genoemde redenen".

VALUE LABELS V170

1 "Ja"

0 "Niet geselecteerd".

VALUE LABELS V171

1 "Ja"

0 "Niet geselecteerd".

VALUE LABELS V172

1 "Ja"

0 "Niet geselecteerd".

VALUE LABELS V173

1 "Ja"

0 "Niet geselecteerd".

VALUE LABELS V174

1 "Ja"

0 "Niet geselecteerd".

VALUE LABELS V175

1 "Ja"

0 "Niet geselecteerd".

VALUE LABELS V176

1 "Ja"

0 "Niet geselecteerd".

VALUE LABELS V177

1 "Ja"

0 "Niet geselecteerd".

VALUE LABELS V178

1 "Ja"

0 "Niet geselecteerd".

VALUE LABELS V179

1 "Ja"

0 "Niet geselecteerd".

VALUE LABELS V180

1 "Ja"

2 "Nee".

VALUE LABELS V183

1 "Ja"

0 "Niet geselecteerd".

VALUE LABELS V184

1 "Ja"

0 "Niet geselecteerd".

VALUE LABELS V185

1 "Ja"

0 "Niet geselecteerd".

VALUE LABELS V186

1 "Ja"

0 "Niet geselecteerd".

VALUE LABELS V187

1 "Ja"

0 "Niet geselecteerd".

VALUE LABELS V188

1 "Ja"

0 "Niet geselecteerd".

RENAME VARIABLE ( V1 = firstname ).

RENAME VARIABLE ( V2 = lastname ).

RENAME VARIABLE ( V3 = email ).

RENAME VARIABLE ( V4 = emailstatus ).

RENAME VARIABLE ( V5 = language ).

RENAME VARIABLE ( V6 = sent ).

RENAME VARIABLE ( V7 = remindersent ).

RENAME VARIABLE ( V8 = remindercount ).

RENAME VARIABLE ( V9 = usesleft ).

RENAME VARIABLE ( V10 = id ).

RENAME VARIABLE ( V11 = token ).

RENAME VARIABLE ( V12 = submitdate ).

RENAME VARIABLE ( V13 = lastpage ).

RENAME VARIABLE ( V14 = startlanguage ).

RENAME VARIABLE ( V15 = IC1 ).

RENAME VARIABLE ( V16 = WorkasPsychNL ).

RENAME VARIABLE ( V17 = WhereWorkNL_WorkPrivNL ).

RENAME VARIABLE ( V18 = WhereWorkNL_WorkHospNL ).

RENAME VARIABLE ( V19 = WhereWorkNL_WorkCentNL ).

RENAME VARIABLE ( V20 = WhereWorkNL_WorkPyschCareNL ).

RENAME VARIABLE ( V21 = WhereWorkNL_WorkPsychHomeNL ).

RENAME VARIABLE ( V22 = WhereWorkNL_WorkAsstLivNL ).

RENAME VARIABLE ( V23 = WhereWorkNL_WorkAsstOthNL ).

RENAME VARIABLE ( V24 = SpecWhereWork ).

RENAME VARIABLE ( V25 = YrsAsPsychNL ).

RENAME VARIABLE ( V26 = SpecialEducEOLNL ).

RENAME VARIABLE ( V27 = CompetenceEUNL ).

RENAME VARIABLE ( V28 = AgeNL ).

RENAME VARIABLE ( V29 = SexNL ).

RENAME VARIABLE ( V30 = eenoneensvragen_STOnlyTermNL ).

RENAME VARIABLE ( V31 = eenoneensvragen_STOnlySomatNL ).

RENAME VARIABLE ( V32 = eenoneensvragen_STNotPsychNL ).

RENAME VARIABLE ( V33 = eenoneensvragen_STYesPsychHelpRelNL ).

RENAME VARIABLE ( V34 = eenoneensvragen_STMAIDNL ).

RENAME VARIABLE ( V35 = eenoneensvragen_STHopelessNL ).

RENAME VARIABLE ( V36 = eenoneensvragen_STExtremeSuffNL ).

RENAME VARIABLE ( V37 = eenoneensvragen_STNoPerpectiveNL ).

RENAME VARIABLE ( V38 = eenoneensvragen_STEuthPrevSuicNL ).

RENAME VARIABLE ( V39 = eenoneensvragen_STFutureMedOptionsNL ).

RENAME VARIABLE ( V40 = eenoneensvragen_STWholeLifeNL ).

RENAME VARIABLE ( V41 = eenoneensvragen_STWellConsiderNL ).

RENAME VARIABLE ( V42 = eenoneensvragen_STTooPermissiveNL ).

RENAME VARIABLE ( V43 = RefEuNL ).

RENAME VARIABLE ( V44 = WhyRefEuNL ).

RENAME VARIABLE ( V45 = InvolvEuNL_0 ).

RENAME VARIABLE ( V46 = InvolvEuNL_1 ).

RENAME VARIABLE ( V47 = InvolvEuNL_2 ).

RENAME VARIABLE ( V48 = InvolvEuNL_3 ).

RENAME VARIABLE ( V49 = InvolvEuNL_4 ).

RENAME VARIABLE ( V50 = InvolvEuNL_5 ).

RENAME VARIABLE ( V51 = InvolvEuNL_6 ).

RENAME VARIABLE ( V52 = InvolvEuNL_7 ).

RENAME VARIABLE ( V53 = InvolvEuNL_8 ).

RENAME VARIABLE ( V54 = SpecInvolNL ).

RENAME VARIABLE ( V55 = NumberEuReqNL ).

RENAME VARIABLE ( V56 = OftenAdv_SQ001 ).

RENAME VARIABLE ( V57 = OftenAdv_SQ002 ).

RENAME VARIABLE ( V58 = OftenAdv_SQ003 ).

RENAME VARIABLE ( V59 = NumberEUPerformNL ).

RENAME VARIABLE ( V60 = FutureRolesNL_SQ009 ).

RENAME VARIABLE ( V61 = FutureRolesNL_SQ001 ).

RENAME VARIABLE ( V62 = FutureRolesNL_SQ002 ).

RENAME VARIABLE ( V63 = FutureRolesNL_SQ003 ).

RENAME VARIABLE ( V64 = FutureRolesNL_SQ004 ).

RENAME VARIABLE ( V65 = FutureRolesNL_SQ005 ).

RENAME VARIABLE ( V66 = FutureRolesNL_SQ006 ).

RENAME VARIABLE ( V67 = FutureRolesNL_SQ007 ).

RENAME VARIABLE ( V68 = FutureRolesNL_SQ008 ).

RENAME VARIABLE ( V69 = SpecFutureRoleNL ).

RENAME VARIABLE ( V70 = ExtConsTeamNL_SQ001 ).

RENAME VARIABLE ( V71 = ExtConsTeamNL_SQ002 ).

RENAME VARIABLE ( V72 = ExtConsTeamNL_SQ003 ).

RENAME VARIABLE ( V73 = WhichConsteamNL ).

RENAME VARIABLE ( V74 = WhyNoExtConsTeamNL ).

RENAME VARIABLE ( V75 = AddInfoPart1NL ).

RENAME VARIABLE ( V76 = FullExp ).

RENAME VARIABLE ( V77 = SpecFuncNL_1 ).

RENAME VARIABLE ( V78 = SpecFuncNL_2 ).

RENAME VARIABLE ( V79 = SpecFuncNL_3 ).

RENAME VARIABLE ( V80 = SpecFuncNL_4 ).

RENAME VARIABLE ( V81 = SpecFuncNL_5 ).

RENAME VARIABLE ( V82 = SpecFuncNL_6 ).

RENAME VARIABLE ( V83 = TimeEuProcNL_DeciWksNL ).

RENAME VARIABLE ( V84 = TimeEuProcNL_DeciMonthsNL ).

RENAME VARIABLE ( V85 = TimeEuProcNL_DeciYrsNL ).

RENAME VARIABLE ( V86 = TimeBefEuReqNL_TxWksNL ).

RENAME VARIABLE ( V87 = TimeBefEuReqNL_TxMonthsNL ).

RENAME VARIABLE ( V88 = TimeBefEuReqNL_TxYrsNL ).

RENAME VARIABLE ( V89 = PsychTherNL_1 ).

RENAME VARIABLE ( V90 = PsychTherNL_2 ).

RENAME VARIABLE ( V91 = PsychTherNL_3 ).

RENAME VARIABLE ( V92 = PsychTherNL_4 ).

RENAME VARIABLE ( V93 = PsychTherNL_5 ).

RENAME VARIABLE ( V94 = PsychTherNL_6 ).

RENAME VARIABLE ( V95 = WhichTherNL ).

RENAME VARIABLE ( V96 = ImportPathNL_PathClinNL ).

RENAME VARIABLE ( V97 = ImportPathNL_PathClinNLcomment ).

RENAME VARIABLE ( V98 = ImportPathNL_PathPersNL ).

RENAME VARIABLE ( V99 = ImportPathNL_PathPersNLcomment ).

RENAME VARIABLE ( V100 = ImportPathNL_PathSomaNL ).

RENAME VARIABLE ( V101 = ImportPathNL_PathSomaNLcomment ).

RENAME VARIABLE ( V102 = ReasEuReqNL_1 ).

RENAME VARIABLE ( V103 = ReasEuReqNL_2 ).

RENAME VARIABLE ( V104 = ReasEuReqNL_3 ).

RENAME VARIABLE ( V105 = ReasEuReqNL_4 ).

RENAME VARIABLE ( V106 = ReasEuReqNL_5 ).

RENAME VARIABLE ( V107 = ReasEuReqNL_6 ).

RENAME VARIABLE ( V108 = ReasEuReqNL_7 ).

RENAME VARIABLE ( V109 = ReasEuReqNL_8 ).

RENAME VARIABLE ( V110 = ReasEuReqNL_9 ).

RENAME VARIABLE ( V111 = ReasEuReqNL_10 ).

RENAME VARIABLE ( V112 = ReasEuReqNL_11 ).

RENAME VARIABLE ( V113 = ReasEuReqNL_12 ).

RENAME VARIABLE ( V114 = ReasEuReqNL_13 ).

RENAME VARIABLE ( V115 = ReasEuReqNL_14 ).

RENAME VARIABLE ( V116 = ReasEuReqNL_15 ).

RENAME VARIABLE ( V117 = TwoReasEuNL ).

RENAME VARIABLE ( V118 = ConsCaregiversNL_1 ).

RENAME VARIABLE ( V119 = ConsCaregiversNL_2 ).

RENAME VARIABLE ( V120 = ConsCaregiversNL_3 ).

RENAME VARIABLE ( V121 = ConsCaregiversNL_4 ).

RENAME VARIABLE ( V122 = ConsCaregiversNL_5 ).

RENAME VARIABLE ( V123 = ConsCaregiversNL_6 ).

RENAME VARIABLE ( V124 = ConsCaregiversNL_7 ).

RENAME VARIABLE ( V125 = ConsCaregiversNL_8 ).

RENAME VARIABLE ( V126 = ConsCaregiversNL_9 ).

RENAME VARIABLE ( V127 = ConsCaregiversNL_10 ).

RENAME VARIABLE ( V128 = ConsCaregiversNL_11 ).

RENAME VARIABLE ( V129 = ConsCaregiversNL_12 ).

RENAME VARIABLE ( V130 = ConsCaregiversNL_13 ).

RENAME VARIABLE ( V131 = WhichOtherPrContNL ).

RENAME VARIABLE ( V132 = ConsFamFriNL_1 ).

RENAME VARIABLE ( V133 = ConsFamFriNL_2 ).

RENAME VARIABLE ( V134 = ConsFamFriNL_3 ).

RENAME VARIABLE ( V135 = ConsFamFriNL_4 ).

RENAME VARIABLE ( V136 = OpinOfPtNL_MentCompNL ).

RENAME VARIABLE ( V137 = OpinOfPtNL_IncurCondNL ).

RENAME VARIABLE ( V138 = OpinOfPtNL_UnbearSuffNL ).

RENAME VARIABLE ( V139 = OpinOfPtNL_HopMedCondNL ).

RENAME VARIABLE ( V140 = OpinOfPtNL_LackResTherNL ).

RENAME VARIABLE ( V141 = OpinOfPtNL_VolunReqNL ).

RENAME VARIABLE ( V142 = OpinOfPtScaleNL_MentCompScaleNL ).

RENAME VARIABLE ( V143 = OpinOfPtScaleNL_IncurCondScaleNL ).

RENAME VARIABLE ( V144 = OpinOfPtScaleNL_UnbearSuffScaleNL ).

RENAME VARIABLE ( V145 = OpinOfPtScaleNL_HopMedCondScaleNL ).

RENAME VARIABLE ( V146 = OpinOfPtScaleNL_LackResTherScaleNL ).

RENAME VARIABLE ( V147 = OpinOfPtScaleNL_VolunReqScaleNL ).

RENAME VARIABLE ( V148 = OtherOpinTextNL ).

RENAME VARIABLE ( V149 = TypePressNL_Press3PartNL ).

RENAME VARIABLE ( V150 = TypePressNL_PressPtNL ).

RENAME VARIABLE ( V151 = TypePressNL_PressFamYesNL ).

RENAME VARIABLE ( V152 = TypePressNL_PressFamNoNL ).

RENAME VARIABLE ( V153 = TypePressNL_PressCollNoNL ).

RENAME VARIABLE ( V154 = TypePressNL_PressCollYesNL ).

RENAME VARIABLE ( V155 = TypePressNL_PressCarInstNoNL ).

RENAME VARIABLE ( V156 = TypePressNL_PressCarInstYesNL ).

RENAME VARIABLE ( V157 = ConfExperNL_SelfEmoBurdNL ).

RENAME VARIABLE ( V158 = ConfExperNL_NewTherChanNL ).

RENAME VARIABLE ( V159 = ConfExperNL_PtLowSuicRiskNL ).

RENAME VARIABLE ( V160 = ConfExperNL_RestRelatNL ).

RENAME VARIABLE ( V161 = ConfExperNL_OthPtAlsoEuNL ).

RENAME VARIABLE ( V162 = FinalAdvNL_SQ001 ).

RENAME VARIABLE ( V163 = FinalAdvNL_SQ002 ).

RENAME VARIABLE ( V164 = FinalAdvNL_SQ003 ).

RENAME VARIABLE ( V165 = FinalAdvNL_SQ004 ).

RENAME VARIABLE ( V166 = NatureFinalAdvNL_SQ001 ).

RENAME VARIABLE ( V167 = NatureFinalAdvNL_SQ002 ).

RENAME VARIABLE ( V168 = DeathEuNL ).

RENAME VARIABLE ( V169 = DeathOtherWhyNL ).

RENAME VARIABLE ( V170 = PresentEUNL_1 ).

RENAME VARIABLE ( V171 = PresentEUNL_2 ).

RENAME VARIABLE ( V172 = PresentEUNL_3 ).

RENAME VARIABLE ( V173 = PresentEUNL_4 ).

RENAME VARIABLE ( V174 = PresentEUNL_5 ).

RENAME VARIABLE ( V175 = SelfEmoSuppNL_1 ).

RENAME VARIABLE ( V176 = SelfEmoSuppNL_2 ).

RENAME VARIABLE ( V177 = SelfEmoSuppNL_3 ).

RENAME VARIABLE ( V178 = SelfEmoSuppNL_4 ).

RENAME VARIABLE ( V179 = SelfEmoSuppNL_5 ).

RENAME VARIABLE ( V180 = CaseInfFutNL ).

RENAME VARIABLE ( V181 = OpinChangeNL ).

RENAME VARIABLE ( V182 = AddInfoPartTwoNL ).

RENAME VARIABLE ( V183 = NonPartSurvNL_SQ001 ).

RENAME VARIABLE ( V184 = NonPartSurvNL_SQ002 ).

RENAME VARIABLE ( V185 = NonPartSurvNL_SQ003 ).

RENAME VARIABLE ( V186 = NonPartSurvNL_SQ004 ).

RENAME VARIABLE ( V187 = NonPartSurvNL_SQ005 ).

RENAME VARIABLE ( V188 = NonPartSurvNL_SQ006 ).

RENAME VARIABLE ( V189 = NonPartSurvNL_other ).

RESTORE LOCALE.

************************************************************************************************************************************************************************************************************************************************

## Syntax psychiatrists’ attitudes and readiness to engage

************************************************************************************************************************************************************************************************************************************************

***TABLE 1: Respondents' characteristics***

FREQUENCIES VARIABLES=WorkasPsychNL WhereWorkNLWorkPrivNL WhereWorkNLWorkHospNL

WhereWorkNLWorkCentNL WhereWorkNLWorkPyschCareNL WhereWorkNLWorkPsychHomeNL

WhereWorkNLWorkAsstLivNL WhereWorkNLWorkAsstOthNL SexNL SpecialEducEOLNL CompetenceEUNL

/ORDER=ANALYSIS.

FREQUENCIES VARIABLES=AgeNL YrsAsPsychNL

/STATISTICS=MEDIAN

/ORDER=ANALYSIS.

***TABLE 2: Respondents’ attitudes toward euthanasia in psychiatry. Don't forget to check valid % for items with missings***

FREQUENCIES VARIABLES=STOnlyTermNL STOnlySomatNL STNotPsychNL STYesPsychHelpRelNL STMAIDNL

STHopelessNL STExtremeSuffNL STNoPerpectiveNL STEuthPrevSuicNL STFutureMedOptionsNL STWholeLifeNL

STWellConsiderNL STTooPermissiveNL

/STATISTICS=STDDEV MINIMUM MAXIMUM MEAN

/ORDER=ANALYSIS.

***Table 3: Respondents’ Readiness to engage in Psychiatric Euthanasia procedures***

FREQUENCIES VARIABLES=FutureRolesNLSQ008

/ORDER=ANALYSIS.

***This variable was analysed in a qualitative manner and then re-coded IF no other role was indicated:

“In welke andere rol?”:

1) als behandelend arts de eu-vraag bespreken tijdens therapie (n = 1): recoded into ‘No role’

2) als gepensioneerd arts een collega adviseren (n = 1): recoded into ‘Advising role in a preliminary stage’

3) als lid van een EC de eu-casus bespreken (n = 2): recoded into ‘Advising role in a preliminary stage’

The other respondent had indicated another specific future role***

FREQUENCIES VARIABLES=ExtConsTeamNLSQ001 ExtConsTeamNLSQ002 ExtConsTeamNLSQ003 FutNoRole FutRoleRef

FutRoleAttOwn FutRoleAttOther FutRolePrelAdv FutRoleFormAdv FutRolePerfOwn FutRolePerfOth

MostExtendFutRole

/ORDER=ANALYSIS.

***Additional information to Table 3: Respondents’ readiness to act in multiple roles during euthanasia procedures for psychiatric patients.***

***A new variable as computed to extract 6 potential future roles 'ConcRole', including No Role***

***Two new variables were computed: Combination of Roles + Which future Role was chosen if only 1 future role was indicated***

FREQUENCIES VARIABLES=ConcRoleNone ConcRoleRef ConcRolePrel ConcRoleFormAdv ConcRoleAttPh

ConcRolePerf ConcRoleIfOne CombiFutRole

/ORDER=ANALYSIS.

*** Association Analysis***

CROSSTABS

/TABLES=SexNL BY ConcRoleNone ConcRoleRef ConcRolePrel ConcRoleFormAdv ConcRoleAttPh ConcRolePerf

MostExtendFutRole

/FORMAT=AVALUE TABLES

/STATISTICS=CHISQ

/CELLS=COUNT ROW

/COUNT ROUND CELL.

CROSSTABS

/TABLES=Age3Cat BY ConcRoleNone ConcRoleRef ConcRolePrel ConcRoleFormAdv ConcRoleAttPh ConcRolePerf

MostExtendFutRole

/FORMAT=AVALUE TABLES

/STATISTICS=CHISQ

/CELLS=COUNT ROW

/COUNT ROUND CELL.

CROSSTABS

/TABLES=YearsPsych3Cat BY ConcRoleNone ConcRoleRef ConcRolePrel ConcRoleFormAdv ConcRoleAttPh ConcRolePerf

MostExtendFutRole

/FORMAT=AVALUE TABLES

/STATISTICS=CHISQ

/CELLS=COUNT ROW

/COUNT ROUND CELL.

CROSSTABS

/TABLES=CompetenceEUNL BY ConcRoleNone ConcRoleRef ConcRolePrel ConcRoleFormAdv ConcRoleAttPh ConcRolePerf

MostExtendFutRole

/FORMAT=AVALUE TABLES

/STATISTICS=CHISQ

/CELLS=COUNT ROW

/COUNT ROUND CELL.

T-TEST GROUPS=SexNL(0 1)

/MISSING=ANALYSIS

/VARIABLES=STNotPsychNL

/CRITERIA=CI(.95).

T-TEST GROUPS=CompetenceEUNL(0 1)

/MISSING=ANALYSIS

/VARIABLES=STNotPsychNL

/CRITERIA=CI(.95).

ONEWAY STNotPsychNL BY Age3Cat

/STATISTICS DESCRIPTIVES EFFECTS HOMOGENEITY BROWNFORSYTHE WELCH

/MISSING ANALYSIS

/POSTHOC=TUKEY BONFERRONI GH ALPHA(0.05).

ONEWAY STNotPsychNL BY YearsPsych3Cat

/STATISTICS DESCRIPTIVES EFFECTS HOMOGENEITY BROWNFORSYTHE WELCH

/MISSING ANALYSIS

/POSTHOC=TUKEY BONFERRONI GH ALPHA(0.05).

MEANS TABLES=YearsPsych3Cat Age3Cat BY STNotPsychNL

/STATISTICS ANOVA.

************************************************************************************************************************************************************************************************************************************************

## Data import psychiatrists’ concrete experiences

************************************************************************************************************************************************************************************************************************************************

***TABLE 1: Respondents' characteristics***

FREQUENCIES VARIABLES=WorkasPsychNL WhereWorkNLWorkPrivNL WhereWorkNLWorkHospNL

WhereWorkNLWorkCentNL WhereWorkNLWorkPyschCareNL WhereWorkNLWorkPsychHomeNL

WhereWorkNLWorkAsstLivNL WhereWorkNLWorkAsstOthNL SexNL SpecialEducEOLNL CompetenceEUNL

ConcRoleNone

/ORDER=ANALYSIS.

FREQUENCIES VARIABLES=AgeNL YrsAsPsychNL

/STATISTICS=MEDIAN

/ORDER=ANALYSIS.

***TABLE2: Respondents’ involvement with psychiatric euthanasia cases throughout their career ***

FREQUENCIES VARIABLES=EverConfronted RefEuNL InvNoRole InvRoleRef InvRoleAttOwn InvRoleAttOther InvRolePrel

InvRoleFormAdv InvRolePerfOwn InvRolePerfOther MostExtInvolv

/ORDER=ANALYSIS.

FREQUENCIES VARIABLES=InvNone InvRef InvPrel InvFormAdv InvAtt InvPerf MostExtInvolv CombiInvolv

IfOneInvolv

/STATISTICS=MEDIAN

/ORDER=ANALYSIS.

***TABLE 3: Respondents’ concrete involvement with psychiatric euthanasia cases***

FREQUENCIES VARIABLES=NumberEuReqNL OftenAdvSQ001 OftenAdvSQ002 OftenAdvSQ003 NumberEUPerformNL

/ORDER=ANALYSIS.

***Table 4:Hypotheses testing concrete involvement in terms of respondent’s specific role***

CROSSTABS

/TABLES=SexNL CompetenceEUNL BY InvNone InvRef InvPrel InvFormAdv InvAtt InvPerf MostExtInvolv

/FORMAT=AVALUE TABLES

/STATISTICS=CHISQ

/CELLS=COUNT ROW

/COUNT ROUND CELL.

CROSSTABS

/TABLES=Age3Cat YearsPsych3Cat BY InvNone InvRef InvPrel InvFormAdv InvAtt InvPerf MostExtInvolv

MostExtendFutRole

/FORMAT=AVALUE TABLES

/STATISTICS=CHISQ

/CELLS=COUNT ROW

/COUNT ROUND CELL.

***Nonparametric Tests: Independent Samples.***

***Kolmogorov-Smirnov for Sex & Competence***

NPTESTS

/INDEPENDENT TEST (MostExtInvolv) GROUP (SexNL) KOLMOGOROV_SMIRNOV

/MISSING SCOPE=ANALYSIS USERMISSING=EXCLUDE

/CRITERIA ALPHA=0.05 CILEVEL=95.

NPAR TESTS

/K-S= MostExtInvolv BY SexNL(0 1)

/STATISTICS=DESCRIPTIVES

/MISSING ANALYSIS.

NPTESTS

/INDEPENDENT TEST (MostExtInvolv) GROUP (CompetenceEUNL) KOLMOGOROV_SMIRNOV

/MISSING SCOPE=ANALYSIS USERMISSING=EXCLUDE

/CRITERIA ALPHA=0.05 CILEVEL=95.

NPAR TESTS

/K-S= MostExtInvolv BY CompetenceEUNL(0 1)

/STATISTICS=DESCRIPTIVES

/MISSING ANALYSIS.

***Kruskal-Wallis test for Age and Years Experience, and check for significant differences if p <.05 via Kolmogorov-Smirnov tests***

NPAR TESTS

/K-W=MostExtInvolv BY Age3Cat(1 3)

/STATISTICS DESCRIPTIVES

/MISSING ANALYSIS

NPAR TESTS

/K-S= MostExtInvolv BY Age3Cat(1 2)

/STATISTICS=DESCRIPTIVES

/MISSING ANALYSIS.

NPAR TESTS

/K-S= MostExtInvolv BY Age3Cat(1 3)

/STATISTICS=DESCRIPTIVES

/MISSING ANALYSIS.

NPAR TESTS

/K-S= MostExtInvolv BY Age3Cat(2 3)

/STATISTICS=DESCRIPTIVES

/MISSING ANALYSIS.

NPAR TESTS

/K-W=MostExtInvolv BY YearsPsych3Cat(1 3)

/STATISTICS DESCRIPTIVES

/MISSING ANALYSIS

NPAR TESTS

/K-S= MostExtInvolv BY YearsPsych3Cat(1 2)

/STATISTICS=DESCRIPTIVES

/MISSING ANALYSIS.

NPAR TESTS

/K-S= MostExtInvolv BY YearsPsych3Cat(1 3)

/STATISTICS=DESCRIPTIVES

/MISSING ANALYSIS.

NPAR TESTS

/K-S= MostExtInvolv BY YearsPsych3Cat(2 3)

/STATISTICS=DESCRIPTIVES

/MISSING ANALYSIS.

***TABLE 5: Hypothesis testing regarding Respondents’ being confronted with psychiatric euthanasia cases and requests refused***

CROSSTABS

/TABLES=SexNL CompetenceEUNL BY RefEuNL EverConfronted

/FORMAT=AVALUE TABLES

/STATISTICS=CHISQ

/CELLS=COUNT ROW

/COUNT ROUND CELL.

CROSSTABS

/TABLES=Age3Cat YearsPsych3Cat BY RefEuNL EverConfronted

/FORMAT=AVALUE TABLES

/STATISTICS=CHISQ

/CELLS=COUNT ROW

/COUNT ROUND CELL.

***New Variable 'EverConfrontedDum' for reasons of 0 cells count**

CROSSTABS

/TABLES=SexNL CompetenceEUNL BY RefEuNL EverConfrontedDum InvNone

/FORMAT=AVALUE TABLES

/STATISTICS=CHISQ

/CELLS=COUNT ROW

/COUNT ROUND CELL.

CROSSTABS

/TABLES=Age3Cat YearsPsych3Cat BY RefEuNL EverConfrontedDum InvNone

/FORMAT=AVALUE TABLES

/STATISTICS=CHISQ

/CELLS=COUNT ROW

/COUNT ROUND CELL.

***** New Variables : “AgeCat2” with values: 1 = < 40 and 2 = > 40 (3 wordt 2)

“YearsPsychCat2” with values: 1 = <20 and 2 = >20 (2 wordt 1)***

CROSSTABS

/TABLES=Age2Cat YearsPsych2Cat BY RefEuNL EverConfrontedDum InvNone

/FORMAT=AVALUE TABLES

/STATISTICS=CHISQ

/CELLS=COUNT ROW

/COUNT ROUND CELL.

LOGISTIC REGRESSION VARIABLES InvNone InvRef InvPrel InvFormAdv InvFormAdv

InvAtt InvPerf

/METHOD=ENTER YearsPsych3Cat Age3Cat

/CONTRAST (YearsPsych3Cat)=Indicator.

************************************************************************************************************************************************************************************************************************************************

## Data import: case study

************************************************************************************************************************************************************************************************************************************************

***TABLE1: general characteristics sample***

FREQUENCIES VARIABLES=WorkasPsychNL WhereWorkNLWorkPrivNL WhereWorkNLWorkHospNL

WhereWorkNLWorkCentNL WhereWorkNLWorkPyschCareNL WhereWorkNLWorkPsychHomeNL

WhereWorkNLWorkAsstLivNL WhereWorkNLWorkAsstOthNL SpecWhereWork SpecialEducEOLNL CompetenceEUNL

SexNL YrsAsPsychNL AgeNL

/ORDER=ANALYSIS.

***Comorbiditeit klinische + persoonlijkheidsstoornis***

COMPUTE ComorbClinPers=ImportPathNLPathClinNL + ImportPathNLPathPersNL .

EXECUTE.

***TABLE2: Characteristics of the patients***

FREQUENCIES VARIABLES=ImportPathNLPathClinNL ImportPathNLPathPersNL ComorbClinPers PsychTherNL1

PsychTherNL2 PsychTherNL3 PsychTherNL4 PsychTherNL5 PsychTherNL6 ReasEuReqNL1 ReasEuReqNL2

ReasEuReqNL3 ReasEuReqNL4 ReasEuReqNL5 ReasEuReqNL6 ReasEuReqNL7 ReasEuReqNL8 ReasEuReqNL9

ReasEuReqNL10 ReasEuReqNL11 ReasEuReqNL12 ReasEuReqNL13 ReasEuReqNL14 ReasEuReqNL15

ImportPathNLPathSomaNL

/ORDER=ANALYSIS.

FREQUENCIES VARIABLES=AverageTTreatmentYears

/STATISTICS=STDDEV MINIMUM MAXIMUM MEAN

/ORDER=ANALYSIS.

***TABLE3: Characteristics of the eu-procedure***

FREQUENCIES VARIABLES=SpecFuncNL1 SpecFuncNL2 SpecFuncNL3 SpecFuncNL4 SpecFuncNL5 SpecFuncNL6

ConsCaregiversNL1 ConsCaregiversNL2 ConsCaregiversNL3 ConsCaregiversNL4 ConsCaregiversNL5

ConsCaregiversNL6 ConsCaregiversNL7 ConsCaregiversNL8 ConsCaregiversNL9 ConsCaregiversNL10

ConsCaregiversNL11 ConsCaregiversNL12 ConsCaregiversNL13 WhichOtherPrContNL ConsFamFriNL1

ConsFamFriNL2 ConsFamFriNL3 ConsFamFriNL4 OpinOfPtNLMentCompNL OpinOfPtNLIncurCondNL

OpinOfPtNLUnbearSuffNL OpinOfPtNLHopMedCondNL OpinOfPtNLLackResTherNL OpinOfPtNLVolunReqNL

TypePressNLPress3PartNL TypePressNLPressPtNL TypePressNLPressFamYesNL TypePressNLPressFamNoNL

TypePressNLPressCollNoNL TypePressNLPressCollYesNL TypePressNLPressCarInstNoNL

TypePressNLPressCarInstYesNL ConfExperNLSelfEmoBurdNL ConfExperNLNewTherChanNL

ConfExperNLPtLowSuicRiskNL ConfExperNLRestRelatNL ConfExperNLOthPtAlsoEuNL

/ORDER=ANALYSIS.

***TABLE3: BIS: DURATION OF THE PROCEDURE: DURATION OF THE PROCEDURE IN MONTHS AND PROCEDURE OF TREATMENT IN YEARS.***

FREQUENCIES VARIABLES=AverageTProcedureMonths

/STATISTICS=STDDEV MINIMUM MAXIMUM MEAN

/ORDER=ANALYSIS.

FREQUENCIES VARIABLES=FinalAdvNLSQ001 FinalAdvNLSQ002 FinalAdvNLSQ003 FinalAdvNLSQ004 DeathEuNL

PresentEUNL1 PresentEUNL2 PresentEUNL3 PresentEUNL4 PresentEUNL5

DeathOtherWhyNL

/ORDER=ANALYSIS.

***TABLE4: Difficulties Assessment***

DESCRIPTIVES VARIABLES=OpinOfPtScaleNLMentCompScaleNL OpinOfPtScaleNLIncurCondScaleNL

OpinOfPtScaleNLUnbearSuffScaleNL OpinOfPtScaleNLHopMedCondScaleNL OpinOfPtScaleNLLackResTherScaleNL

OpinOfPtScaleNLVolunReqScaleNL

/STATISTICS=MEAN STDDEV MIN MAX.

FREQUENCIES VARIABLES=OpinOfPtScaleNLMentCompScaleNL OpinOfPtScaleNLIncurCondScaleNL

OpinOfPtScaleNLUnbearSuffScaleNL OpinOfPtScaleNLHopMedCondScaleNL OpinOfPtScaleNLLackResTherScaleNL

OpinOfPtScaleNLVolunReqScaleNL

/STATISTICS=STDDEV MINIMUM MAXIMUM MEAN

/ORDER=ANALYSIS.

***TABLE5: Impact of the eu-procedure***

FREQUENCIES VARIABLES=SelfEmoSuppNL1 SelfEmoSuppNL2 SelfEmoSuppNL3 SelfEmoSuppNL4 SelfEmoSuppNL5

CaseInfFutNL

/ORDER=ANALYSIS.

****TABLE6: via split-file and frequencies + manually checked***

SORT CASES BY DeathEuNL.

SPLIT FILE LAYERED BY DeathEuNL.

DESCRIPTIVES VARIABLES=OpinOfPtScaleNLMentCompScaleNL OpinOfPtScaleNLIncurCondScaleNL

OpinOfPtScaleNLUnbearSuffScaleNL OpinOfPtScaleNLHopMedCondScaleNL OpinOfPtScaleNLLackResTherScaleNL

OpinOfPtScaleNLVolunReqScaleNL

/STATISTICS=MEAN STDDEV MIN MAX.

FREQUENCIES VARIABLES=OpinOfPtScaleNLMentCompScaleNL OpinOfPtScaleNLIncurCondScaleNL

OpinOfPtScaleNLUnbearSuffScaleNL OpinOfPtScaleNLHopMedCondScaleNL OpinOfPtScaleNLLackResTherScaleNL

OpinOfPtScaleNLVolunReqScaleNL

/ORDER=ANALYSIS.

SPLIT FILE off.

***

EXAMINE VARIABLES=AverageTTreatmentYears

/PLOT BOXPLOT HISTOGRAM

/COMPARE GROUPS

/PERCENTILES(5,10,25,50,75,90,95) HAVERAGE

/STATISTICS DESCRIPTIVES EXTREME

/CINTERVAL 95

/MISSING LISTWISE

/NOTOTAL.

EXAMINE VARIABLES=AverageTProcedureMonths

/PLOT BOXPLOT HISTOGRAM

/COMPARE GROUPS

/PERCENTILES(5,10,25,50,75,90,95) HAVERAGE

/STATISTICS DESCRIPTIVES EXTREME

/CINTERVAL 95

/MISSING LISTWISE

/NOTOTAL.

FREQUENCIES VARIABLES=AverageTProcedureMonths

/NTILES=4

/STATISTICS=STDDEV VARIANCE RANGE MINIMUM MAXIMUM SEMEAN MEAN MEDIAN MODE SKEWNESS SESKEW

KURTOSIS SEKURT

/HISTOGRAM NORMAL

/ORDER=ANALYSIS.

************************************************************************************************************************************************************************************************************************************************
