# Supplementary material for: The engagement of psychiatrists in the assessment of euthanasia requests from psychiatric patients in Belgium: a survey study
Source: BMC Psychiatry. 2020 Aug 8;20:400. doi: 10.1186/s12888-020-02792-w (PMC7414658; doi:10.1186/s12888-020-02792-w)
Supplement: Supplementary file 1 — Additional file 1. [file 12888_2020_2792_MOESM1_ESM.zip › Appendix H_Psychiatrists%u2019 demographics and professional characteristics (N %).docx]

**Appendix H**

**Supplemental Material 1: Psychiatrists’ demographics and professional characteristics (N/%)**

| Variables | Sample (*N* = 178)  (N^o^ and %) | |
| --- | --- | --- |
| Gender  Male  Female Unknown | 100 75  3 | 56.2  42.1  1.7 |
| Age (in years)  < 30  30 - 40 years  41 - 60 years  > 60 | 27  39  64  48 | 15.1  21.9  36.0  27.0 |
| Worked as psychiatrist or psychiatric trainee during last year  Yes  No  Unknown | 161  16  1 | 90.4  9.0  0.6 |
| Clinical setting*  Private or Group Practice Psychiatric Hospital Care Community Mental HealthCare Center Psychiatric Nursing Home  Psychiatric Home Care  Sheltered housing Other** | 80  120  22 9  6 12  26 | 44.9 67.4 12.4 5.1 3.4 6.7 14.6 |
| Work experience (in number of years)  < 5 years 6 - 10 years  11 - 20 years > 20 years | 32  20  41  85 | 18.0  11.2  23.0  47.8 |
| Ever received special training in EOL Yes  No  Unknown | 9  167 2 | 5.1  93.8 1.1 |
| Readiness to be involved in euthanasia procedure(s)  Yes  No | 149 29 | 83.7 16.3 |

* Some psychiatrists had more than one workplace
** Other workplaces: prison or forensic psychiatric centers, psychiatric and psychosocial rehabilitation centers, psychiatric mobile crisis or response teams, other housing and care centers for other subpopulations (e.g. students, disabled persons).
