# Supplementary material for: The engagement of psychiatrists in the assessment of euthanasia requests from psychiatric patients in Belgium: a survey study
Source: BMC Psychiatry. 2020 Aug 8;20:400. doi: 10.1186/s12888-020-02792-w (PMC7414658; doi:10.1186/s12888-020-02792-w)
Supplement: Supplementary file 1 — Additional file 1. [file 12888_2020_2792_MOESM1_ESM.zip › BOX 1_Summary of Legal Criteria .docx]

Box 1. Legal criteria for euthanasia in adult patients Belgium

| Legal criteria for euthanasia in Belgium |
| --- |
| Euthanasia is allowed if the patient:   1. is legally competent and conscious at the moment of the euthanasia request; 2. has made a voluntary, well-considered, and repeated request, that is not the result of any external pressure; and 3. is in a medical situation, without prospect of improvement, of constant and unbearable *physical and/or psychological* suffering  i) that cannot be alleviated; and  ii) that results from a serious and incurable condition caused by accident or illness. |
| Decision-making procedure |
| The attending physician must:   1. inform the patient about her health condition and life expectancy; 2. discuss with the patient her euthanasia request and any therapeutic and palliative options still remaining and their consequences; 3. be certain that all substantive criteria have been met, including the patient’s constant and unbearable suffering that cannot be alleviated and the durable nature of the request, and to this end;    1. have several conversations with the patient, spread out over a reasonable period of time, taking into account the progress of the patient’s condition;    2. consult a second physician,       1. who must be independent and competent to give an opinion on the condition concerned;       2. who must review the medical record and examine the patient; and       3. who must ascertain the patient’s constant and unbearable suffering that cannot be alleviated;    3. In case the patient is a terminally ill minor or not-terminally ill adult: consult a third physician,       1. who must be independent and a psychiatrist;       2. who must review the medical record and examine the patient; and       3. who must ascertain the constant and unbearable suffering that cannot be alleviated, and the voluntary, well-considered, and repeated nature of the euthanasia request;    4. in case the patient is a terminally ill minor:   consult a specialist in child and/or adolescence psychiatry   - 1. if there is a nursing team that has regular contact with the patient, discuss the request with that team or with members of that team;   2. in case of adult patients: if the patient so desires, discuss the request with the relatives appointed by the patient;  in case of minors, discuss the request with the patient’s guardian and ascertain the latter’s approval; and   3. ascertain that the patient has had the opportunity to discuss the request with the persons whom she designates. |
