# Supplementary material for: The engagement of psychiatrists in the assessment of euthanasia requests from psychiatric patients in Belgium: a survey study
Source: BMC Psychiatry. 2020 Aug 8;20:400. doi: 10.1186/s12888-020-02792-w (PMC7414658; doi:10.1186/s12888-020-02792-w)
Supplement: Supplementary file 1 — Additional file 1. [file 12888_2020_2792_MOESM1_ESM.zip › BOX 2_Glossary_Survey_English(1).docx]

**BOX 2 : Glossary**

**Euthanasia**:
The act of a physician, intentionally terminating a patient’s life at her explicit request by means of administering a lethal dose of drugs to this patient OR supplying a lethal dose of drugs to this patient who then self-administers it (i.e. medical assistance in dying).
**Clarification of a euthanasia request**:

During the clarification of a euthanasia request, the physician will examine whether a patient is eligible for euthanasia (i.e. whether the patient and her explicit euthanasia request is in accordance with all legal and/or other due care criteria).
**Treating physician:**

The treating physician of the patient focuses on the treatment of the patient’s psychopathology. This physician can also be involved in the clarification of the patient’s euthanasia request and/or can act as performing physician. **Performing physician**:
This physician can perform the act of euthanasia independently, with the assistance of a colleague physician, or can just be present during the act of euthanasia, for example by preparing and/or supplying a lethal dose of drugs that the patient then self-administers (i.e. medical assistance in dying).
**Preliminary advising physician**:
The physician who is asked to give informal advice on a few specific criteria concerning the patient’s explicit euthanasia request. This advice may include assessment or evaluation of e.g. the patient’s mental competence, the presence/exclusion of acute depression, etc.

**Procedural advising physician**:
The physician who, for the purpose of the euthanasia procedure and at the request of the performing physician, is asked to examine whether a patient is eligible for euthanasia (e.g. if the legal and clinical due care criteria are fulfilled) and is obliged to report the results of this examination in writing to the performing physician.
