# Supplementary material for: The engagement of psychiatrists in the assessment of euthanasia requests from psychiatric patients in Belgium: a survey study
Source: BMC Psychiatry. 2020 Aug 8;20:400. doi: 10.1186/s12888-020-02792-w (PMC7414658; doi:10.1186/s12888-020-02792-w)
Supplement: Supplementary file 1 — Additional file 1. [file 12888_2020_2792_MOESM1_ESM.zip › Supplemental Material_General Questionnaire_English.pdf]

General Questionnaire

Psychiatrists and trainees in psychiatry's Attitudes, Encounters with, and Engagement towards Psychiatric Euthanasia Requests and Procedures

Part 1: Some professional and personal questions in general

1. During the past 12 months, have you worked as psychiatrist (or trainee) with adult patients? ☐ Yes ☐ No

2. Have you worked (more answer options plausible):

☐ In a private or Group Practice
☐ In a Psychiatric Hospital Care
☐ In a Community Mental HealthCare Center
☐ In a Psychiatric Nursing Home
☐ In a Psychiatric Home Care
☐ In a Sheltered housing facility
☐ Other, namely:……

3. How many years are or have you been professionally active as psychiatrist, including as trainee in psychiatry?

☐ Less than 5 years ☐ 6 - 10 years ☐ 11 - 20 years ☐ More than 20 years

4. Have you ever received special training in palliative and/or other end of life care? ☐ Yes ☐ No

5. Do you feel sufficiently competent to be involved in euthanasia procedures as psychiatrist (or trainee)? ☐ Yes ☐ No

6. What is your age? ☐ Younger than 30 ☐ 30 - 40 years ☐ 41 - 60 years ☐ Older than 60 years

7. What is your sex? ☐ Male ☐ Female ☐ X

Part 2: Statements

8. *Could you please indicate to what extent you agree or disagree with the following 13 statements. This always concerns your own personal opinion, not what is or is not permitted by law. Hence, there are no right or wrong answers.*
Note: The statements regarding psychiatric patients only concern ADULT patients, PREDOMINANTLY suffering from psychiatric conditions as underlying motive for requesting euthanasia.

	Totally disagree				Totally agree
Euthanasia should only be legally allowed for the terminally ill.	☐	☐	☐	☐	☐
Euthanasia should be legally allowed for the non-terminally ill, but only when based on somatic illnesses.	☐	☐	☐	☐	☐
Euthanasia should remain legally allowed for patients with psychiatric illnesses.	☐	☐	☐	☐	☐
Euthanasia assessment in psychiatric patients is compatible with a psychotherapeutic relationship.	☐	☐	☐	☐	☐
In psychiatric patients, physician-assisted suicide (physician provides the lethal drugs to the patient who then self-administers it) is more acceptable than euthanasia (physician administers the lethal drugs to the patient).	☐	☐	☐	☐	☐
A psychiatric patient can find herself in a medically hopeless situation.	☐	☐	☐	☐	☐
A psychiatric patient can suffer unbearably.	☐	☐	☐	☐	☐
For a psychiatric patient, a lack of reasonable treatment perspectives can exist.	☐	☐	☐	☐	☐
Euthanasia is an acceptable alternative to prevent for suicide.	☐	☐	☐	☐	☐
During the assessment of a psychiatric patient's euthanasia request, potentially effective therapeutic treatment options should be taken into account.	☐	☐	☐	☐	☐
During the assessment of a psychiatric patient's euthanasia request, the focus should not only be placed on the patient's medical condition, but also on the patient's whole life context.	☐	☐	☐	☐	☐
A psychiatric patient's death request can be well considered, and not only considered as a symptom of the patient's psychopathology.	☐	☐	☐	☐	☐
In some cases, there is mention of overly permissive approaches towards euthanasia in psychiatric patients.	☐	☐	☐	☐	☐

Part 3: The following questions gauge your engagement in ADULT patients' euthanasia requests that are PRIMARILY BASED on the suffering experiences CAUSED by one or more psychiatric disorder(s), other than dementia.

9. Have you ever refused to be actively engaged **as a treating physician** in order to have a psychiatric patient's explicit euthanasia request clarified?	☐ Yes ☐ No
10. What was your main motive for (non-) refusal?	……………………………………………………………………………………… ………………………………………………………………………………………

11. During your career, have you been actively engaged in one or more roles concerning explicitly expressed euthanasia requests of adult patients with a psychiatric disorder(s)? (You may tick more than 1 box)

☐ No, not in any role

☐ Yes, as **treating physician**, who refers their own patient to a colleague-physician for further clarification/advice

☐ Yes, as **attending physician**, engaged in the clarification of a euthanasia request of **my own patient**

☐ Yes, as **attending physician**, engaged in the clarification of a euthanasia request of a **colleague-physician's patient**

☐ Yes, as **preliminary advising physician** concerning a partial aspect (e.g. ruling out the existence of an acute depression, assessing mental competence)

☐ Yes, as **procedural advising physician** concerning the legally required 1st or 2nd advice

☐ Yes, as **performing physician**, when being present at, assisting in or carrying out the act of euthanasia in **my own patient**

☐ Yes, as **performing physician**, when being present at, assisting in or carrying out the act of euthanasia in a **colleague's patient**

☐ Yes, in another role:……

12. During the past 12 months, how often have you been engaged in euthanasia procedures concerning psychiatric patients (in whatever role)?

☐ 0 patients	☐ 1-2 patients	☐ 3-5 patients	☐ 5-9 patients	☐ 10-20 patients	☐ > 20 patients

13. During the past 12 months, how often have you given a **positive and/or negative advice** or **refused to give an advice**?

Positive advice	☐ Not applicable	☐ 1-2 patients	☐ 3-5 patients	☐ More than 5 patients
Negative advice	☐ Not applicable	☐ 1-2 patients	☐ 3-5 patients	☐ More than 5 patients
Refused to give advice	☐ Not applicable	☐ 1-2 patients	☐ 3-5 patients	☐ More than 5 patients

14. **During the past 5 years**, for how many psychiatric patients have you been engaged as **performing physician**?

☐ 0 patients	☐ 1-2 patients	☐ 3-5 patients	☐ More than 5 patients

15. Would you consider to actively engage in one or more roles concerning explicitly expressed euthanasia requests of adult patients with (a) psychiatric disorder(s)? (You may tick more than 1 box)

☐ No, not in any role

☐ Yes, as **treating physician**, who refers their own patient to a colleague-physician for further clarification/advice

☐ Yes, as **attending physician**, engaged in the clarification of a euthanasia request of **my own patient**

☐ Yes, as **attending physician**, engaged in the clarification of a euthanasia request of a **colleague-physician's patient**

☐ Yes, as **preliminary advising physician** concerning a partial aspect (e.g. ruling out the existence of an acute depression, assessing mental competence)

☐ Yes, as **procedural advising physician** concerning the legally required 1st or 2nd advice

☐ Yes, as **performing physician**, when being present at, assisting in or carrying out the act of euthanasia in **my own patient**

☐ Yes, as **performing physician**, when being present at, assisting in or carrying out the act of euthanasia in a **colleague's patient**

☐ Yes, in another role:……

16. Have you ever engaged an **external consultation team**, specialised in the clarification of euthanasia requests (e.g. ULteam, Vonkel) in the euthanasia procedure of a psychiatric patient?

☐ No, and I would never consider it<br>☐ No, but I might consider it	☐ Yes, namely: ……………………………………………………………………..<br>………………………………………………………………………………………
17. For what reason would or wouldn't you engage such an external consultation team?	………………………………………………………………………………………<br>………………………………………………………………………………………

Would you like to clarify or explain some of your answers to the abovementioned questions?

……

……
