# Supplementary material for: The engagement of psychiatrists in the assessment of euthanasia requests from psychiatric patients in Belgium: a survey study
Source: BMC Psychiatry. 2020 Aug 8;20:400. doi: 10.1186/s12888-020-02792-w (PMC7414658; doi:10.1186/s12888-020-02792-w)
Supplement: Supplementary file 1 — Additional file 1. [file 12888_2020_2792_MOESM1_ESM.zip › Supplemental Material_General Questionnaire_French.pdf]

Questionnaire général : attitudes envers l'euthanasie et expériences dans ce domaine de la part des psychiatres (assistants)

Volet 1 : Questions générales, professionnelles et personnelles	
1. Au cours de ces 12 derniers mois, avez-vous travaillé comme psychiatre (assistant(e)) avec des patients adultes ?	☐ Oui ☐ Non
2. Au cours de cette période, avez-vous travaillé comme psychiatre (plusieurs réponses possibles) :	
☐ En pratique privée (de groupe)	☐ En Maison de Soins psychiatrique (MSP)
☐ En milieu hospitalier	☐ Accompagnement psychiatrique à domicile (APD)
☐ En Centre de Santé mentale (CSM)	☐ Initiative d'Habitation protégée (IHP)
☐ Autre, c.-à-d. …………………………………………………………………………	
3. Depuis combien d'années travaillez-vous ou avez-vous travaillé comme psychiatre, y compris en tant qu'assistant(e) ?	
☐ moins de 5 ans ☐ entre 6 et 10 ans ☐ entre 11 et 20 ans ☐ plus de 20 ans	
4. Avez-vous suivi une formation spécialisée en soins palliatifs et/ou autres soins de fin de vie ?	☐ Oui ☐ Non
5. Estimez-vous disposer des compétences nécessaires pour être associé(e) à une procédure d'euthanasie en tant que psychiatre ?	☐ Oui ☐ Non
6. Votre âge ?	☐ moins de 30 ans ☐ 30-40 ans ☐ 41-60 ans ☐ plus de 60 ans
7. Votre genre ?	☐ Homme ☐ Femme ☐ X

Volet 2 : Affirmations					
8. *Indiquez dans quelle mesure vous êtes d'accord avec les 13 affirmations suivantes. Il s'agit à chaque fois de votre opinion personnelle et non de ce qui est ou n'est pas autorisé par la loi. Il n'y a donc pas de bonnes ni de mauvaises réponses.* Note : les affirmations à propos de patients psychiatriques concernent uniquement les patients présentant un syndrome psychiatrique en tant que pathologie sous-jacente.					
	Pas du tout d'accord				Tout à fait d'accord
La loi devrait uniquement autoriser l'euthanasie pour les patients en phase terminale.	☐	☐	☐	☐	☐
La loi devrait autoriser l'euthanasie pour les patients en phase non-terminale, mais uniquement en cas d'affection somatique.	☐	☐	☐	☐	☐
L'euthanasie devrait rester autorisée par la loi pour les patients atteints d'une pathologie psychiatrique.	☐	☐	☐	☐	☐
L'euthanasie des patients psychiatriques est compatible avec une relation de soins psychiatrique.	☐	☐	☐	☐	☐
Pour les patients psychiatriques, le suicide médicalement assisté (le patient s'auto-administre la dose létale en présence du médecin et accompagné par lui) me semble plus acceptable que l'euthanasie (le médecin administre la dose létale au patient).	☐	☐	☐	☐	☐
Un patient psychiatrique peut être dans une situation médicalement sans issue.	☐	☐	☐	☐	☐
Un patient psychiatrique peut être dans un état de souffrance intolérable.	☐	☐	☐	☐	☐
Il est possible que des perspectives de traitement raisonnables fassent défaut pour un patient psychiatrique.	☐	☐	☐	☐	☐
L'euthanasie est une alternative acceptable pour prévenir le suicide.	☐	☐	☐	☐	☐
Lors de la dépistage d'une demande d'euthanasie, la possibilité de futures avancées thérapeutiques effectives doit être prise en compte.	☐	☐	☐	☐	☐
La dépistage d'une demande d'euthanasie ne devrait pas se focaliser sur la situation médicale, mais devrait aussi prendre en compte les conditions de vie générales du patient.	☐	☐	☐	☐	☐
La volonté de mourir d'un patient psychiatrique peut être mûrement réfléchie et pas uniquement un symptôme d'une pathologie sous-jacente.	☐	☐	☐	☐	☐
Dans certains cas, la possibilité pour un patient psychiatrique de retourner à une demande d'euthanasie est traitée trop à la légère.	☐	☐	☐	☐	☐

Volet 3 : Les questions suivantes concernent vos expériences avec des demandes d'euthanasie de patients ADULTES DECOULANT PRINCIPALEMENT d'une ou de plusieurs pathologie(s) psychiatrique(s) affectant le patient.	
9. **En tant que médecin traitant** d'un patient psychiatrique, vous est-il arrivé de refuser d'être activement associé(e) à la dépistage de sa demande d'euthanasie explicite ?	☐ Oui<br>☐ Non

10. Quelle(s) étai(en)t votre/vos motivation(s) ?	....<br>..

11. Au cours de votre carrière de psychiatre, avez-vous été associé(e) à la demande d'euthanasie explicite d'un patient adulte atteint principalement d'une ou de plusieurs pathologie(s) psychiatrique(s) ? *(Cocher plusieurs options est possible)*

☐ Non, cela ne m'est jamais arrivé **(→ question 15)**
☐ Oui, en tant que médecin traitant ayant **renvoyé** son propre patient à un **confrère** pour la dépistage d'une demande d'euthanasie.
☐ Oui, en tant que médecin traitant s'étant chargé de la dépistage d'une demande d'euthanasie d'un **propre patient**.
☐ Oui, en tant que médecin s'étant chargé de la dépistage d'une demande d'euthanasie d'un **patient d'un confrère**.
☐ Oui, en tant que **médecin consulté au préalable** à propos d'un élément partiel (par ex. exclusion d'un état dépressif aigu, évaluation de la capacité mentale à manifester sa volonté).
☐ Oui, en tant que premier ou second **médecin consulté**, comme légalement requis par la **procédure** d'euthanasie.
☐ Oui, en tant que médecin déclarant ayant procédé à l'euthanasie d'un **propre patient**, y ayant apporté son assistance ou y ayant été présent(e).
☐ Oui, en tant que médecin déclarant ayant procédé à l'euthanasie d'un **patient d'un confrère**, y ayant apporté son assistance ou y ayant été présent(e).
☐ Oui, dans une autre fonction, c.-à-d. ....

12. Au cours de ces 12 derniers mois, à combien de demandes d'euthanasie de tels patients avez-vous été associé(e) (dans n'importe quel rôle professionnel) ?

☐ aucun patient **(→ question 14)**	☐ 1-2 patients	☐ 3-5 patients	☐ 5-9 patients	☐ 10-20 patients	☐ > 20 patients

13. Au cours de ces 12 derniers mois, combien de fois avez-vous fourni un **avis favorable ou défavorable** ou **refusé** de fournir un avis ?

Avis favorable	☐ pas d'application	☐ 1-2 patients	☐ 3-5 patients	☐ plus de 5 patients
Avis défavorable	☐ pas d'application	☐ 1-2 patients	☐ 3-5 patients	☐ plus de 5 patients
Refus d'avis	☐ pas d'application	☐ 1-2 patients	☐ 3-5 patients	☐ plus de 5 patients

14. Pour combien de patients psychiatriques avez-vous agi en tant que médecin déclarant au cours de ces 5 dernières années ?

☐ aucun patient	☐ 1-2 patients	☐ 3-5 patients	☐ plus de 5 patients

15. Envisagez-vous d'assumer à l'avenir une ou plusieurs fonctions dans le cadre de demandes d'euthanasie concrètes de patients adultes atteints d'une ou de plusieurs pathologie(s) psychiatrique(s) ? *(cocher plusieurs options est possible)*

☐ Oui, en tant que médecin traitant renvoyant l'un de ses propres patients à un confrère pour la suite du processus.
☐ Oui, en tant que médecin traitant se chargeant de la dépistage d'une demande d'euthanasie d'un **propre patient**.
☐ Oui, en tant que médecin se chargeant de la dépistage d'une demande d'euthanasie du **patient d'un confrère**.
☐ Oui, en tant que **médecin consulté au préalable** à propos d'un élément partiel (par ex. exclusion d'un état dépressif aigu, évaluation de la capacité mentale à manifester sa volonté).
☐ Oui, en tant que premier ou second **médecin consulté**, comme légalement requis par la **procédure** d'euthanasie.
☐ Oui, en tant que médecin déclarant procédant à l'euthanasie d'un **propre patient**, y apportant son assistance ou y étant présent(e).
☐ Oui, en tant que médecin déclarant procédant à l'euthanasie d'un **patient d'un confrère**, y apportant son assistance ou y étant présent(e).
☐ Oui, dans une autre fonction, c.-à-d. ....
☐ Non, dans aucune fonction.

16. Vous est-il arrivé d'associer une **équipe de consultation extérieure**, spécialisée dans la dépistage des demandes d'euthanasie (par exemple ULteam), à la procédure d'euthanasie d'un patient psychiatrique ?

☐ Non, et je ne le ferais jamais	☐ Oui, c'est-à-dire : ....
☐ Non, mais il est concevable que je le fasse un jour	....

17. Pour quelle(s) raison(s) feriez-vous appel ou non à une équipe de consultation extérieure ?	....<br>...

Souhaitez-vous ajouter des précisions ou des commentaires ?

....
....
....
