# Supplementary material for: The engagement of psychiatrists in the assessment of euthanasia requests from psychiatric patients in Belgium: a survey study
Source: BMC Psychiatry. 2020 Aug 8;20:400. doi: 10.1186/s12888-020-02792-w (PMC7414658; doi:10.1186/s12888-020-02792-w)
Supplement: Supplementary file 1 — Additional file 1. [file 12888_2020_2792_MOESM1_ESM.zip › Supplemental Material_Optional Questionnaire_English.pdf]

Facultative Questionnaire : the most recent concrete experience

The questions below relate to your most recent experience with a completed euthanasia procedure (regardless of its final outcome) of an ADULT patient, PREDOMINANTLY suffering from a psychiatric condition, other than dementia, in the past 12 months.

1. What was your role (You may tick more than 1 box)

☐ Treating physician of the patient and their psychopathology
☐ Clarification of a euthanasia request of **my own patient**
☐ Clarification of a euthanasia request of a **colleague-physician's patient**
☐ Preliminary advising physician
☐ Procedural advising physician
☐ Performing physician

2. How long di dit take for the decision to be made, from the first request for euthanasia until the final decision (indicate the number of weeks/months/years)	....
3. How long had the patient been in treatment before he/she consulted you and requested euthanasia? (indicate the number of weeks/months/years)	....

4. Was the patient undergoing any psychotherapeutic treatment(s) at the time of the first consultation with regard to his/her euthanasia request? (You may tick more than 1 box)

☐ No active treatment
☐ Yes, psychotropics
☐ Yes, psychotherapy
☐ Other therapeutic interventions,
☐ Yes, other medical drugs
☐ Yes, neurochirurgical treatment

....

....

5. What was the patient's main clinical pathology ? (You may tick and fill in more than 1 box)

☐ Psychiatric disorders :..
☐ Personality disorders : ....
☐ Somatic co-diagnoses :...

6. What were the patient's motives for requesting euthanasia? (You may tick more than 1 box)

☐ Feelings of depression
☐ Stalled on many life domains (work/relationships/...)
☐ No perspective for improvement
☐ No purpose (left) in life
☐ Loneliness
☐ No (longer) wanting to be a burden
☐ Existential suffering (suffering on life itself, meaninglessness)
☐ Gradual deterioration
☐ Fear of suicide
☐ Disability/Immobility
☐ Loss of autonomy, control over own life
☐ Loss of dignity
☐ Total exhaustion
☐ No quality of life, only in 'survival mode'
☐ Other motives : ....

....

7. In your opinion, what were the 2 main reasons for the patient to request euthanasia?

....

....

8. Did you consult one or more physicians/caregivers/institutions during the decision-making process? (You may tick more than 1 box)

☐ No
☐ Yes, the patient's general practitioner
☐ Yes, Independent colleague-psychiatrist(s)
☐ Yes, other physicians of the patient
☐ Yes, Independent trained and experienced LEIF-physician(s)
☐ Yes, Independent physicians of specialised EOL centers
☐ Yes, nurse(s)
☐ Yes, the ethics committee
☐ Yes, another internal advisory committee
☐ Yes, psychologist(s)
☐ Yes, a palliative care team
☐ Yes, (Psycho-)Social service(s)
☐ Yes, other, namely : ....

9. Was the patient's family and/or friends involved? (You may tick more than 1 box)

☐ No, although the patient did have family or friends
☐ No, patient did not have family or friends
☐ Yes, during the euthanasia procedure
☐ Yes, during and after the euthanasia procedure

10. In your opinion, in this patient's case, was there any question of...

Mental competency	☐ Yes	☐ No
Incurable disorder	☐ Yes	☐ No
Unbearable suffering	☐ Yes	☐ No
Medical futility	☐ Yes	☐ No

No reasonable therapeutic options left ☐ Yes ☐ No
Voluntary, sustained and repeated request ☐ Yes ☐ No

11. How difficult was it for you to assess the following criteria?

	None				Plenty
Mental competency	☐	☐	☐	☐	☐
Incurable disorder	☐	☐	☐	☐	☐
Unbearable suffering	☐	☐	☐	☐	☐
Medical futility	☐	☐	☐	☐	☐
No reasonable therapeutic options left	☐	☐	☐	☐	☐
Voluntary, sustained and repeated request	☐	☐	☐	☐	☐
Other criteria, namely …………………………………………………………………………………………	☐	☐	☐	☐	☐

12. In the course of the euthanasia procedure, did you face any pressure as indicated below?

Patient requesting euthanasia under pressure from others ☐ Yes ☐ No
Pressure from the patient to approve euthanasia ☐ Yes ☐ No
Pressure from patient's family or friends to approve euthanasia ☐ Yes ☐ No
Pressure from patient's family or friends to reject the euthanasia request ☐ Yes ☐ No
Pressure from colleagues to reject the euthanasia request ☐ Yes ☐ No
Pressure from colleagues to approve euthanasia ☐ Yes ☐ No
Pressure from the care institute to reject the euthanasia request ☐ Yes ☐ No
Pressure from the care institute to approve euthanasia ☐ Yes ☐ No

13. During the euthanasia procedure, did you encounter the following experiences?

High emotional burden for yourself ☐ Yes ☐ No
A lowered risk of suicide with the patient ☐ Yes ☐ No
New therapeutic opportunities with the patient ☐ Yes ☐ No
Re-establishment of relationships (patient and significant others) ☐ Yes ☐ No
Fellow patients also requesting euthanasia ☐ Yes ☐ No

14. What was the nature of the advice/advices provided as part of the request for euthanasia?

☐ Don't know ☐ … positive advices ☐ … negative advices
☐ No advices were obtained

15. Did the patient die by euthanasia?

☐ Don't know ➔ question 17
☐ Yes ➔ question 16
☐ No, the procedure is still ongoing ➔ question 17
☐ No, the patient had withdrawn the request *without external pressure* ➔ question 17
☐ No, the patient had withdrawn the request *under external pressure* ➔ question 17
☐ No, the patient died otherwise ➔ question 17
☐ No, because ……➔ question 17

16. Did you attend the euthanasia?

☐ Yes, and I administered the lethal substance myself (with or without the assistance of an experienced colleague).
☐ Yes, and I prepared the substance, which was then administered to the patient by another caregiver.
☐ Yes, and I've prepared the substance, which the patient self-administered.
☐ Yes, but I did not prepare or administer the substance
☐ No

17. Did you seek emotional support for yourself during or after the euthanasia procedure? (multiple answers possible)

☐ No | ☐ Yes, inner personal circle | ☐ Yes, colleagues | ☐ Yes, external professional help | ☐ Oui, others

18. Has this case influenced your attitude towards future euthanasia requests and procedures? ☐ Yes ☐ No ➔ question 20

19. In what way has it changed your attitude? ……………………………………………………………………………………
……………………………………………………………………………………

20. Would you like to add any clarification or comments about this particular case?

……
……
