# Supplementary material for: The engagement of psychiatrists in the assessment of euthanasia requests from psychiatric patients in Belgium: a survey study
Source: BMC Psychiatry. 2020 Aug 8;20:400. doi: 10.1186/s12888-020-02792-w (PMC7414658; doi:10.1186/s12888-020-02792-w)
Supplement: Supplementary file 1 — Additional file 1. [file 12888_2020_2792_MOESM1_ESM.zip › Supplemental Material_Optional Questionnaire_French-1.pdf]

Volet facultatif : votre expérience la plus récente de l'euthanasie

Les questions ci-dessous concernent votre expérience la plus récente d'une procédure d'euthanasie achevée (indépendamment de l'issue finale) d'un patient atteint d'une pathologie psychiatrique au cours de ces 12 derniers mois.

1. Quelle était votre fonction ? (cocher plusieurs options est possible)

☐ Médecin traitant du patient et de sa psychopathologie
☐ Clarification de la demande d'euthanasie d'un propre patient
☐ Clarification de la demande d'euthanasie du patient d'un confrère
☐ Médecin consulté au préalable
☐ Médecin consulté dans le cadre de la procédure
☐ Médecin déclarant

2. Combien de temps a duré la prise de décision, depuis la demande d'euthanasie jusqu'à la décision finale (indiquez le nombre de semaines/mois/années) ....

3. Depuis combien de temps le patient était-il en traitement avant qu'il vous consulte et soumette une demande d'euthanasie? (indiquez le nombre de semaines/mois/années) ....

4. Le patient suivait-il un ou des traitement(s) psychothérapeutique(s) au moment de la première consultation dans le cadre de l'euthanasie? (cocher plusieurs options est possible)

☐ Non, le patient ne suivait aucun traitement
☐ Oui, des substances psychotropes
☐ Oui, des entretiens thérapeutiques
☐ Oui, d'autres médicaments
☐ Oui, un traitement neurochirurgical
☐ Autres interventions, c.-à-d. ....
....

5. Quelle était la principale pathologie du patient ? (cocher et compléter plusieurs options est possible)

☐ Trouble clinique, c.-à-d. ....
☐ Trouble de la personnalité, c.-à-d. ....
☐ Affection somatique, c.-à-d. ....

6. Quelles étaient les raisons données par le patient pour demander l'euthanasie ? (plusieurs réponses possibles)

☐ Sentiments dépressifs
☐ Échec dans plusieurs domaines de la vie (travail/ vie amoureuse/…)
☐ Aucune perspective d'amélioration
☐ Pas/plus de but dans la vie
☐ Solitude
☐ Ne pas/plus vouloir être un poids pour l'entourage
☐ Souffrance existentielle (souffrir de la vie, manque de sens)
☐ Détérioration progressive
☐ Peur du suicide
☐ Invalidité/immobilité
☐ Perte d'autonomie, du contrôle de sa propre vie
☐ Perte de dignité
☐ Épuisement total
☐ Aucune qualité de vie, juste « survivre »
☐ Autre, c.-à-d. ....
....

7. À votre avis, quelles étaient les 2 principales raisons pour que le patient demande l'euthanasie ?

....
....

8. Avez-vous consulté un(e) ou plusieurs médecins/soignants/instances au cours de la prise de décision ? (cocher plusieurs options est possible)

☐ Non
☐ Oui, le(s) médecin(s) généraliste(s) du patient
☐ Oui, un/autre(s) médecin(s) du patient
☐ Oui, des confrères psychiatres indépendants
☐ Oui, un/des médecin(s) EOL-LEIF indépendant(s)
☐ Oui, un/des médecin(s) indépendant(s) d'une équipe de consultation sur la fin de vie
☐ Oui, infirmier(s)/infirmière(s)
☐ Oui, commission d'éthique
☐ Oui, autre commission consultative interne
☐ Oui, un/des psychologue(s)
☐ Oui, une équipe palliative
☐ Oui, service (psycho-)social
☐ Oui, autre(s), c.-à-d. ....

9. Y a-t-il eu concertation avec la famille et/ou les amis du patient ? (plusieurs réponses possibles)

☐ Non, pas de membres de la famille ni d'amis associés au processus
☐ Non, le patient n'avait pas de famille ni d'amis
☐ Oui, au cours de la procédure d'euthanasie
☐ Oui, après la fin de la procédure d'euthanasie

10. À votre avis, dans le cas de ce patient était-il question de…

Compétence mentale (capacité de discernement)	☐ Oui	☐ Non
Affection incurable	☐ Oui	☐ Non
Souffrance insupportable	☐ Oui	☐ Non

Situation médicale sans issue	☐ Oui	☐ Non
Absence de perspectives thérapeutiques raisonnables	☐ Oui	☐ Non
Demande formuleé de manière volontaire, réfléchie et répétée	☐ Oui	☐ Non

11. Quelle difficulté avez-vous eu pour évaluer les critères ci-dessous ?

	Aucune				Immense
Compétence mentale (capacité de discernement)	☐	☐	☐	☐	☐
Affection incurable	☐	☐	☐	☐	☐
Souffrance insupportable	☐	☐	☐	☐	☐
Situation médicale sans issue	☐	☐	☐	☐	☐
Absence de perspectives thérapeutiques raisonnables	☐	☐	☐	☐	☐
Demande formuleé de manière volontaire, réfléchie et répétée	☐	☐	☐	☐	☐
Autres, c.-à-d.	☐	☐	☐	☐	☐

12. Au cours de la procédure d'euthanasie, avez-vous été confronté(e) à des pressions telles qu'indiquées ci-dessous ?

Patient demandant l'euthanasie sous la pression de tiers	☐ Oui	☐ Non
Pressions du patient pour décider de l'euthanasie (avis/exécution)	☐ Oui	☐ Non
Pressions des proches pour décider de l'euthanasie (avis/exécution)	☐ Oui	☐ Non
Pressions des proches pour rejeter la demande d'euthanasie	☐ Oui	☐ Non
Pressions de confrères pour rejeter la demande d'euthanasie	☐ Oui	☐ Non
Pressions de confrères pour décider de l'euthanasie (avis/exécution)	☐ Oui	☐ Non
Pression d'une institution de soins pour rejeter la demande d'euthanasie	☐ Oui	☐ Non
Pression d'une institution de soins pour décider de l'euthanasie (avis/ exécution)	☐ Oui	☐ Non

13. Au cours de la procédure d'euthanasie, avez-vous rencontré les éléments suivants ?

Charge émotionnelle importante pour vous-même	☐ Oui	☐ Non
Nouvelles possibilités thérapeutiques pour le patient	☐ Oui	☐ Non
Baisse du risque de suicide chez le patient	☐ Oui	☐ Non
Réhabilitation des rapports entre le patient et ses proches	☐ Oui	☐ Non
Autres patients demandant également l'euthanasie	☐ Oui	☐ Non

14. Quelle a été la nature de l'avis/des avis fournis dans le cadre de la demande d'euthanasie ? (plusieurs réponses possibles)

☐ Pour finir, aucun avis n'a été fourni ☐ ... avis favorables ☐ ... avis défavorables

☐ Je n'en ai pas été informé(e)

15. Le patient est-il décédé par euthanasie ?

☐ Je ne sais pas ➔ question 17

☐ Oui ➔ question 16

☐ Non, la procédure d'euthanasie n'a pas été menée à bout ➔ question 17

☐ Non, le patient a retiré la demande d'euthanasie *sans pressions de tiers* ➔ question 17

☐ Non, le patient a retiré la demande d'euthanasie *sous la pression de tiers* ➔ question 17

☐ Non, le patient est décédé d'une autre cause ➔ question 17

☐ Non, car ....➔ question 17

16. Avez-vous assisté à l'euthanasie ?

☐ Oui, et j'ai moi-même administré la substance létale (éventuellement avec l'assistance d'un confrère expérimenté)

☐ Oui, et j'ai préparé la substance, qui a ensuite été administrée au patient par un(e) autre soignant(e)

☐ Oui, et j'ai préparé la substance, que le patient s'est auto-administré

☐ Oui, mais je n'ai pas préparé ni administré la substance

☐ Non

17. Avez-vous cherché un soutien émotionnel pour vous-même pendant ou après la procédure d'euthanasie ? (plusieurs réponses possibles)

☐ Non | ☐ Oui, en privé | ☐ Oui, auprès de confrères | ☐ Oui, auprès de professionnels externes | ☐ Oui, autres

18. Ce cas a-t-il influencé votre attitude envers des demandes futures ? ☐ Oui ☐ Non➔ question 20

19. De quelle manière a-t-il modifié votre attitude ? ....

....

20. Souhaitez-vous ajouter des précisions ou des commentaires à propos de ce cas particulier ?

....

....

....
